# POLQ suppresses genome instability and alterations in DNA repeat tract lengths

**DOI:** 10.1093/narcan/zcac020

**Published:** 2022-06-29

**Authors:** Kate Liddiard, Alys N Aston-Evans, Kez Cleal, Eric A Hendrickson, Duncan M Baird

**Affiliations:** Division of Cancer and Genetics, School of Medicine, Cardiff University, Heath Park, Cardiff CF14 4XN, UK; Dementia Research Institute, School of Medicine, Cardiff University, Hadyn Ellis Building, Maindy Road, Cardiff CF24 4HQ, UK; Division of Cancer and Genetics, School of Medicine, Cardiff University, Heath Park, Cardiff CF14 4XN, UK; Department of Biochemistry, Molecular Biology, and Biophysics, University of Minnesota Medical School, Minneapolis, MN 55455, USA; Division of Cancer and Genetics, School of Medicine, Cardiff University, Heath Park, Cardiff CF14 4XN, UK

## Abstract

DNA polymerase theta (POLQ) is a principal component of the alternative non-homologous end-joining (ANHEJ) DNA repair pathway that ligates DNA double-strand breaks. Utilizing independent models of POLQ insufficiency during telomere-driven crisis, we found that *POLQ*^–^^/–^ cells are resistant to crisis-induced growth deceleration despite sustaining inter-chromosomal telomere fusion frequencies equivalent to wild-type (WT) cells. We recorded longer telomeres in *POLQ*^–^^/^^–^ than WT cells pre- and post-crisis, notwithstanding elevated total telomere erosion and fusion rates. *POLQ*^–^^/–^ cells emerging from crisis exhibited reduced incidence of clonal gross chromosomal abnormalities in accordance with increased genetic heterogeneity. High-throughput sequencing of telomere fusion amplicons from POLQ-deficient cells revealed significantly raised frequencies of inter-chromosomal fusions with correspondingly depreciated intra-chromosomal recombinations. Long-range interactions culminating in telomere fusions with centromere alpha-satellite repeats, as well as expansions in HSAT2 and HSAT3 satellite and contractions in ribosomal DNA repeats, were detected in *POLQ*^–^^/^^–^ cells. In conjunction with the expanded telomere lengths of *POLQ*^–^^/–^ cells, these results indicate a hitherto unrealized capacity of POLQ for regulation of repeat arrays within the genome. Our findings uncover novel considerations for the efficacy of POLQ inhibitors in clinical cancer interventions, where potential genome destabilizing consequences could drive clonal evolution and resistant disease.

## Graphical Abstract

**Graphical Abstract:**
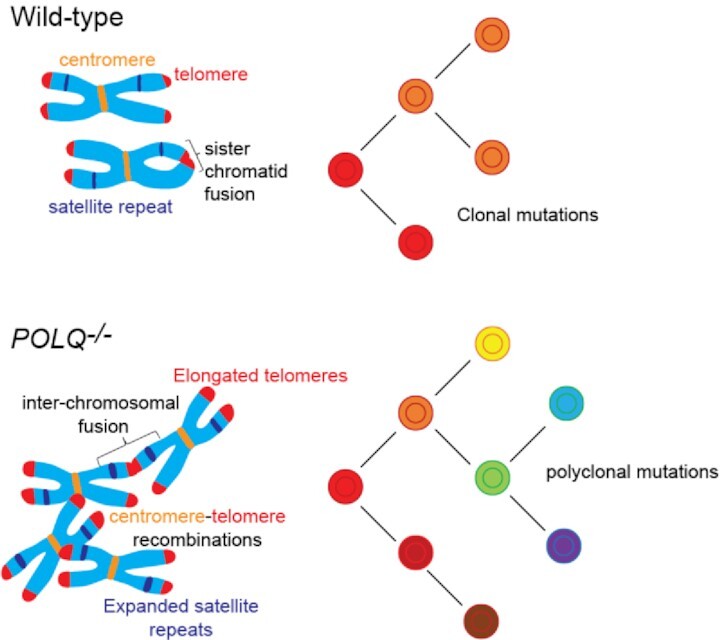
POLQ-deficient cells display altered DNA repeat tract lengths and genetic heterogeneity, but reduced intra-chromosomal telomere fusion frequencies.

## INTRODUCTION

DNA polymerase theta (POLQ) is a large (290 kDa in humans) atypical A-family polymerase integral to mammalian error-prone DNA repair (DNAR) (1,2). POLQ uniquely exhibits ATP-dependent helicase activity, while lacking the 3′–5′ exonuclease proofreading capacity that restrains translesion synthesis (3–5). In combination with lesion bypass, POLQ-mediated DNAR [theta-mediated end joining (TMEJ) (6)] produces mutations as a consequence of microhomology-dependent annealing with irrecoverable exclusion of terminal DNA sequences (7–9). Additionally, POLQ synthesizes bridging sequences through template-independent extension of single-stranded DNA (ssDNA), generating novel insertions at DNA double-strand breaks (DSBs) that interrupt genomic constitution (10–12). As such, TMEJ represents a facet of alternative non-homologous end joining (ANHEJ) identified by mutational signature as well as POLQ dependence (7,13,14). POLQ has been implicated in diverse DNA-modulating activities, including reverse transcription (15), base excision repair (BER) (16), mismatch repair (MMR) (17) and responses to replication stress (17), demonstrating the prevailing influence of this multifunctional protein (18,19). Pertinently, reciprocal interactions between POLQ, RAD51 and RPA determine the balance of ANHEJ and homologous recombination (HR) (12,20). POLQ conjointly restricts RAD51 recruitment to (20) and facilitates RPA removal (21) from resected DSBs, stimulating ANHEJ at the expense of high-fidelity HR repair. By corollary, TMEJ captures the products of unproductive HR, providing a viable post-replicative DNAR alternative when HR is inadequate. Additional inter-pathway regulation is accorded by RAD52 that constrains the polymerase function of POLQ to mitosis to suppress aberrant chromosome fusions (22). Consequently, POLQ is synthetic lethal with HR components (23,24) and tumours compromised in HR repair are especially vulnerable to POLQ inhibition (20,25). Conversely, overexpression of POLQ may confer resistance to replication stress, contributing to poor patient prognosis in cancer (26,27). The advent of novel pharmaceutical POLQ inhibitors combined with a burgeoning appreciation of the interactivity of DNAR pathways imparts renewed impetus to the management of residual and resistant disease in cancer (28,29).

NHEJ-mediated fusions between chromosome termini characterize the state of heightened genome instability termed ‘crisis’ instigated by evasion of cell cycle checkpoints following telomere damage or deprotection (30,31). Crisis can be modelled in cancer cell lines through repression of telomerase activity (32), resulting in telomere attrition as a function of cell division (33). Telomeres shortened beyond a critical threshold (34) in cells divested of effective cell cycle regulation provide insufficient defence against terminal recombinations when exposed chromosome ends become the substrates of aberrant DNAR (35). Telomere fusions promote the propagation of genome instability during crisis through deranged mitotic partition of dicentric chromosomes, resulting in genome rearrangements and copy number variations (CNVs) (36–41). Immortality is achieved through the renewal of telomere length stabilization mechanisms, including the revival of telomerase activity or homology-based telomere elongation, expedited by this state of genomic fluidity (42,43). Persistent evolution of the cancer genome is a product of processive mutagenic divergence punctuated by pervasive chromosome recombinations that may have their origins in telomere dysfunction (44–50).

We (48,51–54) and others (50,55–58) have identified DNAR components and mechanisms involved in catalysing telomere fusions and dispersing genomic instability during tumour initiation and progression. Classical DNA ligase 4-dependent non-homologous end joining (CNHEJ) and ANHEJ direct telomere fusions with distinct spectra (52,59); however, successful escape from telomere-driven crisis is contingent on DNA ligase 3-mediated ANHEJ that supports sufficient appropriate chromosomal rearrangements to arrest telomere shortening without compromising cell viability and proliferative capacity (51,60,61). As the fundamental constituent of the definitive ANHEJ pathway that is TMEJ, POLQ involvement in the fusion of dysfunctional telomeres has been previously documented (12). However, POLQ is not constitutively highly expressed in non-cancerous cells (62) and, as a single mutation, produces only moderate phenotypes in model systems (6,63). Furthermore, the potential for fractional redundancy among replicative and translesion polymerases obscures current realization of essential or exclusive functions of POLQ during crisis. Resolving the rare individual telomere fusions that arise during malignant transformation poses significant technical difficulties (Dewhurst, S.M. *et al.* bioRxiv 2020.2009.2029.318436); hence, existing research is dominated by proxy identifications based on genomic structural variations and CNVs symptomatic of fusion events (49,64,65). In this study, we address these compelling scientific and experimental challenges using our bespoke telomere fusion sequencing capacity (52,66) in association with whole genome sequencing (WGS) in two independent wild-type (WT) and POLQ-deficient human cancer cell lines transiting telomere-driven crisis. With this compound approach, we distinguished POLQ-dependent and POLQ-independent rearrangements propelling clonal diversification and crisis escape. Notably, while POLQ deficiency altered the balance and frequencies of intra- and inter-chromosomal telomere fusions, cell viability and the ultimate reactivation of telomere maintenance mechanisms were not compromised. Our data also uncover an unexpected impact of POLQ on repetitive DNA tract length fluctuations, including at telomeres, satellites and ribosomal DNA (rDNA). These findings represent novel and timely contributions to the consideration of POLQ inhibitors as cancer therapeutics (25,28,29).

## MATERIALS AND METHODS

### *POLQ* ^–/–^ human cell lines

Three independent human cell lines in which *POLQ* had been disrupted using CRISPR/Cas9 editing were employed in this study, alongside unmutated WT counterparts.

HCT116 WT and *POLQ*^–/–^ cells were generated by Professor Eric Hendrickson, University of Minnesota, and were cultured in McCoy’s 5A medium supplemented with 10% (v/v) foetal calf serum, 1 × 10^5^ IU/l penicillin, 100 mg/l streptomycin and 2 mM glutamine.

HAP1 WT and POLQ-deficient cells were generously donated by Professor Geoffrey Higgins, Oxford University, and were cultured in IMDM medium supplemented with 10% (v/v) foetal calf serum, 1 × 10^5^ IU/l penicillin, 100 mg/l streptomycin and 2 mM glutamine. These cells were determined to be diploid post-crisis, so are referred to as *POLQ*^–^^/^^–^ throughout.

RPE1-hTERT (hTERT-immortalized) WT and *POLQ*^–^^/^^–^ cells were valuably contributed by Professor Agnel Sfeir, Sloan Kettering Institute, and were cultured in DMEM medium supplemented with 10% (v/v) foetal calf serum, 1 × 10^5^ IU/l penicillin, 100 mg/l streptomycin and 2 mM glutamine.

Targeted genetic disruption of *POLQ* was confirmed by Sanger sequencing of all lines in the absence of commercially available antibodies that can reliably detect this low-abundance nuclear antigen.

Antibiotic selection agent (1 μg/ml puromycin) was also added for long-term culture of cells retrovirally transduced with a dominant-negative *hTERT* expression cassette (*DN-hTERT*) to suppress telomerase-mediated telomere lengthening (32) or puromycin resistance cassette alone (controls).

Single cell suspensions were stained with 30 μg/ml acridine orange and 100 μg/ml DAPI (4′,6-diamidine-2′-phenylindole dihydrochloride) to count viable cells using the Chemometec NC-3000 image cytometer.

### Retroviral transduction of WT and *POLQ*^–/–^ cell lines

Retroviral vectors carrying a puromycin resistance cassette with or without a dominant-negative *hTERT* expression cassette (*DN-hTERT*) were packaged in ψ-CRIP cells (gifted by Richard Mulligan, Whitehead Institute). Retroviral transduction of WT and *POLQ*^–^^/^^–^ cells was achieved in the presence of 8 μg/ml polybrene (Merck) and selection with 1 μg/ml puromycin after 24 h. Single cell clones were picked using cloning rings and population growth was monitored with regular DNA sampling for 150 days for HCT116 and 250 days for HAP1 WT and *POLQ*^–^^/^^–^ clones.

### Drug treatments

Where indicated, WT and *POLQ*^–^^/^^–^ parental cell lines were treated with 5 μM cisplatin (*cis*-diamminedichloroplatinum II; Merck Life Science) or 0.9% NaCl carrier control for 24 h.

In the experiments indicated, transfected cells were treated with 100 μM novobiocin (NVB; Selleckchem) (25) or water carrier control for 48 h, with dose replenished after 24 h.

### TALEN transfections

Subtelomere DSBs were induced at specific chromosome ends using bespoke TALEN (transcription activator-like effector nuclease) pairs. TALEN pairs targeting the 17p and 16p/21q families of telomeres have been described previously (52,53). Novel TALEN pairs targeting the human chromosome Xp subtelomere at ChrX:10571 (GRCh38 reference) were designed for this study and synthesized by LabOmics.

Endonuclease-free plasmid DNA preparations were generated for each TALEN and sequence verified ahead of transfection.

### Telomere fusion assays

Telomere fusions were amplified from HCT116, HAP1 and RPE1-hTERT WT and *POLQ*^–^^/–^ cell gDNA or fibroblast samples by multiplex long-range PCR using combinations of the subtelomere-specific primers listed in Supplementary Table S8 (30,38) and detailed in the appropriate figure legends. Replicate reactions were resolved by 0.5% TAE agarose gel electrophoresis for visualization on Southern blots using radiolabelled telomere-adjacent probes specific for each telomere end. Fusion frequency was calculated from the mean number of non-constitutive fusion amplicons revealed by Southern blotting relative to DNA input and expressed per diploid genome.

### Fusion amplicon sequencing

High-throughput high-resolution sequencing of fusion amplicons derived from crisis fibroblasts is described in our former study (66). *De novo* sequencing of telomere fusions amplified from crisis-transiting HCT116 and HAP1 WT and *POLQ*^–^^/–^ clones was conducted using 100 individual fusion PCR reactions per sample that were subsequently pooled for purification using Agencourt AMPure XP magnetic beads. HCT116 fusion amplicons were sequenced by BGI Genomics Company (150-bp paired-end BGI DNBSeq™) and HAP1 fusion amplicons were sequenced at the Wales Gene Park (150-bp paired-end Illumina HiSeq 2500).

### Whole genome sequencing

Genomic DNA phenol–chloroform extracted from pre- and post-crisis WT and *POLQ*^–^^/^^–^ clones and parental cell lines was evaluated by agarose gel electrophoresis and spectrophotometry to assess integrity and purity. Between 1 and 5 μg pure DNA for each sample underwent WGS at the BGI Genomics Company (15× coverage, 150-bp paired-end).

### Data deposition

All novel sequencing data relevant to this study have been deposited under BioProject PRJNA813416 at the SRA metadata portal.

## RESULTS

### POLQ-deficient cells display extended telomere lengths and rapid transit through telomere-driven crisis

As a prominent mediator of the ANHEJ DNAR responsible for a contingent of telomere fusions that occur during crisis, we hypothesized that the abrogation of POLQ would significantly affect clonal evolution. To investigate the capacity of POLQ-deficient cells to escape a telomere-driven crisis, we employed retroviral transduction to suppress telomerase function in two independent human cancer cell lines, HCT116 and HAP1, that had undergone CRISPR/Cas9-mediated mutation of the *POLQ* gene (Supplementary Figure S1Ai). Auxiliary experiments were conducted using an hTERT-immortalized retinal pigment epithelial cell line, RPE1-hTERT, bearing the same mutation in *POLQ* as the HCT116 line. *POLQ* mutation had minimal impact on parental cell phenotype, likely on account of the low levels of expression measured in proliferating cells (Supplementary Figure S1Aii). In addition, POLQ insufficiency did not alter cellular responses to the DNA cross-linking chemotherapeutic, cisplatin (Supplementary Figure S1Aiii). Cisplatin-treated HCT116, HAP1 and RPE1-hTERT cells all demonstrated growth deceleration concomitant with *CDKN1A* upregulation (Supplementary Figure S1Aiv) that was independent of *POLQ* status. Single telomere length analyses (STELA) in these parental lines revealed a conspicuous extension of telomere length at the unique chromosome ends of 17p (for HCT116 and RPE1-hTERT lines; HAP1 17p telomeres could not be amplified) and XpYp (for HAP1 and RPE1-hTERT lines; HCT116 possesses a truncated XpYp subtelomere) in *POLQ*^–^^/–^ cells compared with WT (Supplementary Figure S1Bi). Mean telomere lengths in POLQ-deficient cells were ∼1.3–2-fold longer than their WT counterparts prior to the establishment of the telomere crisis models. To underscore the peculiarity of this prolongation, we compared the HCT116 *POLQ*^–^^/–^ cells with a complementary array of HCT116 cell lines bearing mutations targeting related DNAR pathways (Supplementary Figure S1Bii). For all models other than the *POLQ*^–^^/^^–^, we measured a significant reduction in mean 17p telomere length for the DNAR mutant compared with the corresponding WT line (Supplementary Figure S1Biii), marking POLQ as the sole component that may be linked to telomere length regulation. Since HAP1 *POLQ*^–^^/^^–^ telomere lengths may have exceeded the threshold for accurate assessment using STELA (Supplementary Figure S1Bi), we applied the orthogonal technique of telomere restriction fragment analysis to confirm ≥1.5-fold expanded gross telomere lengths in all *POLQ*^–^^/^^–^ lines beyond those of WT cells (Supplementary Figure S1Biv).

**Figure 1.**
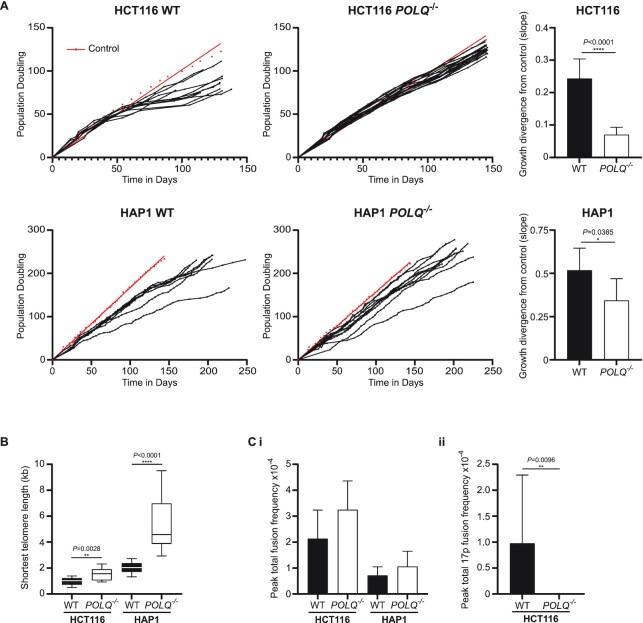
*POLQ* ^–/–^ clones are resistant to crisis-induced growth deceleration but not telomere fusions. (**A**) Growth curves for 11 HCT116 and 8 HAP1 WT *DN-hTERT*-transduced single cell clones are displayed (left panels) in comparison with 16 HCT116 and 10 HAP1 *POLQ*^–/–^*DN-hTERT*-transduced single cell clones (central panels). Growth trajectories of control clones of each lineage transduced with puromycin selection cassette alone are plotted in red for each grouping with curves fitted to a linear regression model. Linear regression best-fitting lines were generated for each individual clone and slope values subtracted from appropriate controls to estimate the degree of growth divergence (right panels) for WT (black bars) and *POLQ*^–/–^ clones (white bars). Mean slope differentials with 95% confidence interval (CI) are depicted and statistical significance was calculated using unpaired *t*-tests with Welch’s correction. (**B**) The shortest telomere lengths recorded for HCT116 (17p) and HAP1 (XpYp) WT (black) and *POLQ*^–/–^ (white) clones during crisis are presented as a box and whisker plot with statistical significance evaluated with Mann–Whitney unpaired *U*-tests. (**C**) (i) Total telomere fusion frequency (based on diploid genome DNA inputs into fusion PCR) for each WT and *POLQ*^–/–^ clone was calculated at each PD sampled. Maximal fusion frequencies for all WT (black bars) and *POLQ*^–/–^ (white bars) clones are displayed as means with 95% CI and statistical significance determined using Mann–Whitney unpaired *U*-tests. (ii) The differential 17p telomere intra-chromosomal fusion frequencies for HCT116 WT (black bars) and *POLQ*^–/–^ (white bars) are shown (the 17p subtelomere was not amplifiable in HAP1 clones). Mean values with 95% CI are displayed and Mann–Whitney unpaired *U*-tests were performed.

Following retroviral transduction with a dominant-negative *hTERT* expression cassette (*DN-hTERT*) and single cell cloning, the absence of POLQ was not detrimental to population growth (Figure 1A) during the phases of experimentally induced telomere erosion and telomere-driven crisis. All single cell clones except two HAP1 WT clones persisted through crisis, ultimately escaping through the reactivation of telomerase (Supplementary Figure S1Ci and ii) in order to maintain telomere lengths and evade replicative senescence. Thus, in contrast to related constituents of end-joining DNAR (51), POLQ is either not essential for escape from a telomere-driven crisis or detrimental effects are tempered by the longer telomere lengths engendered by its absence (Supplementary Figure S1B). Although POLQ expression has long been recognized to promote random integration events (67–69), we did not uncover any inequalities in *DN-hTERT* genomic assimilation among WT and POLQ clones (Supplementary Figure S1Ciii and iv) preceding the initiation of telomere-driven crisis. WT HCT116 and HAP1 clones expressing *DN-hTERT* displayed characteristic growth retardation during telomere-driven crisis (Figure 1A). In contrast, the proliferation of *POLQ*^–^^/^^–^ clones closely paralleled the control clones transduced with puromycin resistance cassette alone, transiting crisis more rapidly than the WT clones. The crisis profiles of individual HCT116 and HAP1 WT and *POLQ*^–^^/^^–^ clones are depicted in Supplementary Figure S1Di–iv for evaluation of telomere erosion and subsequent stabilization with escape from crisis. Calculating the deviation of growth trajectory for each singular clone from the controls exposed the patent incongruity between WT and *POLQ*^–^^/^^–^ clones (Figure 1A, right panels), demonstrating that the absence of POLQ significantly reduced the impact of telomere erosion on population doubling (PD) rates. Overall, the *POLQ*^–^^/^^–^ clones underwent significantly more telomere erosion (2.13-fold greater in HCT116 *POLQ*^–^^/^^–^ and 1.57-fold greater in HAP1 *POLQ*^–^^/^^–^) than WT clones prior to telomere length stabilization (Supplementary Figure S1E). The *POLQ*^–^^/^^–^ clones entered crisis with longer mean telomere lengths and sustained this differential at crisis nadir (Figure 1B). Collectively, these results indicate that POLQ function is integral to growth deceleration during the exacerbated genomic instability that accompanies telomere crisis, allowing *POLQ*^–^^/^^–^ clones to transit crisis more rapidly and with longer telomeres than their WT counterparts.

### POLQ promotes, but is not essential for sister chromatid intra-chromosomal fusions in cancer cell lines

Telomere attrition was accompanied by the emergence of telomere fusions that could be amplified using single or combinations of subtelomere-specific primers to distinguish intra-chromosomal (sister chromatid) from inter-chromosomal (compound) recombinations during crisis in both WT and *POLQ*^–^^/^^–^ clones. Maximal (highest incidence recorded at any given PD time point) fusion frequencies were not statistically significantly different in *POLQ*^–^^/^^–^ than WT HCT116 and HAP1 cells (Figure 1Ci). Exacerbated fusion frequencies overall in HCT116 (Supplementary Figure S1F) correlate with the shorter mean telomere lengths of HCT116 clones compared with HAP1 clones (Figure 1B). Curiously, we discovered a significant deficit of intra-chromosomal fusions amplified from the HCT116 *POLQ*^–^^/^^–^ clones using the 17p subtelomere primer alone (Figure 1Cii).

**Figure 2.**
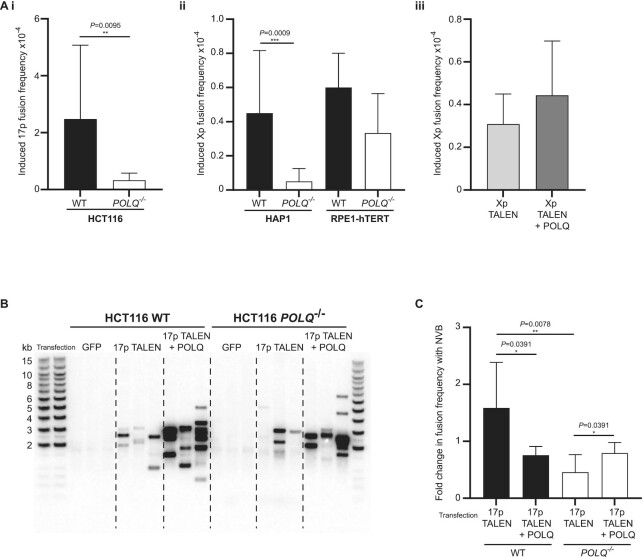
POLQ promotes but is not essential for sister chromatid telomere fusions in endonuclease-targeting experiments. (**A**) (i) 17p single primer intra-chromosomal fusion frequencies induced in HCT116 WT (black) and *POLQ*^–/–^ (white) cells nucleofected with 17p subtelomere-targeting TALEN pairs are presented as means with 95% CI of four experiments with Mann–Whitney unpaired *U*-tests applied. (ii) Total Xp intra-chromosomal telomere fusions detected in HAP1 or RPE1-hTERT WT (black) and *POLQ*^–/–^ (white) cells lipofected with TALEN pairs targeting the subtelomere and harvested at 48 h. Fusion frequency was calculated based on diploid genome DNA inputs into fusion PCR. Means with standard deviation (SD) derived from seven independent experiments are presented and statistical significance was determined using Mann–Whitney unpaired *U*-tests. (iii) A comparison of Xp intra-chromosomal fusion frequencies in all HAP1 and RPE1-hTERT cells transfected with Xp TALEN pairs alone (light grey) or concurrently with (dark grey) a POLQ expression vector. Mean values with 95% CI are displayed and Mann–Whitney unpaired *U*-tests were performed. (**B**) Southern blot using radiolabelled 17p subtelomere probe to detect 17p TALEN-induced 17p intra-chromosomal telomere fusions in WT and *POLQ*^–/–^ HCT116 samples co-transfected or not with a POLQ expression vector using lipofectamine. GFP transfections were performed as negative controls and to estimate transfection efficiencies in samples. (**C**) 17p intra-chromosomal fusion frequencies in HCT116 WT (black) and *POLQ*^–/–^ (white) cells lipofectamine transfected with 17p TALEN pairs in the presence or absence of a POLQ expression vector and 100 μM NVB for 48 h with dose replenished after 24 h. Data represent two biological and two technical replicates. Mean fold changes in fusion frequencies with 95% CI are displayed and significance was assessed using Wilcoxon matched-pairs signed rank tests.

To investigate this notable dearth of intra-chromosomal fusions in *POLQ*^–^^/^^–^ clones, we employed our previously established targeted nuclease-induced fusion assays (52,53) to capture different classes of telomere fusions within HCT116, HAP1 and RPE1-hTERT WT and *POLQ*^–^^/^^–^ parental cell lines (Figure 2 and Supplementary Figure S2). Transient transfection of HCT116 and HAP1 cells with a bespoke nuclease (TALEN) designed to elicit DSBs at a subtelomeric locus common to members of the 16p/21q families of homologous chromosome ends (38) resulted in robust induction of inter-chromosomal (dual chromosome) 16p and 21q telomere fusions synonymous to WT and *POLQ*^–^^/^^–^ cells (Supplementary Figure S2Ai). Cleavage of the unique 17p subtelomere by similar means was possible only for the HCT116 cell line, owing to the lack of a corresponding target site in HAP1 and RPE1-hTERT cells. In accordance with the HCT116 clones enduring telomere-driven crisis, 17p single chromosome (intra-chromosomal) fusions were abundant in TALEN-transfected WT cells (mean rate of 2.5 × 10^–4^/diploid genome), but rare (7.6-fold lower frequency, *P* = 0.0095) in *POLQ*^–^^/^^–^ cells (Figure 2Ai). Transfection efficiencies using two alternative methodologies (Supplementary Figure S2Aii and iii) were comparable for WT and *POLQ*^–^^/^^–^ HCT116 and RPE1-hTERT cells, precluding this as an explanation for the divergent 17p fusion yields.

To ensure that our discovery of suppressed intra-chromosomal fusions in *POLQ*^–^^/^^–^ cells was not specific to the 17p telomere or to HCT116 cells, we designed a novel TALEN to actuate DSBs exclusively within the XpYp subtelomere (Xp TALEN; Supplementary Figure S2Bi), permitting the original detection of Xp–Xp intra-chromosomal fusions in HAP1 and RPE1-hTERT cells that lack Y chromosomes (Figure 2Aii and Supplementary Figure S2Bii). Significantly, TALEN-induced Xp intra-chromosomal fusion frequencies were 9-fold (*P* = 0.0009) lower in HAP1 *POLQ*^–^^/^^–^ than WT cells and co-transfection with an exogenous POLQ expression vector increased the incidence of these fusions moderately (but not statistically significantly) in all cells (Figure 2Aiii). In essence, these data support a key role for POLQ in facilitating fusion between dysfunctional (eroded or TALEN-targeted) telomeres of sister chromatids, but not between telomeres of distinct chromosomes.

At telomeres, replication stalled by G4 quadruplex and R-loop secondary DNA structures may depend on POLQ function to rescue collapsed forks (70) and evade large-scale deletions (6); hence, loss of POLQ may conversely produce symmetric and extensively deleted sister chromatid fusions. Palindromic DNA sequences present a special challenge to DNA polymerases and genome stability (71,72), so we modified our telomere fusion assays to improve the capture efficiency of these molecules (Figure 2B and Supplementary Figure S2Ci). To minimize the burden of symmetrical PCR amplification, we employed two unidirectional primers aligning to separate regions of the 17p subtelomere for the investigation of TALEN-induced intra-chromosomal fusions in HCT116 cells. Using this strategy, 17p single chromosome fusions were more readily detected in *POLQ*^–^^/^^–^ cells (with the caveat that this technique may also facilitate amplification of fusions between replicated chromosomes) and with greater potency (3-fold, *P* = 0.0012 and 3.7-fold, *P* = 0.0023, upregulation in WT and *POLQ*^–^^/^^–^ cells, respectively) in cells co-transfected with the POLQ expression vector.

To probe further the contributions of POLQ to TALEN-induced intra-chromosomal fusions, we treated transfected HCT116 cells with the antibiotic POLQ ATPase inhibitor, NVB (25) (Figure 2C and Supplementary Figure S2Cii–iv). At the 100 μM dose found to selectively inhibit POLQ and repress end-joining repair by 50% (25), we determined no detrimental impact on transfection efficiency (Supplementary Figure S2Cii; not statistically significant), but a significant 1.5-fold reduction (*P* = 0.0313) in the viability of WT, but not *POLQ*^–^^/^^–^ cells that were evidently less vulnerable to POLQ regulation (Supplementary Figure S2Ciii). In contrast, NVB treatment subdued TALEN-induced intra-chromosomal fusion frequencies only in samples with atypical POLQ expression and not in WT non-supplemented cells (Figure 2C and Supplementary Figure S2Civ). These results suggest that POLQ expression and function may be insignificant in unstressed proliferating cell lines, but the consequences of aberrant or absent expression are effectively modulated by NVB either directly or indirectly (25,73) or through non-specific effects.

### POLQ-deficient cells escaping crisis manifest increased genomic heterogeneity

For improved resolution of POLQ-mediated DNAR during telomere-driven crisis and escape, we performed WGS of early and late crisis WT and *POLQ*^–^^/^^–^ HCT116 and HAP1 clones, as well as fusion amplicon sequencing of deep crisis samples (Supplementary Table S1). CNV profiles were generated by background subtraction of parental from early crisis and late from early crisis samples (48). Heatmaps illustrate the relative copy number (CN) changes across each cohort of HCT116 (Supplementary Figure S3A) and HAP1 (Supplementary Figure S3B) clones and CNV unique segments (Supplementary Table S2) are plotted as a karyotype map (Supplementary Figure S3Ci). CN gains at chromosomes 1q and Xp in the WT HAP1 parental line were not sustained in the crisis clones, resulting in losses reported at these locations for all HAP1 WT early crisis samples (Supplementary Figure S3B). The totality of CNVs for WT clones exceeded that of the *POLQ*^–^^/^^–^ clones of each cell line and was exacerbated by passage through crisis for most samples (Supplementary Table S2 and Supplementary Figure S3Cii). CN gains predominated in all lineages (Supplementary Figure S3Ciii) and were more abundant in WT than *POLQ*^–^^/^^–^ clones (Supplementary Figure S3Civ).

Unique crisis-induced deletions, inversions, duplications, translocations and insertions were next compared among WT and *POLQ*^–^^/^^–^ HCT116 (Supplementary Figure S4Ai) and HAP1 (Supplementary Figure S4Aii) clones. The overall frequencies of structural variants (SVs) were greater for HAP1 than HCT116 cells (Supplementary Table S3), but notably elevated among the WT samples of both lineages (Supplementary Figure S4Aiii). In late crisis samples, the mean total incidence of unique SVs was 11-fold (*P* = 0.00286) and 6.2-fold (*P* = 0.0171) higher in WT than *POLQ^–^^/^^–^* HCT116 and HAP1 samples, respectively (Supplementary Figure S4Aiv). There was no common paradigm for the types of SVs identified in WT compared with *POLQ*^–^^/^^–^ clones (Supplementary Figure S4Av), although HAP1 *POLQ*^–^^/^^–^ clones exhibited significantly fewer deletions and more numerous duplications than their WT counterparts. Superimposition of SV breakpoints with CNV distributions (Supplementary Figure S4B) reveals convergent events that may be symptomatic of the devastating genomic disruption that precedes stabilization of the cancer karyotype (46). To assess the contribution of telomere fusions to these susceptibility signatures, we measured intersections between unique SVs and genomic fusion junctions over increasing distance intervals. In both HCT116 and HAP1 WT clones, ∼20% all validated genomic telomere fusion junctions were located within 10 Mb of a unique SV, whereas far fewer fusions derived from HCT116 *POLQ*^–^^/^^–^ clones compared with HAP1 *POLQ*^–^^/^^–^ clones (7% compared with 17%) displayed analogous coincidence with unique SVs. Mapping these foci of crisis-induced DNA damage (Supplementary Table S4) uncovers genomic loci that may facilitate clonal escape, with particular spotlight on lesions prevailing across multiple samples.

CNV calls can be diluted by polyclonality, whereby genomic heterogeneity obscures recombinations that occur in individual clones. We performed clonality estimates based on single-nucleotide polymorphism unique variant allele frequencies (VAFs) to evaluate the extent of polyclonality in all HCT116 (Supplementary Figure S5A) and HAP1 (Supplementary Figure S5B) WT and *POLQ*^–^^/^^–^ clones. Clones for which the median VAF displayed a considerable shift below 0.5 (consistent with an allele-specific variant within a monoclonal population) were considered to be polyclonal following their transit through crisis. For both HCT116 and HAP1 cells, polyclonality was prevalent among the *POLQ*^–^^/^^–^ and not the WT clones (Supplementary Figure S5C). Collectively, 100% HCT116 and 75% HAP1 *POLQ*^–^^/^^–^ clones were considered polyclonal post-crisis in stark contrast to the WT clones of each line, of which only 25% were categorized as polyclonal. This potential subclonal diversity is consistent with both the augmented telomere fusion rates and the rapid crisis transit of *POLQ*^–^^/^^–^ clones that could have facilitated the emergence of multiple derivatives each bearing independent rearrangements promoting successful stabilization of telomere length. The co-existence of assorted subclones likely accounts for the reduced CNVs and SVs observed for the *POLQ*^–^^/^^–^ clones (Supplementary Figures S3 and S4), whereas idiosyncratic genomic rearrangements in WT clones largely converged on the emergence of single cell lines with homogeneous CNVs exceeding threshold detection. Thus, *POLQ*^–^^/^^–^ populations may be characterized by genetic heterogeneity arising from divergent evolution during telomere-driven crisis.

### *POLQ* ^–/–^ clones demonstrate altered telomere fusion profiles

**Figure 3.**
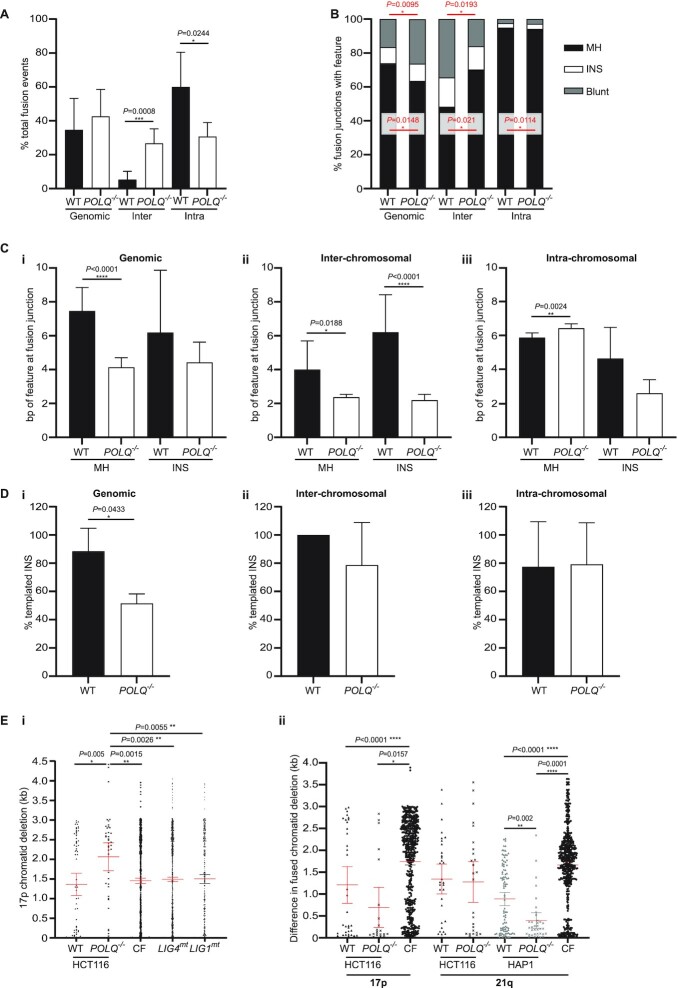
POLQ-deficient cells present elevated inter-chromosomal fusion incidence counterbalanced by reduced intra-chromosomal fusion frequencies. (**A**) The proportions of all sequence-validated telomere fusions derived from HCT116 and HAP1 WT (black) and *POLQ*^–/–^ (white) clones that can be classified as genomic, inter-chromosomal or intra-chromosomal events are presented as means with 95% CI. Statistical significance was evaluated using Mann–Whitney unpaired *U*-tests. (**B**) Stacked bar charts indicate the percentages of junctions within each telomere fusion subcategory that demonstrate microhomology (MH; black), insertions (INS; white) or are blunt-ended (blunt; grey). Data for WT and *POLQ*^–/–^ clones were compared using Fisher’s exact tests with significance indicated in red. (**C**) The mean numbers (with 95% CI) of nucleotides (bp) of microhomology or insertions at (i) genomic, (ii) inter-chromosomal and (iii) intra-chromosomal telomere fusions determined for WT (black) and *POLQ*^–/–^ (white) clones. Mann–Whitney unpaired *U*-tests were utilized to assess significance. (**D**) The proportions of all WT (black) and *POLQ*^–/–^ (white) (i) genomic, (ii) inter-chromosomal and (iii) intra-chromosomal telomere fusion junctions with sequence insertions that are locally templated are shown as mean values with SD. Significance was determined with Fisher’s exact tests. (**E**) (i) The amount of deletion (in kb) of each 17p chromatid involved in an intra-chromosomal telomere fusion amplified from HCT116 WT (filled triangles) and *POLQ*^–/–^ (crosses) clones juxtaposed with chromatid deletion data pertaining to crisis fibroblasts derived from patients with *LIG4* or *LIG1* mutations (mt; dash symbols) or normal controls (CF; filled circles) (66). Data are displayed as means with 95% CI in red and statistical significance was analysed using Mann–Whitney unpaired *U*-tests. (ii) The difference in deletion (kb) of fused 17p (left; HCT116 only) or 21q family chromatids in intra-chromosomal fusions amplified from WT (filled triangles) and *POLQ*^–/–^ (crosses) HCT116 (black) and HAP1 (grey) clones or crisis fibroblasts (CF; filled circles) is illustrated with means and 95% CI annotated in red. Mann–Whitney unpaired *U*-tests were used to compare datasets.

High-throughput sequencing of telomere fusion amplicons generated from WT and *POLQ*^–^^/^^–^ deep crisis samples enabled large-scale discrimination of distinct classes of telomere fusion (Figure 3 and Supplementary Figure S6). In accordance with our former observations (Figure 1Ci), fusion calls were 1.94-fold (*P* = 0.0328) higher in *POLQ*^–^^/^^–^ than WT clones (Supplementary Figure S6Ai). Intra-chromosomal fusions were defined as those between chromatids of a single chromosome (17p or the 16p or 21q telomere families), caveated by the cognition that homology between 16p and 21q telomere family members precludes definitive discrimination of true sister chromatid fusions. Inter-chromosomal fusions were construed as those comprised of distinct chromosome ends and genomic fusions were defined as recombinations between telomeres and distant loci throughout the genome. In agreement with our primary observations in crisis (Figure 1Cii) and nuclease-treated cells (Figure 2), we discovered a significant 2-fold reduction in the proportion of fusions that were intra-chromosomal for the *POLQ*^–^^/^^–^ compared with the WT clones (Figure 3A and Supplementary Figure S6Aii). Residual intra-chromosomal fusions sequenced from *POLQ*^–^^/^^–^ clones may represent confounding inter-chromosomal events; however, 17p (unique telomere) as well as 21q single chromosome fusions were detected in HCT116 *POLQ*^–^^/^^–^ samples, suggesting that these may represent genuine POLQ-independent intra-chromosomal events. A reversed skew was determined for inter-chromosomal fusion proportions that were ∼5-fold lower in the WT than the *POLQ*^–^^/^^–^ clones.

We next examined all authenticated fusion junctions for evidence of microhomology (MH) usage and insertion (INS) of extraneous sequence (Figure 3B–D). While MH usage dominated at intra-chromosomal fusions (Figure 3B), this was only minimally (but significantly) affected by the absence of POLQ. MH usage was also 1.2-fold (*P* = 0.0148) reduced at genomic junctions in *POLQ*^–^^/^^–^ clones, with a concomitant 1.6-fold (*P* = 0.0095) rise in the proportions of blunt fusions lacking MH or INS. Inter-chromosomal telomere fusions presented inverted proportions in comparison, with MH usage increasing 1.5-fold (*P* = 0.021), accompanied by a >2-fold (*P* = 0.0193) contraction of blunt fusion fractions in *POLQ*^–^^/^^–^ clones. The proportions of fusion junctions bearing INS were relatively stable for all fusion categories. MH and INS lengths (Figure 3Ci–iii) were customarily curtailed by the loss of POLQ, except at intra-chromosomal junctions, where the mean magnitude of MH rose by 0.5 bp (Figure 3Ciii) in the *POLQ*^–^^/^^–^ clones. These expositions suggest a transformation in DSB processing in the absence of POLQ, with shorter stretches of MH or inserted DNA required to stabilize junctions. INS templated by local sequence content predominated over random INS at all junctions (Figure 3Di–iii) but were notably (1.7-fold, *P* = 0.0433) depleted at genomic fusions amplified from *POLQ*^–^^/^^–^ clones despite the considerable mean INS length being preserved (Figure 3Ci). A degree of decoupling of fusion processing from local sequence context can therefore be inferred for the *POLQ*^–^^/^^–^ clones.

We ascertained variations in the proportions of intra-chromosomal fusions contributed by the distinct chromosome ends among WT and *POLQ*^–^^/^^–^ clones (Supplementary Figure S6B). Although impracticable to resolve, the altered balance between 17p and 21q events in HCT116 (Supplementary Figure S6Bi) and between 21q and 16p events in HAP1 (Supplementary Figure S6Bii) could indicate a substitution of some authentic intra-chromosomal 17p (HCT116) and 21q (HAP1) fusions for more ambiguous inter-chromosomal 21q (HCT116) and 16p (HAP1) events in the *POLQ*^–^^/^^–^ clones. We next appraised the deletion of individual chromatids participating in 17p–17p (HCT116; Figure 3Ei) or 21q–21q (HCT116 and HAP1; Supplementary Figure S6C) telomere fusions and calculated the difference in deletion at fused pairs revealed through sequencing as a measure of the intramolecular symmetry (Figure 3Eii). In comparison with WT, as well as our previously acquired (66) datasets pertaining to crisis fibroblasts derived from patients with DNA ligase 1 (*LIG1*) or ligase 4 (*LIG4*) mutations and controls, *POLQ*^–^^/^^–^ HCT116 clones evinced significantly greater deletion of 17p chromatid constituents of intra-chromosomal fusions (Figure 3Ei). Corresponding analyses at 21q (Supplementary Figure S6C) were confounded by the uncertainty inherent to intra- versus inter-chromosomal fusion discrimination; however, the mean differences in deletion at fused chromatids were lower for HCT116 *POLQ*^–^^/^^–^ clones at 17p and 21q (although not statistically significant) and HAP1 *POLQ*^–^^/^^–^ clones at 21q (*P* = 0.002; Figure 3Eii). These results suggest a reduction in asymmetry of intra-chromosomal fusions in *POLQ*^–^^/^^–^ clones that can be explained, at least in part, by exaggerated deletion of chromatid pairs.

### *POLQ* ^–/–^ clones reveal diminished incidence of telomere fusions with genes and repetitive DNA

**Figure 4.**
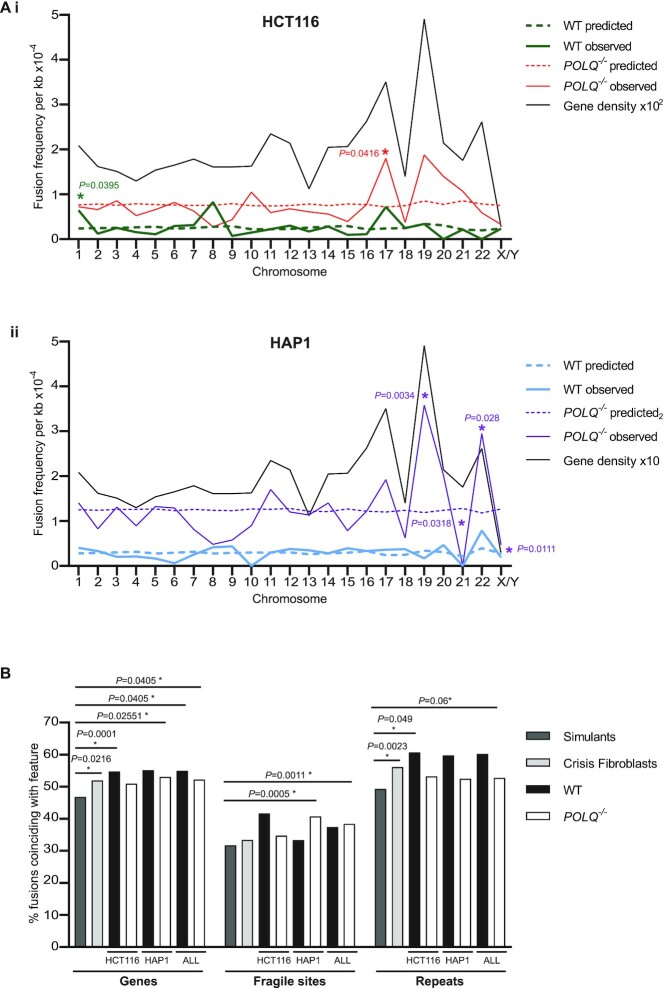
Reduced incidence of telomere fusions with genes and repeats amplified from *POLQ*^–/–^ clones. (**A**) The frequencies of all validated genomic telomere fusions amplified from (i) HCT116 and (ii) HAP1 crisis clones localizing to individual chromosomes are presented normalized to chromosome sizes (straight lines; WT in bold). Predicted frequencies based on mean genomic telomere fusion frequency per sample are displayed as dashed lines and significant discrepancies determined by Fisher’s exact tests marked with an asterisk. The overall gene density for each chromosome is also featured as a straight black line in each plot. (**B**) The proportions of genomic telomere fusions derived from WT (black) and *POLQ*^–/–^ (white) HCT116 and HAP1 clones or control crisis fibroblasts (light grey) or a simulated fusion dataset (mid-grey) whose junctions coincide with genes, fragile sites or repeat sequences throughout the genome are shown. Significance was tested using Fisher’s exact tests, except where the substantial simulant dataset was compared and chi-squared tests with Yates’ correction were employed instead.

To examine potential consequences of divergent genomic fusion processes, we plotted each manually validated genomic telomere fusion junction as a karyotype (Supplementary Figure S7A). Adjusting chromosome fusion frequencies to chromosome size facilitated the distinction of individual chromosomes bearing fusions surpassing those predicted from mean sample fusion rates (Figure 4A). From these charts, it was possible to discern elevated fusion frequencies for HCT116 (Figure 4Ai) and HAP1 (Figure 4Aii) WT clones at chromosome 8 that could not be attributed to chromosome size or mean gene density. This observation correlates with our former disclosure that chromosome 8 harbours a surfeit of genes differentially expressed in crisis fibroblasts disproportionate to its size and gene density (66). There were no overlaps in chromosomes equivalently independently enriched in fusions from *POLQ*^–^^/^^–^ clones and the prevalence of fusion junctions at chromosomes 11, 17, 19 and 22 was considered interconnected with gene density along these chromosomes.

Assessment of fusion junction coincidence with key determinants of DNA structure and accessibility (Figure 4B) was conducted in juxtaposition with crisis fibroblast and simulated fusion datasets (66) to uncover significant variation. Although a consistent and robust trend for reduced association of fusion junctions from *POLQ*^–^^/^^–^ clones with repetitive DNA was evident, this did not reach statistical significance and the overarching discovery was of the discrepancy between the simulated dataset and both cancer cell lines. Consequently, we proceeded to evaluate fusion coincidence with fragile sites and DNA repeats defined by RepeatMasker [Smit, A.F.A, Hubley, R. and Green, P. (2013–2015) RepeatMasker Open-4.0; http://www.repeatmasker.org) in comparison with mean genome density using BEDTools (74). These analyses indicated significant concurrence of WT but not *POLQ^–/–^* fusion junctions with DNA repeats (HCT116 WT, *P* = 6.05 × 10^–3^; HAP1 WT, *P* = 0.0315; data not shown). No such incongruity was resolved at fragile sites. Similarly, there was no coherent difference in the partition of fusion junctions within genes among exons and introns (Supplementary Figure S7B) and the association of fusions derived from *POLQ*^–^^/^^–^ clones with genes of shorter mean lengths was not significant (Supplementary Figure S7Ci and ii). Tangentially, we found an absence of intersection of genomic fusion junctions from all samples with 124 L1 LINE loci recently identified as drivers of oncogenic rearrangements (Supplementary Figure S7D) (75). Collectively, these observations suggest a special contribution of POLQ to recombinations between dysfunctional telomeres and repetitive loci across the genome.

### Telomere fusions occur within accessible and unstable genomic loci

Fused genes identified in HCT116 and HAP1 crisis clones were investigated using the Broad Institute Gene Set Enrichment Analysis (GSEA) Molecular Signatures Database (76) (MSigDB database v7.4; Supplementary Tables S5 and S6). Genes fused in WT HCT116 and HAP1 clones were enriched in neurological and microRNA gene networks that may portray the crisis transcriptome (77–79) since fusion with longer genes (80,81) (Supplementary Figure S7C) and a particular vulnerability of chromosome 8 (82) was identified for these samples (Figure 4A). Concurring with our prior conjecture that damaged telomeres are prone to fusion with actively expressed genes during crisis (37,66), over-represented networks within *POLQ*^–^^/^^–^ genomic fusions included HMGB1 target genes, connecting the DNAR engaged during this period of genomic vulnerability (83) with propagation of instability. STRING (84) functional protein interaction analyses complemented the GSEA results (Supplementary Figure S8A and B). The extent of network interactions in the *POLQ*^–^^/^^–^ clones significantly exceeded predicted probability (*P* = 0.000186; Supplementary Figure S8C and Supplementary Table S7), affirming a relatedness among genes incorporated into telomere fusions during crisis that is likely underpinned by common regulation or expression, as well as localization (85).

### *POLQ* ^–/–^ clones exhibit a propensity for telomere interactions with centromeric alpha-satellite repeats

**Figure 5.**
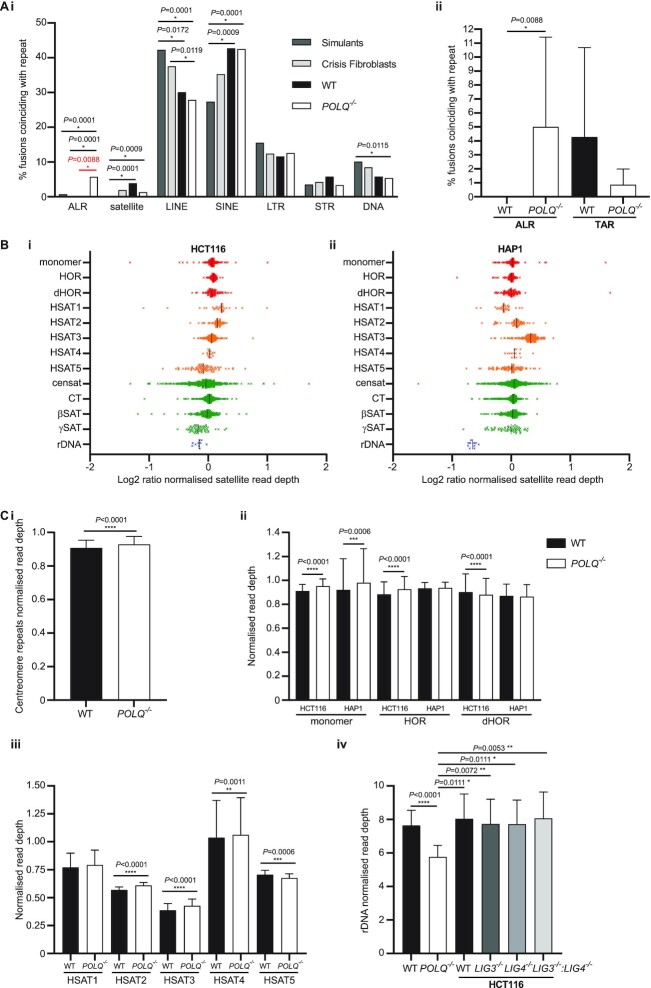
Increased frequency of *POLQ*^–/–^-derived telomere fusions with ALRs. (**A**) (i) The proportions of genomic telomere fusions derived from WT (black) and *POLQ*^–/–^ (white) HCT116 and HAP1 clones or control crisis fibroblasts (light grey) or a simulated fusion dataset (mid-grey) whose junctions coincide with repeat features characterized by RepeatMasker are shown. ALR, alpha-satellite; satellite, other satellite sequences; LINE, long interspersed nuclear elements; SINE, short interspersed nuclear elements; LTR, long terminal repeats; STR, short tandem repeats; DNA, DNA transposons. Significance was tested using Fisher’s exact tests, except for comparisons using the simulant dataset where chi-squared tests with Yates’ correction were required. (ii) The proportions of genomic telomere fusions derived from WT (black) and *POLQ*^–/–^ (white) HCT116 and HAP1 clones whose junctions coincide with ALRs or TARs are displayed as means with 95% CI with significance evaluated using Fisher’s exact tests. (**B**) Assessment of satellite read depth from WGS data for each (i) HCT116 and (ii) HAP1 parental cell line as a proxy for satellite genomic content. Satellite regions from the T2T reference v1.0 (Nurk, S. *et al.* bioRxiv 2021.2005.2026.445798) were extracted as a new mini reference to which parental line WGS data were aligned. Read depths were determined using Mosdepth (149) and normalized to the mean of the sample. Log_2_ ratios of *POLQ*^–/–^ compared with WT read depths are displayed for various satellite motifs (Gershman, A. *et al.* bioRxiv 2021.2005.2026.443420). Monomer, alpha-satellite monomeric sequence; HOR, higher order repeat; dHOR, divergent HOR; HSAT, human satellite sequence; censat, centromere satellite repeats; CT, centric transition regions; βSAT, beta-satellite repeats; γSAT, gamma-satellite repeats; rDNA, ribosomal DNA. (**C**) Normalized mean read depths relating to centromere-associated repeats (monomer, HOR and dHOR sequences) are (i) compared for all WT (black) and all *POLQ*^–/–^ (white) clones or (ii) separated by repeat element and cell line. Statistical significance was determined using Wilcoxon matched-pairs signed rank tests. (iii) Normalized mean read depths for HSAT1–5 repeats for WT (black) and *POLQ*^–/–^ (white) parental lines with 95% CI. Statistical significance was evaluated using Wilcoxon matched-pairs signed rank tests. (iv) Normalized mean read depths relating to rDNA repeats for WT (black) and *POLQ^–/–^* (white) alongside WT (black) and DNA ligase-deficient (grey tones) HCT116 parental lines with 95% CI. Wilcoxon matched-pairs signed rank tests were employed to compare WT and *POLQ^–/–^* samples and Mann–Whitney unpaired *U*-tests were used to test the statistical significance of observed differences between these and the DNA ligase-deficient lines.

In exploring the coincidence of genomic fusion junctions with DNA repeats, we perceived an unusual accretion of telomere fusions with alpha-satellite repeats (ALRs) in *POLQ*^–^^/^^–^ compared with WT clones (*POLQ*^–^^/^^–^ clones 17/294 genomic fusions with repeats compared with 0/103 WT genomic fusions with repeats; Figure 5Ai and ii). Telomere recombinations with ALRs were rarely recorded in our crisis fibroblast and simulated datasets but were variably detected in DNAR mutant models (Supplementary Figure S9A) and significantly enriched (*P* = 0.0088) in *POLQ*^–^^/^^–^ clones (Figure 5A and Supplementary Figure S9A). Comparisons with alternative specialized DNA repeat features (other satellites, LINE, SINE, LTR, STR and DNA repeats) revealed statistically significant differences between simulants or crisis fibroblast controls and the cancer cell lines, but no additional differentials between WT and *POLQ*^–^^/^^–^ clones, including at telomere-associated repeats (TARs; Figure 5Aii). Genome-wide evaluation of this exceptional relationship with ALRs confirmed statistical significance (*P* = 0.0139) for the concurrence of *POLQ*^–^^/^^–^ HCT116 (but not HCT116 WT or HAP1) fusion junctions with higher order tandem repeats (HORs) of alpha-satellite units that comprise human centromeres. Hence, the absence of POLQ permits long-range telomere–centromere interactions akin to those implicated in cancer neochromosome formation (46,86).

If the mechanisms regulating telomere elongation in POLQ-deficient cells (Supplementary Figure S1) are pertinent also to centromeric repeats (87), then repetitive sequence could conceivably occupy a larger proportion of the genomic DNA content of *POLQ^–^^/^^–^* than WT clones, raising the probability of DSBs and fusions occurring within these tracts. We performed an assessment of satellite read depth as a proxy for genomic satellite repeat content in WT and *POLQ^–^^/^^–^* parental lines in order to test our hypothesis of ALR expansion in the absence of POLQ. Parental HCT116 lines with compromised DNAR components were analysed in parallel for standardization. Centromere sequences are insufficiently resolved and curated in the GRCh38 human genome assembly, so alignments were conducted using satellite regions (Gershman, A. *et al.* bioRxiv 2021.2005.2026.443420) extracted from the Telomere-to-Telomere Consortium (T2T) reference v1.0 (Nurk, S. *et al.* bioRxiv 2021.2005.2026.445798) as a subsidiary reference. Read depths pertaining to 13 distinct satellite features [determined using Mosdepth (149)] were normalized to the mean of each sample and are presented as log_2_ ratios for *POLQ^–^^/^^–^* compared with WT HCT116 (Figure 5Bi) and HAP1 (Figure 5Bii) cells. Contrary to prediction, higher read depths for ALR monomers and HOR sequences were not consistently determined for *POLQ^–^^/^^–^* cells (Figure 5Ci and ii). Moderate, but significant, increases in reads specifically aligning to human satellites (HSAT) 2, 3 and 4 that form large tandem repeat arrays adjacent to centromere regions (88) were, however, discovered for *POLQ^–^^/^^–^* cells (Figure 5Ciii and Supplementary Figure S9Bi). These arrays are heterogeneous in size and content amongst individuals (89), representing biomarkers of unequal crossover and sources of human variation (90). Despite the inflated read coverage for *POLQ^–^^/^^–^* samples, there was no remarkable coincidence of telomere fusions with HSAT sequences (88) transposed into the GRCh38 genome reference. Taken together, these results revoke the supposition that the amplification of satellite repeat units accounts for the elevated frequencies of telomere fusions with these sequences in the absence of POLQ.

We also detected divergence at HSAT2 and HSAT3 sequences among HCT116 DNAR-deficient lines (Supplementary Figure S9Bii), with cells compromised in both classical (LIG4-dependent) and alternative (LIG3-dependent) NHEJ repair (*LIG3*^–^^/^^–^:*LIG4*^–^^/^^–^) exhibiting significantly altered read depths compared with WT and *LIG3^–^^/^^–^* or *LIG4^–^^/^^–^* single mutants. The direction of change was opposite for the repeat classes, however, signifying that disparate or bifunctional mechanisms may be in operation in cells with defective DNAR. We additionally identified robust decrements in yields of reads aligning to rDNA repeat sequences in the *POLQ^–^^/^^–^* cells compared with WT and HCT116 DNA ligase-deficient models (Figure 5Civ and Supplementary Figure S9Biii), marking the impact of POLQ deficiency as distinct from that of other components integral to end-joining repair. POLQ, therefore, appears to govern the stability of specific DNA repeat classes. Long tandem rDNA repeats undergo expansion and contraction mediated by gene conversion and unequal crossover (91) in response to replication stress and mutation (92,93). These data implicate POLQ in processes including replication-dependent recombination that contribute to genomic fluidity in homeostatic and cancerous states (87,94,95).

## DISCUSSION

Through single cell cloning of *POLQ^–^^/^^–^* HCT116 and HAP1 HR-proficient cells, we have been able to assemble coherent insights into the specific functional contributions of POLQ to DNAR and recombination during episodes of prolonged cellular stress. While not essential for clonal escape from a telomere-driven crisis, POLQ activity evidently shapes the developing cancer genome, stimulating distinctive molecular interactions with identifiable sequelae that may be of value in the diagnosis or targeted treatment of cancer patients.

### *POLQ* ^–/–^ clones experience accelerated transit through telomere-driven crisis

*POLQ^–^^/^^–^* clones derived from both HCT116 and HAP1 cancer cell lines presented telomere erosion and fusion rates comparable with WT cells, yet population growth deceleration during crisis was minimal (Figure 1). This peculiarity of *POLQ^–^^/^^–^* cells likely results from the summation of multiple outcomes of pathways imbalanced by the absence of POLQ (85), including the stabilizing impact of elongated telomeres (Figure 1B). POLQ expression impacts replication timing (96,97), with cells that overexpress POLQ experiencing replication delays. The converse may be true for *POLQ^–^^/^^–^* clones undergoing a telomere-driven crisis, so that cell cycle progression and population doubling rates may be expedited, with reduced opportunity for corrective DNAR that safeguards genomic integrity. As POLQ plays an important role in the rescue of intermediates of aborted HR that materialize late in the cell cycle (22), frustration of TMEJ may also deter cell cycle pausing pre-mitosis, so that chromosomal damage persists (98,99), is extruded into micronuclei (MN) (100) or triggers apoptosis without suspension of proliferation. In concert with the skewed distributions of inter- and intra-chromosomal telomere fusions (Figure 3A), POLQ deficiency supports long-range DNA recombinations that precipitate genomic heterogeneity (100), manifest in the polyclonal post-crisis populations (Supplementary Figure S5). Chromosome segregation defects may result in ploidy increments in POLQ-deficient cells that contribute to subclonal diversification (101) through juxtaposition and amplification of extra-chromosomal DNA, including ALRs and co-regulated genes (102,103). Greater genetic variability in the absence of TMEJ (85) generates the potential for transcriptional and phenotypic diversity (Supplementary Figures S7 and S8) that promotes successful clonal evolution (97,104) and telomere length stabilization. The intersection of telomere fusions with expressed genes, as well as structural and CN aberrations (Figure 4 and Supplementary Figures S3 and S4), reveals the convergence of destabilizing events that propagate genomic volatility and sponsor malignant transformation (46,105). Thus, POLQ deficiency removes the possibility of HR salvage and facets of ANHEJ that might ordinarily detain cell cycle progression, resulting in accelerated growth of genetically diverse populations accompanied by vigorous attrition of cells bearing lethal inter-chromosomal recombinations.

### *POLQ* ^–/–^ clones exhibit altered distributions of telomere fusion classes

Diminished intra-chromosomal contrasting with heightened inter-chromosomal telomere fusion frequencies were recorded for POLQ-deficient cells in both telomere-driven crisis (Figures 1 and 3) and endonuclease-mediated subtelomere-targeting experiments (Figure 2). Overexpression of exogenous POLQ correspondingly enhanced yields of intra-chromosomal 17p and Xp telomere fusions (Figure 2), decidedly implicating POLQ in this specific post-replicative repair (52,53,106). We have previously established the discrete operation of CNHEJ and ANHEJ in the prosecution of inter- or intra-chromosomal telomere fusions, respectively (51,52). POLQ is an established protagonist in ANHEJ (12,20) that predominates in recombinations between the telomeres of sister chromatids (30,38). With compromised TMEJ capability, eroded and damaged telomeres are captured within inter-chromosomal fusions, shifting the overall balance of repair events from localized ANHEJ-mediated intra-chromosomal rearrangements (21) to long-range CNHEJ-mediated inter-chromosomal interactions (52). The moderate increases in genomic fusion frequencies we observed in *POLQ*^–/–^ clones (Figure 3), as well as the ultimate expansions in genomic heterogeneity (Figure 5), further illustrate this transformation in repair pathway utilization. Our results, however, demonstrate that POLQ is not invariably essential for intra-chromosomal telomere fusions since these events can still be amplified from POLQ-deficient cells (Figures 2 and 3). Furthermore, the capacity for NVB (25) to reduce intra-chromosomal fusion frequencies in cells with both surplus and deficiencies in POLQ (Supplementary Figure S2Civ) implicates both POLQ-dependent and POLQ-independent pathways (107). This conclusion is consistent with the finding that genetic abrogation of POLQ does not prevent escape from telomere-driven crisis (Figure 1), in contrast with LIG3-deficient cells that cannot expedite sufficient non-lethal recombinations to stabilize telomere length and support cell viability (51). These observations are also compatible with two recent reports (108,109) demonstrating that only ∼50% of the MH generated at a repair junction is likely POLQ-dependent.

An additional layer of complexity is provided by the impact of POLQ deficiency on replication timing and cell cycle progression (96,97) that may constrain opportunities for HR, resulting in greater cellular dependence on NHEJ. Compounded by the diminished facility for repair at replication forks stalled by telomeric secondary structures (70), recovery of interrupted HR products in *POLQ*^–/–^ cells is likely significantly impaired, so that fewer sister chromatid fusions are captured and perpetuated in proliferating cultures (66). The altered proportions of telomere fusion classes in *POLQ*^–/–^ compared with WT cells may also reflect a lower efficiency of replication of palindromic molecules that are intrinsic to intra- but not inter-chromosomal telomere fusions (52,53). Coherent with research identifying large MH-agnostic deletions in *POLQ*^–/–^ cells (14,18,94), we observed exacerbated deletion of both chromatids contributing to intra-chromosomal fusions (Figure 3E) in *POLQ*^–/–^ clones, leading to an overall loss of asymmetry that could encumber replicative polymerases (71,72). Thus, the reduction in intra-chromosomal fusions in POLQ-deficient clones could result from the under-representation of ANHEJ-mediated repair in combination with the sacrifice of particular repair outputs.

### POLQ-mediated telomere fusions display characteristic junction features

Large-scale sequence analyses of telomere fusions amplified from 8 HCT116 and 10 HAP1 clones that had endured and escaped from a telomere-driven crisis permitted confident resolution of remarkable differences in the processing of telomere recombinations by WT and *POLQ*^–/–^ cells (Figure 3). In the absence of POLQ, we detected significant decrements in the proportions of genomic and intra-chromosomal fusions bearing junctional MH, with corresponding elevations in the percentages of blunt junctions. These observations are consistent with supplementary CNHEJ-mediated repair compensating for the insufficiency of POLQ-dependent ANHEJ (12). An alternative prospect is that, in the absence of POLQ, substituted repair processes (110) exploit MH more distant from the ultimate fusion junction to stabilize the ligation and these may have been omitted from our analyses. In contrast, we determined enhanced MH usage at the inter-chromosomal telomere fusions that are also more abundant in *POLQ*^–/–^ clones. While appearing incongruous with the supposition that CNHEJ is the prevailing mode of repair at these junctions (111,112), the actual lengths of MH employed at inter-chromosomal fusion junctions were lesser than those at genomic and intra-chromosomal fusion junctions and were further tempered by POLQ deficiency. The discrepancies in MH lengths at POLQ-independent inter-chromosomal compared with genomic and intra-chromosomal fusions (means of 2.37, 4.14 and 6.44 bp, respectively) suggest distinct processing and end-ligation mechanisms propel inter-chromosomal rearrangements, while refuting the likelihood of diversion into repair processes dependent on more extensive MH, such as single-strand annealing (107,110,113,114).

Efficient TMEJ has been reported to require at least 3 bp of end-proximal MH (94), with shorter sequences dependent on multiple cycles of annealing and DNA synthesis that scar the repair locus (7,13,115). We did measure increased incidence of INS characteristic of iterative TMEJ (all of which were locally templated in WT samples) present at inter-chromosomal compared with genomic and intra-chromosomal fusions; however, these were not significantly depleted from *POLQ*^–/–^ clones. These results hence more credibly support the abounding contribution of CNHEJ to inter-chromosomal fusions in both the presence and absence of POLQ (116). MH and INS lengths were commonly reduced at all fusion junctions amplified from *POLQ*^–/–^ clones except for intra-chromosomal fusions, where MH sequences were moderately longer. These evaluations highlight the mutagenic essence of TMEJ as well as the inefficiency of some POLQ catalytic functions that require more extensive MH stretches and produce more marked genomic aberrations (3,5,7,11,117).

### POLQ suppresses telomere–centromere interactions

A salient discovery unearthed by our high-resolution examinations of telomere fusions with genomic loci was that of the significant enrichment of junctions within centromere ALRs detected in both HCT116 and HAP1 *POLQ^–^^/^^–^* clones (Figure 5 and Supplementary Figure S9). In the context of reduced overall frequency of telomere fusion with repetitive DNA determined for *POLQ^–^^/^^–^* clones, we initially contemplated whether centromere territories were unusually expanded in *POLQ^–^^/^^–^* clones in tandem with the extended telomere lengths we measured for these cells (Supplementary Figure S1). We rejected the basic probabilistic inference that elevated fusion incidence correlated directly with centromere size upon recording no consistent POLQ-dependent differences in sequence coverage of ALR monomers or HORs. In exploring this conundrum, we identified notable variations in human satellite (HSAT2, HSAT3 and HSAT4) and rDNA repeats common to *POLQ^–^^/^^–^*, but not other HCT116 DNAR-deficient models, proposing hitherto unrecognized functions of POLQ in satellite regulation.

Distended telomere and HSAT DNA repeats undoubtedly pose a greater replicative and energetic burden for the crisis-transiting cell. Longer repeat tracts may elicit replication stress through polymerase slippage (118) and fork stalling at secondary DNA structures (93). POLQ is recruited to centromere DSBs that occur in G1 cell cycle phase, along with HR components that ordinarily serve to restrict mutagenic translocations (119). The repressive histone methylation marks at heterochromatic regions including centromeres favour TMEJ-mediated DNAR, although POLQ processivity is inefficient at AT-rich sequences that comprise many human satellite classes (94,120,121), resulting in recurrent insertion scars (7) that evidence repeated rounds of aborted annealing and DNA synthesis. Through diverse interactions with HR, POLQ has been implicated in the rescue of both late-stage repair intermediates (20,122) and inter-homologue and non-allelic HR products (123,124), suppressing loss of heterozygosity (87). The exacerbated DSB-induced mitotic crossovers in POLQ-deficient cells could predicate the exaggerated sequence flexibility at satellite and rDNA repeats, as well as the telomere elongation detected in comparison with WT cells (91,92,125). The absence of POLQ at damaged satellites prevents both TMEJ-mediated translocations and salvage of failed HR, releasing these repetitive regions into alternative repair pathways that may promote the recombinations that underpin fluctuations in length.

Extended telomere lengths achieved in cloned cell lines could be artefacts symptomatic of the survival advantage conferred by the appended genetic buffer. The exclusivity of this finding to *POLQ^–^^/^^–^* cells of three independent lineages (HCT116, HAP1 and RPE1-hTERT) and converse observations in other DNAR models (Supplementary Figure S1) repudiate this notion, however. Furthermore, we determined no significant differences in *DN-hTERT* integration between WT and *POLQ^–^^/^^–^* clones despite demonstrations that TMEJ is responsible for the majority of random integrations of exogenous DNA into the genome (68,69,126). As suppression and reactivation of telomerase (Supplementary Figure S1C), as well as telomere erosion rates in crisis, were commensurate for WT and *POLQ^–^^/^^–^* clones (even though the shortest lengths achieved were not), our data suggest a novel and unique capacity of POLQ for modulating repetitive sequences.

Mechanistically, POLQ may influence telomere length through various non-exclusive means, including altered accessibility of telomerase to the telomeric 3′ overhang for catalysis of repeat synthesis. A role for the shelterin component, hPOT1, in telomere protection and length regulation has recently been elucidated (127). *POT1* mutations in cancer patients that support telomere length extensions through impaired recruitment of telomerase to the telomere have been characterized (128). The agency of POLQ to cooperate in or regulate related responses, potentially through competition with ssDNA-binding RPA (21,107,129), could result in a comparable extended telomere phenotype when POLQ is abrogated. Akin to the diversified repair at satellite sequences in TMEJ-deficient cells, impaired ANHEJ at telomeres may sustain repair by replicative and recombinatorial processes that permit sequence length evolution in *POLQ^–/–^* cells. Recently, a novel role for the MutSα MMR protein in telomere maintenance has been recognized (130) whereby disruption of this function produces a hyper-recombination phenotype at telomeres. Although we detected no consistent evidence of alternative lengthening of telomeres (ALT) active in *POLQ*^–/–^ clones, the collaboration of POLQ with Holliday junction resolvases (87,95,122) advocates the potential for elongation resulting from a failure of heteroduplex rejection (131). In the absence of POLQ, recombinations between homeologous DNA (including telomeres and satellites) may not be successfully dissolved, leading to repeat tract extensions through premature initiation of DNA synthesis (130). Disruption of the balance of repair mechanisms at telomeres and satellites is credibly more consequential during the prolonged stress of telomere-driven crisis when the losses of POLQ contributions to ANHEJ (12,21), break-induced replication (132,133), BER (16) and MMR (17) coalesce to deliver numerous inter-chromosomal telomere fusions and recombinations with satellite repeats in *POLQ*^–/–^ clones.

Telomeres and rDNA repeats are intriguingly interconnected, sharing features of length regulation inclusive of a critical role for BLM helicase at both sequence classes (134). BLM syndrome cells in culture manifest increased variability at telomeres and reduced rDNA content compared with cells from healthy donors, so that defective helicase activity ostensibly perturbs repeat regulation at widespread sites. ALT cancers in which *ATRX* is mutated also retain lower rDNA content than ALT-negative cancers owing to defective histone H3.3 deposition and heterochromatin formation at rDNA repeats (135). In yeast and plant models, telomere and rDNA stability are symbiotic and rDNA repeats can be copied to chromosome termini to secure genome integrity (136,137). Although such rare rDNA translocations have not been identified in humans, the co-localization of regulatory elements underlines the potential for coordinated recombination (138). In HCT116 and HAP1 *POLQ*^–/–^ cell lines, we determined significant reductions in rDNA sequence coverage compared with WT cells and with other categories of satellite repeats (Figure 5). Contraction of these repeats in *POLQ*^–/–^ cells may result from elevated replication stress (93) without POLQ-mediated recovery at replication forks (139) and the corresponding variation at HSAT and telomere sequences indicates extensive influence of this polymerase over repeat stability (140). The detriment of POLQ helicase and end-joining activity (21,141,142) at rDNA as well as satellite and telomere repeats, coupled with potential displacement into MN (85), may precipitate the global genomic disequilibrium that we and others have observed in POLQ-deficient models (85,87,143).

Enhanced rates of fusion between telomeres and satellite repeats amplified from *POLQ^–^^/^^–^* cells evidence the combined impact of suppressed intra-chromosomal interactions counterbalanced by CNHEJ and the unproductive endeavours of replicative repair. The endonuclease capability of POLQ has been reported to preferentially cleave hairpin structures arising from intramolecular annealing, precluding DNA synthesis primed from these structures and, instead, promoting intermolecular annealing and end-joining (144). In POLQ-deficient cells, satellite repeats conceivably stabilize intermolecular contacts between homeologous sequences licensing aberrant repair. Long-range associations are facilitated by the increased mobility of DNA bearing DSBs, particularly in the G1 phase of cell cycle, when CNHEJ, but not HR repair, is operational (145). ANHEJ debilitated by *POLQ* disruption is surpassed by the efficiency and competency of CNHEJ that ligates distant DSB to conserve chromosome integrity. Thus, increases in long-range telomere–satellite recombinations in the absence of POLQ are products of the shift in DNAR capacity (110) coupled with variability at repeat tracts that amplifies the vulnerability of these loci.

### Clinical inhibition of POLQ inhibition may not be a universally effective strategy

Suppression of TMEJ using inhibitors such as NVB is expected to alter DNAR equilibrium, with exceptional impact in cancer cells harbouring existing repair defects, such as BRCA-deficient tumours (20). Enhanced long-range chromosomal interactions and compromised replication fork rescue could significantly affect cellular viability, potentially improving chemotherapeutic outputs and efficiencies (24,146). Lower doses of chemotherapeutics would limit undesirable side effects and peripheral damage to healthy tissue, encouraging patient compliance and wider socio-economic benefits. Augmented genetic and transcriptional heterogeneity consequential of exacerbated inter-chromosomal recombinations may also reinforce anti-tumoural immunity, generating cancer cell-derived neoantigens (147,148) that activate tumour cell killing, as well as establishing longer term immune surveillance of residual disease. Nonetheless, our results indicate that NVB may have additional POLQ-independent effects (Figure 2C) that will need to be considered for inclusion of this antibiotic in cancer treatment regimes. Laterally, we have uncovered DNAR fractions that are differentially contingent on POLQ function (Figures 2 and 3), so that repressing TMEJ may empower formerly restrained repair with unanticipated or negligible effect. Furthermore, the heightened genome instability that POLQ suppression may unleash (85,143) also has the potential to promote variation and clonal evolution with the possibility of eliciting resistant disease. Indeed, our results demonstrate that cancer interventions may benefit from concomitant suppression of TMEJ with CNHEJ to sustain therapeutic efficacy. Furthermore, our discovery of repeat sequence fluctuations in POLQ-deficient cells suggests that telomere or satellite tract measurements may prove practical and informative biomarkers of effective clinical POLQ inhibition. Thus, in accordance with the multifaceted nature of POLQ itself, the therapeutic targeting of TMEJ will have complex and context-specific repercussions that demand rigorous assessment. Investigations including those narrated in this paper constitute relevant and timely contributions to this compelling deliberation.

## DATA AVAILABILITY

All novel sequencing data relevant to this study have been deposited under BioProject PRJNA813416 at the SRA metadata portal (https://trace.ncbi.nlm.nih.gov/Traces/sra/).

## Supplementary Material

zcac020_Supplemental_Files

## References

[1] Seki M. , Marini F., Wood R.D. POLQ (Pol theta), a DNA polymerase and DNA-dependent ATPase in human cells. Nucleic Acids Res. 2003; 31:6117–6126. 14576298 10.1093/nar/gkg814 PMC275456

[2] Sharief F.S. , Vojta P.J., Ropp P.A., Copeland W.C. Cloning and chromosomal mapping of the human DNA polymerase theta (POLQ), the eighth human DNA polymerase. Genomics. 1999; 59:90–96. 10395804 10.1006/geno.1999.5843

[3] Arana M.E. , Seki M., Wood R.D., Rogozin I.B., Kunkel T.A. Low-fidelity DNA synthesis by human DNA polymerase theta. Nucleic Acids Res. 2008; 36:3847–3856. 18503084 10.1093/nar/gkn310 PMC2441791

[4] Hogg M. , Seki M., Wood R.D., Doublié S., Wallace S.S. Lesion bypass activity of DNA polymerase θ (POLQ) is an intrinsic property of the pol domain and depends on unique sequence inserts. J. Mol. Biol. 2011; 405:642–652. 21050863 10.1016/j.jmb.2010.10.041 PMC3025778

[5] Seki M. , Masutani C., Yang L.W., Schuffert A., Iwai S., Bahar I., Wood R.D. High-efficiency bypass of DNA damage by human DNA polymerase Q. EMBO J. 2004; 23:4484–4494. 15496986 10.1038/sj.emboj.7600424 PMC526458

[6] Roerink S.F. , van Schendel R., Tijsterman M. Polymerase theta-mediated end joining of replication-associated DNA breaks in *C. elegans*. Genome Res. 2014; 24:954–962. 24614976 10.1101/gr.170431.113 PMC4032859

[7] van Schendel R. , van Heteren J., Welten R., Tijsterman M. Genomic scars generated by polymerase theta reveal the versatile mechanism of alternative end-joining. PLoS Genet. 2016; 12:e1006368. 27755535 10.1371/journal.pgen.1006368 PMC5068794

[8] Chan S.H. , Yu A.M., McVey M. Dual roles for DNA polymerase theta in alternative end-joining repair of double-strand breaks in *Drosophila*. PLoS Genet. 2010; 6:e1001005. 20617203 10.1371/journal.pgen.1001005 PMC2895639

[9] Kent T. , Chandramouly G., McDevitt S.M., Ozdemir A.Y., Pomerantz R.T. Mechanism of microhomology-mediated end-joining promoted by human DNA polymerase θ. Nat. Struct. Mol. Biol. 2015; 22:230–237. 25643323 10.1038/nsmb.2961 PMC4351179

[10] Seki M. , Wood R.D. DNA polymerase theta (POLQ) can extend from mismatches and from bases opposite a (6-4) photoproduct. DNA Repair. 2008; 7:119–127. 17920341 10.1016/j.dnarep.2007.08.005 PMC2185714

[11] Hogg M. , Sauer-Eriksson A.E., Johansson E. Promiscuous DNA synthesis by human DNA polymerase θ. Nucleic Acids Res. 2012; 40:2611–2622. 22135286 10.1093/nar/gkr1102 PMC3315306

[12] Mateos-Gomez P.A. , Gong F., Nair N., Miller K.M., Lazzerini-Denchi E., Sfeir A. Mammalian polymerase theta promotes alternative NHEJ and suppresses recombination. Nature. 2015; 518:254–257. 25642960 10.1038/nature14157 PMC4718306

[13] Schimmel J. , van Schendel R., den Dunnen J.T., Tijsterman M. Templated insertions: a smoking gun for polymerase theta-mediated end joining. Trends Genet. 2019; 35:632–644. 31296341 10.1016/j.tig.2019.06.001

[14] Wyatt D.W. , Feng W., Conlin M.P., Yousefzadeh M.J., Roberts S.A., Mieczkowski P., Wood R.D., Gupta G.P., Ramsden D.A. Essential roles for polymerase θ-mediated end joining in the repair of chromosome breaks. Mol. Cell. 2016; 63:662–673. 27453047 10.1016/j.molcel.2016.06.020 PMC4992412

[15] Chandramouly G. , Zhao J., McDevitt S., Rusanov T., Hoang T., Borisonnik N., Treddinick T., Lopezcolorado F.W., Kent T., Siddique L.A. et al. Polθ reverse transcribes RNA and promotes RNA-templated DNA repair. Sci. Adv. 2021; 7:eabf1771. 34117057 10.1126/sciadv.abf1771 PMC8195485

[16] Yoshimura M. , Kohzaki M., Nakamura J., Asagoshi K., Sonoda E., Hou E., Prasad R., Wilson S.H., Tano K., Yasui A. et al. Vertebrate POLQ and POLbeta cooperate in base excision repair of oxidative DNA damage. Mol. Cell. 2006; 24:115–125. 17018297 10.1016/j.molcel.2006.07.032 PMC1868411

[17] Fujimori H. , Hyodo M., Matsuno Y., Shimizu A., Minakawa Y., Atsumi Y., Nakatsu Y., Tsuzuki T., Murakami Y., Yoshioka K. Mismatch repair dependence of replication stress-associated DSB recognition and repair. Heliyon. 2019; 5:e03057. 32083205 10.1016/j.heliyon.2019.e03057 PMC7019108

[18] Ramsden D.A. , Carvajal-Garcia J., Gupta G.P. Mechanism, cellular functions and cancer roles of polymerase-theta-mediated DNA end joining. Nat. Rev. Mol. Cell Biol. 2021; 23:125–140. 34522048 10.1038/s41580-021-00405-2 PMC13537726

[19] Zahn K.E. , Jensen R.B. Polymerase θ coordinates multiple intrinsic enzymatic activities during DNA repair. Genes. 2021; 12:1310. 34573292 10.3390/genes12091310 PMC8470613

[20] Ceccaldi R. , Liu J.C., Amunugama R., Hajdu I., Primack B., Petalcorin M.I., O’Connor K.W., Konstantinopoulos P.A., Elledge S.J., Boulton S.J. et al. Homologous-recombination-deficient tumours are dependent on Polθ-mediated repair. Nature. 2015; 518:258–262. 25642963 10.1038/nature14184 PMC4415602

[21] Mateos-Gomez P.A. , Kent T., Deng S.K., McDevitt S., Kashkina E., Hoang T.M., Pomerantz R.T., Sfeir A. The helicase domain of Polθ counteracts RPA to promote alt-NHEJ. Nat. Struct. Mol. Biol. 2017; 24:1116–1123. 29058711 10.1038/nsmb.3494 PMC6047744

[22] Llorens-Agost M. , Ensminger M., Le H.P., Gawai A., Liu J., Cruz-García A., Bhetawal S., Wood R.D., Heyer W.D., Löbrich M. POLθ-mediated end joining is restricted by RAD52 and BRCA2 until the onset of mitosis. Nat. Cell Biol. 2021; 23:1095–1104. 34616022 10.1038/s41556-021-00764-0 PMC8675436

[23] Feng Z. , Scott S.P., Bussen W., Sharma G.G., Guo G., Pandita T.K., Powell S.N. Rad52 inactivation is synthetically lethal with BRCA2 deficiency. Proc. Natl Acad. Sci. U.S.A. 2011; 108:686–691. 21148102 10.1073/pnas.1010959107 PMC3021033

[24] Feng W. , Simpson D.A., Carvajal-Garcia J., Price B.A., Kumar R.J., Mose L.E., Wood R.D., Rashid N., Purvis J.E., Parker J.S. et al. Genetic determinants of cellular addiction to DNA polymerase theta. Nat. Commun. 2019; 10:4286. 31537809 10.1038/s41467-019-12234-1 PMC6753077

[25] Zhou J. , Gelot C., Pantelidou C., Li A., Yücel H., Davis R.E., Farkkila A., Kochupurakkal B., Syed A., Shapiro G.I. et al. A first-in-class polymerase theta inhibitor selectively targets homologous-recombination-deficient tumors. Nat. Cancer. 2021; 2:598–610. 34179826 10.1038/s43018-021-00203-x PMC8224818

[26] Allera-Moreau C. , Rouquette I., Lepage B., Oumouhou N., Walschaerts M., Leconte E., Schilling V., Gordien K., Brouchet L., Delisle M.B. et al. DNA replication stress response involving PLK1, CDC6, POLQ, RAD51 and CLASPIN upregulation prognoses the outcome of early/mid-stage non-small cell lung cancer patients. Oncogenesis. 2012; 1:e30. 23552402 10.1038/oncsis.2012.29 PMC3503291

[27] Higgins G.S. , Harris A.L., Prevo R., Helleday T., McKenna W.G., Buffa F.M. Overexpression of POLQ confers a poor prognosis in early breast cancer patients. Oncotarget. 2010; 1:175–184. 20700469 10.18632/oncotarget.124 PMC2917771

[28] Zatreanu D. , Robinson H.M.R., Alkhatib O., Boursier M., Finch H., Geo L., Grande D., Grinkevich V., Heald R.A., Langdon S. et al. Polθ inhibitors elicit BRCA-gene synthetic lethality and target PARP inhibitor resistance. Nat. Commun. 2021; 12:3636. 34140467 10.1038/s41467-021-23463-8 PMC8211653

[29] Schrempf A. , Slyskova J., Loizou J.I. Targeting the DNA repair enzyme polymerase θ in cancer therapy. Trends Cancer. 2021; 7:98–111. 33109489 10.1016/j.trecan.2020.09.007

[30] Capper R. , Britt-Compton B., Tankimanova M., Rowson J., Letsolo B., Man S., Haughton M., Baird D.M. The nature of telomere fusion and a definition of the critical telomere length in human cells. Genes Dev. 2007; 21:2495–2508. 17908935 10.1101/gad.439107 PMC1993879

[31] Counter C.M. , Avilion A.A., LeFeuvre C.E., Stewart N.G., Greider C.W., Harley C.B., Bacchetti S. Telomere shortening associated with chromosome instability is arrested in immortal cells which express telomerase activity. EMBO J. 1992; 11:1921–1929. 1582420 10.1002/j.1460-2075.1992.tb05245.x PMC556651

[32] Preto A. , Singhrao S.K., Haughton M.F., Kipling D., Wynford-Thomas D., Jones C.J. Telomere erosion triggers growth arrest but not cell death in human cancer cells retaining wild-type p53: implications for antitelomerase therapy. Oncogene. 2004; 23:4136–4145. 15064743 10.1038/sj.onc.1207564

[33] Harley C.B. , Futcher A.B., Greider C.W. Telomeres shorten during ageing of human fibroblasts. Nature. 1990; 345:458–460. 2342578 10.1038/345458a0

[34] Lin T.T. , Norris K., Heppel N.H., Pratt G., Allan J.M., Allsup D.J., Bailey J., Cawkwell L., Hills R., Grimstead J.W. et al. Telomere dysfunction accurately predicts clinical outcome in chronic lymphocytic leukaemia, even in patients with early stage disease. Br. J. Haematol. 2014; 167:214–223. 24990087 10.1111/bjh.13023

[35] Murnane J.P. Telomeres and chromosome instability. DNA Repair. 2006; 5:1082–1092. 16784900 10.1016/j.dnarep.2006.05.030

[36] Artandi S.E. , Chang S., Lee S.L., Alson S., Gottlieb G.J., Chin L., DePinho R.A. Telomere dysfunction promotes non-reciprocal translocations and epithelial cancers in mice. Nature. 2000; 406:641–645. 10949306 10.1038/35020592

[37] Escudero L. , Cleal K., Ashelford K., Fegan C., Pepper C., Liddiard K., Baird D.M. Telomere fusions associate with coding sequence and copy number alterations in CLL. Leukemia. 2019; 33:2093–2097. 30796307 10.1038/s41375-019-0423-y PMC6690834

[38] Letsolo B.T. , Rowson J., Baird D.M. Fusion of short telomeres in human cells is characterized by extensive deletion and microhomology, and can result in complex rearrangements. Nucleic Acids Res. 2010; 38:1841–1852. 20026586 10.1093/nar/gkp1183 PMC2847243

[39] Riboni R. , Casati A., Nardo T., Zaccaro E., Ferretti L., Nuzzo F., Mondello C. Telomeric fusions in cultured human fibroblasts as a source of genomic instability. Cancer Genet. Cytogenet. 1997; 95:130–136. 9169029 10.1016/s0165-4608(96)00248-8

[40] Roger L. , Jones R.E., Heppel N.H., Williams G.T., Sampson J.R., Baird D.M. Extensive telomere erosion in the initiation of colorectal adenomas and its association with chromosomal instability. J. Natl Cancer Inst. 2013; 105:1202–1211. 23918447 10.1093/jnci/djt191

[41] Gisselsson D. , Jonson T., Petersén A., Strömbeck B., Dal Cin P., Höglund M., Mitelman F., Mertens F., Mandahl N. Telomere dysfunction triggers extensive DNA fragmentation and evolution of complex chromosome abnormalities in human malignant tumors. Proc. Natl Acad. Sci. U.S.A. 2001; 98:12683–12688. 11675499 10.1073/pnas.211357798 PMC60114

[42] Montalto M.C. , Phillips J.S., Ray F.A. Telomerase activation in human fibroblasts during escape from crisis. J. Cell. Physiol. 1999; 180:46–52. 10362016 10.1002/(SICI)1097-4652(199907)180:1<46::AID-JCP5>3.0.CO;2-K

[43] Ding Z. , Wu C.J., Jaskelioff M., Ivanova E., Kost-Alimova M., Protopopov A., Chu G.C., Wang G., Lu X., Labrot E.S. et al. Telomerase reactivation following telomere dysfunction yields murine prostate tumors with bone metastases. Cell. 2012; 148:896–907. 22341455 10.1016/j.cell.2012.01.039 PMC3629723

[44] Cahill D.P. , Kinzler K.W., Vogelstein B., Lengauer C. Genetic instability and Darwinian selection in tumours. Trends Cell Biol. 1999; 9:M57–M60. 10611684

[45] Bollen Y.A. , Stelloo E., van Leenen P.A., van den Bos M.A., Ponsioen B., Lu B.A., van Roosmalen M.J., Bolhaqueiro A.C.F., Kimberley C., Mossner M.A. et al. Reconstructing single-cell karyotype alterations in colorectal cancer identifies punctuated and gradual diversification patterns. Nat. Genet. 2021; 53:1187–1195. 34211178 10.1038/s41588-021-00891-2 PMC8346364

[46] Rosswog C. , Bartenhagen C., Welte A., Kahlert Y., Hemstedt N., Lorenz W., Cartolano M., Ackermann S., Perner S., Vogel W. et al. Chromothripsis followed by circular recombination drives oncogene amplification in human cancer. Nat. Genet. 2021; 53:1673–1685. 34782764 10.1038/s41588-021-00951-7

[47] Shoshani O. , Brunner S.F., Yaeger R., Ly P., Nechemia-Arbely Y., Kim D.H., Fang R., Castillon G.A., Yu M., Li J.S.Z. et al. Chromothripsis drives the evolution of gene amplification in cancer. Nature. 2021; 591:137–141. 33361815 10.1038/s41586-020-03064-z PMC7933129

[48] Cleal K. , Jones R.E., Grimstead J.W., Hendrickson E.A., Baird D.M. Chromothripsis during telomere crisis is independent of NHEJ, and consistent with a replicative origin. Genome Res. 2019; 29:737–749. 30872351 10.1101/gr.240705.118 PMC6499312

[49] O’Hagan R.C. , Chang S., Maser R.S., Mohan R., Artandi S.E., Chin L., DePinho R.A. Telomere dysfunction provokes regional amplification and deletion in cancer genomes. Cancer Cell. 2002; 2:149–155. 12204535 10.1016/s1535-6108(02)00094-6

[50] Maciejowski J. , Li Y., Bosco N., Campbell P.J., de Lange T Chromothripsis and kataegis induced by telomere crisis. Cell. 2015; 163:1641–1654. 26687355 10.1016/j.cell.2015.11.054 PMC4687025

[51] Jones R.E. , Oh S., Grimstead J.W., Zimbric J., Roger L., Heppel N.H., Ashelford K.E., Liddiard K., Hendrickson E.A., Baird D.M. Escape from telomere-driven crisis is DNA ligase III dependent. Cell Rep. 2014; 8:1063–1076. 25127141 10.1016/j.celrep.2014.07.007

[52] Liddiard K. , Ruis B., Takasugi T., Harvey A., Ashelford K.E., Hendrickson E.A., Baird D.M. Sister chromatid telomere fusions, but not NHEJ-mediated inter-chromosomal telomere fusions, occur independently of DNA ligases 3 and 4. Genome Res. 2016; 26:588–600. 26941250 10.1101/gr.200840.115 PMC4864465

[53] Liddiard K. , Ruis B., Kan Y., Cleal K., Ashelford K.E., Hendrickson E.A., Baird D.M. DNA ligase 1 is an essential mediator of sister chromatid telomere fusions in G2 cell cycle phase. Nucleic Acids Res. 2019; 47:2402–2424. 30590694 10.1093/nar/gky1279 PMC6411840

[54] Tankimanova M. , Capper R., Letsolo B.T., Rowson J., Jones R.E., Britt-Compton B., Taylor A.M., Baird D.M. Mre11 modulates the fidelity of fusion between short telomeres in human cells. Nucleic Acids Res. 2012; 40:2518–2526. 22139912 10.1093/nar/gkr1117 PMC3315324

[55] Doksani Y. , de Lange T. Telomere-internal double-strand breaks are repaired by homologous recombination and PARP1/Lig3-dependent end-joining. Cell Rep. 2016; 17:1646–1656. 27806302 10.1016/j.celrep.2016.10.008 PMC5125555

[56] Chan S.W. , Blackburn E.H. Telomerase and ATM/Tel1p protect telomeres from nonhomologous end joining. Mol. Cell. 2003; 11:1379–1387. 12769860 10.1016/s1097-2765(03)00174-6

[57] Davoli T. , de Lange T. Telomere-driven tetraploidization occurs in human cells undergoing crisis and promotes transformation of mouse cells. Cancer Cell. 2012; 21:765–776. 22698402 10.1016/j.ccr.2012.03.044 PMC3376354

[58] Smogorzewska A. , Karlseder J., Holtgreve-Grez H., Jauch A., de Lange T. DNA ligase IV-dependent NHEJ of deprotected mammalian telomeres in G1 and G2. Curr. Biol. 2002; 12:1635–1644. 12361565 10.1016/s0960-9822(02)01179-x

[59] Ghezraoui H. , Piganeau M., Renouf B., Renaud J.B., Sallmyr A., Ruis B., Oh S., Tomkinson A.E., Hendrickson E.A., Giovannangeli C. et al. Chromosomal translocations in human cells are generated by canonical nonhomologous end-joining. Mol. Cell. 2014; 55:829–842. 25201414 10.1016/j.molcel.2014.08.002 PMC4398060

[60] Hendrickson E.A. , Baird D.M. Alternative end joining, clonal evolution, and escape from a telomere-driven crisis. Mol. Cell. Oncol. 2015; 2:e975623. 27308409 10.4161/23723556.2014.975623 PMC4905247

[61] Simsek D. , Brunet E., Wong S.Y., Katyal S., Gao Y., McKinnon P.J., Lou J., Zhang L., Li J., Rebar E.J. et al. DNA ligase III promotes alternative nonhomologous end-joining during chromosomal translocation formation. PLoS Genet. 2011; 7:e1002080. 21655080 10.1371/journal.pgen.1002080 PMC3107202

[62] Kawamura K. , Bahar R., Seimiya M., Chiyo M., Wada A., Okada S., Hatano M., Tokuhisa T., Kimura H., Watanabe S. et al. DNA polymerase theta is preferentially expressed in lymphoid tissues and upregulated in human cancers. Int. J. Cancer. 2004; 109:9–16. 14735462 10.1002/ijc.11666

[63] Shima N. , Munroe R.J., Schimenti J.C. The mouse genomic instability mutation chaos1 is an allele of Polq that exhibits genetic interaction with Atm. Mol. Cell. Biol. 2004; 24:10381–10389. 15542845 10.1128/MCB.24.23.10381-10389.2004 PMC529050

[64] Li Y. , Schwab C., Ryan S., Papaemmanuil E., Robinson H.M., Jacobs P., Moorman A.V., Dyer S., Borrow J., Griffiths M. et al. Constitutional and somatic rearrangement of chromosome 21 in acute lymphoblastic leukaemia. Nature. 2014; 508:98–102. 24670643 10.1038/nature13115 PMC3976272

[65] Notta F. , Chan-Seng-Yue M., Lemire M., Li Y., Wilson G.W., Connor A.A., Denroche R.E., Liang S.B., Brown A.M., Kim J.C. et al. A renewed model of pancreatic cancer evolution based on genomic rearrangement patterns. Nature. 2016; 538:378–382. 27732578 10.1038/nature19823 PMC5446075

[66] Liddiard K. , Grimstead J.W., Cleal K., Evans A., Baird D.M. Tracking telomere fusions through crisis reveals conflict between DNA transcription and the DNA damage response. NAR Cancer. 2021; 3:zcaa044. 33447828 10.1093/narcan/zcaa044 PMC7787266

[67] Arai D. , Nakao Y. Efficient biallelic knock-in in mouse embryonic stem cells by *in vivo*-linearization of donor and transient inhibition of DNA polymerase θ/DNA-PK. Sci. Rep. 2021; 11:18132. 34518609 10.1038/s41598-021-97579-8 PMC8438075

[68] Saito S. , Maeda R., Adachi N. Dual loss of human POLQ and LIG4 abolishes random integration. Nat. Commun. 2017; 8:16112. 28695890 10.1038/ncomms16112 PMC5508229

[69] van Kregten M. , de Pater S., Romeijn R., van Schendel R., Hooykaas P.J., Tijsterman M. T-DNA integration in plants results from polymerase-θ-mediated DNA repair. Nat. Plants. 2016; 2:16164. 27797358 10.1038/nplants.2016.164

[70] Hofman I.J.F. , van Duin M., De Bruyne E., Fancello L., Mulligan G., Geerdens E., Garelli E., Mancini C., Lemmens H., Delforge M. et al. RPL5 on 1p22.1 is recurrently deleted in multiple myeloma and its expression is linked to bortezomib response. Leukemia. 2017; 31:1706–1714. 27909306 10.1038/leu.2016.370 PMC5380219

[71] Leach D.R. Long DNA palindromes, cruciform structures, genetic instability and secondary structure repair. Bioessays. 1994; 16:893–900. 7840768 10.1002/bies.950161207

[72] Akgün E. , Zahn J., Baumes S., Brown G., Liang F., Romanienko P.J., Lewis S., Jasin M. Palindrome resolution and recombination in the mammalian germ line. Mol. Cell. Biol. 1997; 17:9. 10.1128/mcb.17.9.5559 PMC232404 9271431

[73] Lührmann A. , Thölke J., Behn I., Schumann J., Tiegs G., Hauschildt S. Immunomodulating properties of the antibiotic novobiocin in human monocytes. Antimicrob. Agents Chemother. 1998; 42:1911–1916. 9687383 10.1128/aac.42.8.1911 PMC105709

[74] Quinlan A.R. , Hall I.M. BEDTools: a flexible suite of utilities for comparing genomic features. Bioinformatics. 2010; 26:841–842. 20110278 10.1093/bioinformatics/btq033 PMC2832824

[75] Rodriguez-Martin B. , Alvarez E.G., Baez-Ortega A., Zamora J., Supek F., Demeulemeester J., Santamarina M., Ju Y.S., Temes J., Garcia-Souto D. et al. Pan-cancer analysis of whole genomes identifies driver rearrangements promoted by LINE-1 retrotransposition. Nat. Genet. 2020; 52:306–319. 32024998 10.1038/s41588-019-0562-0 PMC7058536

[76] Subramanian A. , Tamayo P., Mootha V.K., Mukherjee S., Ebert B.L., Gillette M.A., Paulovich A., Pomeroy S.L., Golub T.R., Lander E.S. et al. Gene set enrichment analysis: a knowledge-based approach for interpreting genome-wide expression profiles. Proc. Natl Acad. Sci. U.S.A. 2005; 102:15545–15550. 16199517 10.1073/pnas.0506580102 PMC1239896

[77] Li Y. , Wang Q., Ning N., Tang F., Wang Y. Bioinformatic analysis reveals MIR502 as a potential tumour suppressor in ovarian cancer. J. Ovarian Res. 2020; 13:77. 32660514 10.1186/s13048-020-00683-y PMC7359466

[78] Wang Z. , Deng M., Chen L., Wang W., Liu G., Liu D., Han Z., Zhou Y. Circular RNA circ-03955 promotes epithelial–mesenchymal transition in osteosarcoma by regulating miR-3662/metadherin pathway. Front. Oncol. 2020; 10:545460. 33312941 10.3389/fonc.2020.545460 PMC7708376

[79] Adlakha Y.K. , Saini N. Brain microRNAs and insights into biological functions and therapeutic potential of brain enriched miRNA-128. Mol. Cancer. 2014; 13:33. 24555688 10.1186/1476-4598-13-33 PMC3936914

[80] Zylka M.J. , Simon J.M., Philpot B.D. Gene length matters in neurons. Neuron. 2015; 86:353–355. 25905808 10.1016/j.neuron.2015.03.059 PMC4584405

[81] Wei P.C. , Chang A.N., Kao J., Du Z., Meyers R.M., Alt F.W., Schwer B. Long neural genes harbor recurrent DNA break clusters in neural stem/progenitor cells. Cell. 2016; 164:644–655. 26871630 10.1016/j.cell.2015.12.039 PMC4752721

[82] Tabarés-Seisdedos R. , Rubenstein J.L.R. Chromosome 8p as a potential hub for developmental neuropsychiatric disorders: implications for schizophrenia, autism and cancer. Mol. Psychiatry. 2009; 14:563–589. 19204725 10.1038/mp.2009.2

[83] Amato J. , Cerofolini L., Brancaccio D., Giuntini S., Iaccarino N., Zizza P., Iachettini S., Biroccio A., Novellino E., Rosato A. et al. Insights into telomeric G-quadruplex DNA recognition by HMGB1 protein. Nucleic Acids Res. 2019; 47:9950–9966. 31504744 10.1093/nar/gkz727 PMC6765150

[84] Szklarczyk D. , Gable A.L., Lyon D., Junge A., Wyder S., Huerta-Cepas J., Simonovic M., Doncheva N.T., Morris J.H., Bork P. et al. STRING v11: protein–protein association networks with increased coverage, supporting functional discovery in genome-wide experimental datasets. Nucleic Acids Res. 2019; 47:D607D613. 30476243 10.1093/nar/gky1131 PMC6323986

[85] Yousefzadeh M.J. , Wyatt D.W., Takata K., Mu Y., Hensley S.C., Tomida J., Bylund G.O., Doublié S., Johansson E., Ramsden D.A. et al. Mechanism of suppression of chromosomal instability by DNA polymerase POLQ. PLoS Genet. 2014; 10:e1004654. 25275444 10.1371/journal.pgen.1004654 PMC4183433

[86] Garsed D.W. , Marshall O.J., Corbin V.D., Hsu A., Di Stefano L., Schröder J., Li J., Feng Z.P., Kim B.W., Kowarsky M. et al. The architecture and evolution of cancer neochromosomes. Cancer Cell. 2014; 26:653–667. 25517748 10.1016/j.ccell.2014.09.010

[87] Carvajal-Garcia J. , Crown K.N., Ramsden D.A., Sekelsky J. DNA polymerase theta suppresses mitotic crossing over. PLoS Genet. 2021; 17:e1009267. 33750946 10.1371/journal.pgen.1009267 PMC8016270

[88] Altemose N. , Miga K., Willard H. Genomic characterization of large heterochromatic gaps in the human genome assembly. PLoS Comput. Biol. 2014; 10:e1003628. 24831296 10.1371/journal.pcbi.1003628 PMC4022460

[89] Podugolnikova O.A. , Korostelev A.P. The quantitative analysis of polymorphism on human chromosomes 1, 9, 16, and Y. IV. Heterogeneity of a normal population. Hum. Genet. 1980; 54:163–169. 7190127 10.1007/BF00278966

[90] Smith G.P. Evolution of repeated DNA sequences by unequal crossover. Science. 1976; 191:528–535. 1251186 10.1126/science.1251186

[91] Eickbush T.H. , Eickbush D.G. Finely orchestrated movements: evolution of the ribosomal RNA genes. Genetics. 2007; 175:477–485. 17322354 10.1534/genetics.107.071399 PMC1800602

[92] Kobayashi T. , Heck D.J., Nomura M., Horiuchi T. Expansion and contraction of ribosomal DNA repeats in *Saccharomyces cerevisiae*: requirement of replication fork blocking (Fob1) protein and the role of RNA polymerase I. Genes Dev. 1998; 12:3821–3830. 9869636 10.1101/gad.12.24.3821 PMC317266

[93] Salim D. , Bradford W.D., Freeland A., Cady G., Wang J., Pruitt S.C., Gerton J.L. DNA replication stress restricts ribosomal DNA copy number. PLoS Genet. 2017; 13:e1007006. 28915237 10.1371/journal.pgen.1007006 PMC5617229

[94] Carvajal-Garcia J. , Cho J.E., Carvajal-Garcia P., Feng W., Wood R.D., Sekelsky J., Gupta G.P., Roberts S.A., Ramsden D.A. Mechanistic basis for microhomology identification and genome scarring by polymerase theta. Proc. Natl Acad. Sci. U.S.A. 2020; 117:8476–8485. 32234782 10.1073/pnas.1921791117 PMC7165422

[95] Davis L. , Khoo K.J., Zhang Y., Maizels N. POLQ suppresses interhomolog recombination and loss of heterozygosity at targeted DNA breaks. Proc. Natl Acad. Sci. U.S.A. 2020; 117:22900–22909. 32873648 10.1073/pnas.2008073117 PMC7502765

[96] Baldacci G. , Hoffmann J.S., Cadoret J.C. Impact of the DNA polymerase theta on the DNA replication program. Genomics Data. 2015; 3:90–93. 26484154 10.1016/j.gdata.2014.11.014 PMC4535461

[97] Fernandez-Vidal A. , Guitton-Sert L., Cadoret J.C., Drac M., Schwob E., Baldacci G., Cazaux C., Hoffmann J.S. A role for DNA polymerase θ in the timing of DNA replication. Nat. Commun. 2014; 5:4285. 24989122 10.1038/ncomms5285

[98] Lezaja A. , Panagopoulos A., Wen Y., Carvalho E., Imhof R., Altmeyer M. RPA shields inherited DNA lesions for post-mitotic DNA synthesis. Nat. Commun. 2021; 12:3827. 34158486 10.1038/s41467-021-23806-5 PMC8219667

[99] Orthwein A. , Fradet-Turcotte A., Noordermeer S.M., Canny M.D., Brun C.M., Strecker J., Escribano-Diaz C., Durocher D. Mitosis inhibits DNA double-strand break repair to guard against telomere fusions. Science. 2014; 344:189–193. 24652939 10.1126/science.1248024

[100] Clay D.E. , Bretscher H.S., Jezuit E.A., Bush K.B., Fox D.T. Persistent DNA damage signaling and DNA polymerase theta promote broken chromosome segregation. J. Cell Biol. 2021; 220:e202106116. 34613334 10.1083/jcb.202106116 PMC8500225

[101] Bielski C.M. , Zehir A., Penson A.V., Donoghue M.T.A., Chatila W., Armenia J., Chang M.T., Schram A.M., Jonsson P., Bandlamudi C. et al. Genome doubling shapes the evolution and prognosis of advanced cancers. Nat. Genet. 2018; 50:1189–1195. 30013179 10.1038/s41588-018-0165-1 PMC6072608

[102] Shapiro J.A. How chaotic is genome chaos?. Cancers (Basel). 2021; 13:1358. 33802828 10.3390/cancers13061358 PMC8002653

[103] Henriksen R.A. , Jenjaroenpun P., Sjøstrøm I.B., Jensen K.R., Prada-Luengo I., Wongsurawat T., Nookaew I., Regenberg B. Circular DNA in the human germline and its association with recombination. Mol. Cell. 2021; 82:209–219. 34951964 10.1016/j.molcel.2021.11.027 PMC10707452

[104] Uphoff S. , Lord N.D., Okumus B., Potvin-Trottier L., Sherratt D.J., Paulsson J. Stochastic activation of a DNA damage response causes cell-to-cell mutation rate variation. Science. 2016; 351:1094–1097. 26941321 10.1126/science.aac9786 PMC4827329

[105] Umbreit N.T. , Zhang C.Z., Lynch L.D., Blaine L.J., Cheng A.M., Tourdot R., Sun L., Almubarak H.F., Judge K., Mitchell T.J. et al. Mechanisms generating cancer genome complexity from a single cell division error. Science. 2020; 368:eaba0712. 32299917 10.1126/science.aba0712 PMC7347108

[106] Rai R. , Gu P., Broton C., Kumar-Sinha C., Chen Y., Chang S. The replisome mediates A-NHEJ repair of telomeres lacking POT1-TPP1 independently of MRN function. Cell Rep. 2019; 29:3708–3725. 31825846 10.1016/j.celrep.2019.11.012 PMC7001145

[107] Anand R. , Buechelmaier E., Belan O., Newton M., Vancevska A., Kaczmarczyk A., Takaki T., Rueda D.S., Powell S.N., Boulton S.J. HELQ is a dual-function DSB repair enzyme modulated by RPA and RAD51. Nature. 2022; 601:268–273. 34937945 10.1038/s41586-021-04261-0 PMC8755542

[108] Hussmann J.A. , Ling J., Ravisankar P., Yan J., Cirincione A., Xu A., Simpson D., Yang D., Bothmer A., Cotta-Ramusino C. et al. Mapping the genetic landscape of DNA double-strand break repair. Cell. 2021; 184:5653–5669. 34672952 10.1016/j.cell.2021.10.002 PMC9074467

[109] Hussain S.S. , Majumdar R., Moore G.M., Narang H., Buechelmaier E.S., Bazil M.J., Ravindran P.T., Leeman J.E., Li Y., Jalan M. et al. Measuring nonhomologous end-joining, homologous recombination and alternative end-joining simultaneously at an endogenous locus in any transfectable human cell. Nucleic Acids Res. 2021; 49:e74. 33877327 10.1093/nar/gkab262 PMC8287935

[110] Kamp J.A. , Lemmens B.B.L.G., Romeijn R.J., Changoer S.C., van Schendel R., Tijsterman M. Helicase Q promotes homology-driven DNA double-strand break repair and prevents tandem duplications. Nat. Commun. 2021; 12:7126. 34880204 10.1038/s41467-021-27408-z PMC8654963

[111] Oh S. , Harvey A., Zimbric J., Wang Y., Nguyen T., Jackson P.J., Hendrickson E.A. DNA ligase III and DNA ligase IV carry out genetically distinct forms of end joining in human somatic cells. DNA Repair. 2014; 21:97–110. 24837021 10.1016/j.dnarep.2014.04.015 PMC4125535

[112] Kabotyanski E.B. , Gomelsky L., Han J.O., Stamato T.D., Roth D.B. Double-strand break repair in Ku86- and XRCC4-deficient cells. Nucleic Acids Res. 1998; 26:5333–5342. 9826756 10.1093/nar/26.23.5333 PMC147996

[113] Verma P. , Greenberg R.A. Noncanonical views of homology-directed DNA repair. Genes Dev. 2016; 30:1138–1154. 27222516 10.1101/gad.280545.116 PMC4888836

[114] Elliott B. , Richardson C., Jasin M. Chromosomal translocation mechanisms at intronic Alu elements in mammalian cells. Mol. Cell. 2005; 17:885–894. 15780943 10.1016/j.molcel.2005.02.028

[115] Schimmel J. , Kool H., van Schendel R., Tijsterman M. Mutational signatures of non-homologous and polymerase theta-mediated end-joining in embryonic stem cells. EMBO J. 2017; 36:3634–3649. 29079701 10.15252/embj.201796948 PMC5730883

[116] Bhargava R. , Sandhu M., Muk S.A., Lee G., Vaidehi N., Stark J.A. C-NHEJ without indels is robust and requires synergistic function of distinct XLF domains. Nat. Commun. 2018; 9:2484. 29950655 10.1038/s41467-018-04867-5 PMC6021437

[117] Hwang T. , Reh S., Dunbayev Y., Zhong Y., Takata Y., Shen J., McBride K.M., Murnane J.P., Bhak J., Lee S. et al. Defining the mutation signatures of DNA polymerase θ in cancer genomes. NAR Cancer. 2020; 2:zcaa017. 32885167 10.1093/narcan/zcaa017 PMC7454005

[118] Schlötterer C. , Tautz D. Slippage synthesis of simple sequence DNA. Nucleic Acids Res. 1992; 20:211–215. 1741246 10.1093/nar/20.2.211 PMC310356

[119] Yilmaz D. , Furst A., Meaburn K., Lezaja A., Wen Y., Altmeyer M., Reina-San-Martin B., Soutoglou E. Activation of homologous recombination in G1 preserves centromeric integrity. Nature. 2021; 600:748–753. 34853474 10.1038/s41586-021-04200-z

[120] Masuda K. , Ouchida R., Hikida M., Kurosaki T., Yokoi M., Masutani C., Seki M., Wood R.D., Hanaoka F., Wang J. DNA polymerases eta and theta function in the same genetic pathway to generate mutations at A/T during somatic hypermutation of Ig genes. J. Biol. Chem. 2007; 282:17387–17394. 17449470 10.1074/jbc.M611849200

[121] McNulty S.M. , Sullivan B.A. Alpha satellite DNA biology: finding function in the recesses of the genome. Chromosome Res. 2018; 26:115–138. 29974361 10.1007/s10577-018-9582-3 PMC6121732

[122] Kamp J.A. , van Schendel R., Dilweg I.W., Tijsterman M BRCA1-associated structural variations are a consequence of polymerase theta-mediated end-joining. Nat. Commun. 2020; 11:3615. 32680986 10.1038/s41467-020-17455-3 PMC7368036

[123] Larocque J.R. , Jasin M Mechanisms of recombination between diverged sequences in wild-type and BLM-deficient mouse and human cells. Mol. Cell. Biol. 2010; 30:1887–1897. 20154148 10.1128/MCB.01553-09 PMC2849462

[124] LaRocque J.R. , Stark J.M., Oh J., Bojilova E., Yusa K., Horie K., Takeda J., Jasin M Interhomolog recombination and loss of heterozygosity in wild-type and Bloom syndrome helicase (BLM)-deficient mammalian cells. Proc. Natl Acad. Sci. U.S.A. 2011; 108:11971–11976. 21730139 10.1073/pnas.1104421108 PMC3141969

[125] Gangloff S. , Zou H., Rothstein R Gene conversion plays the major role in controlling the stability of large tandem repeats in yeast. EMBO J. 1996; 15:1715–1725. 8612596 PMC450084

[126] Zelensky A.N. , Schimmel J., Kool H., Kanaar R., Tijsterman M Inactivation of Pol θ and C-NHEJ eliminates off-target integration of exogenous DNA. Nat. Commun. 2017; 8:66. 28687761 10.1038/s41467-017-00124-3 PMC5501794

[127] Gu P. , Jia S., Takasugi T.A., Tesmer V.M., Nandakumar J.A., Chen Y.A., Chang S.A Distinct functions of POT1 proteins contribute to the regulation of telomerase recruitment to telomeres. Nat. Commun. 2021; 12:5514. 34535663 10.1038/s41467-021-25799-7 PMC8448735

[128] Gu P. , Wang Y., Bisht K.K., Wu L., Kukova L., Smith E.M., Xiao Y., Bailey S.M., Lei M., Nandakumar J. et al. Pot1 OB-fold mutations unleash telomere instability to initiate tumorigenesis. Oncogene. 2017; 36:1939–1951. 27869160 10.1038/onc.2016.405 PMC5383532

[129] Kratz K. , de Lange T Protection of telomeres 1 proteins POT1a and POT1b can repress ATR signaling by RPA exclusion, but binding to CST limits ATR repression by POT1b. J. Biol. Chem. 2018; 293:14384–14392. 30082315 10.1074/jbc.RA118.004598 PMC6139565

[130] Barroso-González J. , García-Expósito L., Galaviz P., Lynskey M.L., Allen J.A.M., Hoang S., Watkins S.C., Pickett H.A., O’Sullivan R.J Anti-recombination function of MutSα restricts telomere extension by ALT-associated homology-directed repair. Cell Rep. 2021; 37:110088. 34879271 10.1016/j.celrep.2021.110088 PMC8724847

[131] Jiricny J The multifaceted mismatch-repair system. Nat. Rev. Mol. Cell Biol. 2006; 7:335–346. 16612326 10.1038/nrm1907

[132] Kockler Z.W. , Osia B., Lee R., Musmaker K., Malkova A Repair of DNA breaks by break-induced replication. Annu. Rev. Biochem. 2021; 90:165–191. 33792375 10.1146/annurev-biochem-081420-095551 PMC9629446

[133] Osia B. , Alsulaiman T., Jackson T., Kramara J., Oliveira S., Malkova A Cancer cells are highly susceptible to accumulation of templated insertions linked to MMBIR. Nucleic Acids Res. 2021; 49:8714–8731. 34379776 10.1093/nar/gkab685 PMC8421209

[134] Schawalder J. , Paric E., Neff N.F Telomere and ribosomal DNA repeats are chromosomal targets of the bloom syndrome DNA helicase. BMC Cell Biol. 2003; 4:15. 14577841 10.1186/1471-2121-4-15 PMC270065

[135] Udugama M. , Sanij E., Voon H.P.J., Son J., Hii L., Henson J.D., Chan F.L., Chang F.T.M., Liu Y., Pearson R.B. et al. Ribosomal DNA copy loss and repeat instability in ATRX-mutated cancers. Proc. Natl Acad. Sci. U.S.A. 2018; 115:4737–4742. 29669917 10.1073/pnas.1720391115 PMC5939086

[136] Apte M.S. , Masuda H., Wheeler D.L., Cooper J.P RNAi and Ino80 complex control rate limiting translocation step that moves rDNA to eroding telomeres. Nucleic Acids Res. 2021; 49:8161–8176. 34244792 10.1093/nar/gkab586 PMC8373062

[137] Pich U. , Fuchs J., Schubert I How do Alliaceae stabilize their chromosome ends in the absence of TTTAGGG sequences?. Chromosome Res. 1996; 4:207–213. 8793205 10.1007/BF02254961

[138] Stimpson K.M. , Song I.Y., Jauch A., Holtgreve-Grez H., Hayden K.E., Bridger J.M., Sullivan B.A Telomere disruption results in non-random formation of *de novo* dicentric chromosomes involving acrocentric human chromosomes. PLoS Genet. 2010; 6:e1001061. 20711355 10.1371/journal.pgen.1001061 PMC2920838

[139] Wang Z. , Song Y., Li S., Kurian S., Xiang R., Chiba T., Wu X DNA polymerase θ (POLQ) is important for repair of DNA double-strand breaks caused by fork collapse. J. Biol. Chem. 2019; 294:3909–3919. 30655289 10.1074/jbc.RA118.005188 PMC6422074

[140] Chan K.Y. , Li X., Ortega J., Gu L., Li G.M DNA polymerase θ promotes CAG•CTG repeat expansions in Huntington’s disease via insertion sequences of its catalytic domain. J. Biol. Chem. 2021; 297:101144. 34473992 10.1016/j.jbc.2021.101144 PMC8463855

[141] Beagan K. , Armstrong R.L., Witsell A., Roy U., Renedo N., Baker A.E., Schärer O.D., McVey M *Drosophila* DNA polymerase theta utilizes both helicase-like and polymerase domains during microhomology-mediated end joining and interstrand crosslink repair. PLoS Genet. 2017; 13:e1006813. 28542210 10.1371/journal.pgen.1006813 PMC5466332

[142] Ozdemir A.Y. , Rusanov T., Kent T., Siddique L.A., Pomerantz R.T Polymerase θ-helicase efficiently unwinds DNA and RNA–DNA hybrids. J. Biol. Chem. 2018; 293:5259–5269. 29444826 10.1074/jbc.RA117.000565 PMC5892577

[143] Li J. , Ko J.M., Dai W., Yu V.Z., Ng H.Y., Hoffmann J.S., Lung M.L Depletion of DNA polymerase theta inhibits tumor growth and promotes genome instability through the cGAS–STING–ISG pathway in esophageal squamous cell carcinoma. Cancers (Basel). 2021; 13:3204. 34206946 10.3390/cancers13133204 PMC8268317

[144] Zahn K.E. , Jensen R.B., Wood R.D., Doublié S Human DNA polymerase θ harbors DNA end-trimming activity critical for DNA repair. Mol. Cell. 2021; 81:1534–1547. 33577776 10.1016/j.molcel.2021.01.021 PMC8231307

[145] Smith M.J. , Bryant E.E., Joseph F.J., Rothstein R DNA damage triggers increased mobility of chromosomes in G1-phase cells. Mol. Biol. Cell. 2019; 30:2620–2625. 31483739 10.1091/mbc.E19-08-0469 PMC6761769

[146] Higgins G.S. , Prevo R., Lee Y.F., Helleday T., Muschel R.J., Taylor S., Yoshimura M., Hickson I.D., Bernhard E.J., McKenna W.G A small interfering RNA screen of genes involved in DNA repair identifies tumor-specific radiosensitization by POLQ knockdown. Cancer Res. 2010; 70:2984–2993. 20233878 10.1158/0008-5472.CAN-09-4040 PMC2848966

[147] Turajlic S. , Litchfield K., Xu H., Rosenthal R., McGranahan N., Reading J.L., Wong Y.N.S., Rowan A., Kanu N., Al Bakir M et al. Insertion-and-deletion-derived tumour-specific neoantigens and the immunogenic phenotype: a pan-cancer analysis. Lancet Oncol. 2017; 18:1009–1021. 28694034 10.1016/S1470-2045(17)30516-8

[148] Qing T. , Jun T., Lindblad K.E., Lujambio A., Marczyk M., Pusztai L., Huang K.-l Diverse immune response of DNA damage repair-deficient tumors. Cell Rep. Med. 2021; 2:100276. 34095878 10.1016/j.xcrm.2021.100276 PMC8149377

[149] Pedersen B.S. , Quinlan A.R Mosdepth: quick coverage calculation for genomes and exomes. Bioinformatics. 2018; 34:867–868. 29096012 10.1093/bioinformatics/btx699 PMC6030888

