# Supplementary material for: POLQ suppresses genome instability and alterations in DNA repeat tract lengths
Source: NAR Cancer. 2022 Jun 29;4(3):zcac020. doi: 10.1093/narcan/zcac020 (PMC9241439; doi:10.1093/narcan/zcac020)
Supplement: zcac020_Supplemental_Files [file zcac020_supplemental_files.zip › Liddiard_POLQ_Supplementary_info_180522.docx]

**Supplementary Materials and Methods**

**Cells**

All cells were routinely cultured at 37°C in 5% CO_2_, screened for the absence of mycoplasma and authenticated by functional or expression assays appropriate to their specific genetic backgrounds.

**DNAR-deficient HCT116**

Datasets employing HCT116 harbouring targeted mutations in DNA ligases and repair components have been previously described (1-4).

**DNAR-deficient human fibroblasts**

Datasets employing human patient-derived fibroblasts with congenital mutations in DNA ligases have been detailed in our former study (5). The current manuscript was supplemented by data derived from patient fibroblasts bearing a mutation in *NBN* obtained from the Coriell Institute Cell Repository (GM 07166). GM 07166 fibroblasts were cultured in EMEM supplemented with 1 × non-essential amino acids, 15% (v/v) foetal calf serum, 1 x10^5^ IU/l penicillin, 100 mg/l streptomycin and 2 mM glutamine and buffered with 0.2% NaHCO_3_ solution.

The control fibroblast datasets have been comprehensively characterised (5) and comprise the four normal diploid lines MRC5, HCA2, IMR90 and WI38.

All fibroblasts were retrovirally transduced with HPV *E6E7* to promote senescence bypass (6).

**Nucleofection**

Subconfluent (60-80%) HCT116 and HAP1 WT and *POLQ^-/-^* cell lines were nucleofected using the Amaxa 4D Nucleofector with SE Cell Line reagents. Briefly, 1x10^6^ cells were nucleofected with 2.5 μg each TALEN plasmid or 1 μg control GFP expression vector (Lonza pmaxGFP) in 100 μl supplemented nucleofection reagent using program DS-138 (HCT116) or FF-113 (HAP1). Following 10 min recovery post-nucleofection in unsupplemented RPMI at 25°C and 15 min incubation in complete medium at 37°C, transfection replicates were pooled for co-culture to eliminate inter-sample variation. Cells were harvested at 24 or 48 hr post-nucleofection as indicated and pelleted for use in downstream assays.

**Lipofection**

HCT116, HAP1 and RPE1-hTERT were plated into 6 well tissue culture plates 24 hr prior to transfection at appropriate densities to achieve subconfluency (60-80%) at the point of transfection. Transfection master mixes were prepared using 5 μl [Lipofectamine™ 3000 Transfection Reagent](https://www.thermofisher.com/order/catalog/product/L3000001) (ThermoFisher Scientific) diluted in 125 μl OptiMEM (ThermoFisher Scientific) mixed 1:1 with 2.5 μg plasmid (TALEN or GFP) DNA diluted in OptiMEM and supplemented with 5 μl P3000 reagent. Following 5 minutes’ incubation at room temperature, lipid-DNA mixes were added dropwise to cells plated in complete medium. Cells were cultured with lipid-DNA complexes for 48 hr with medium replenishment after 24 hr without removal of transfection mix. Cells were harvested at 24 or 48 hr post-nucleofection as indicated and pelleted for use in downstream assays.

**Transfection efficiency**

GFP-transfected cells were supplemented with Hoechst 33342 to a final concentration of 10 μg/ml and incubated for 15 min at 37°C before propidium iodide was added to a final concentration of 10 μg/ml. GFP expression in viable cell populations was measured using the Chemometec NC-3000 image cytometer and extrapolated to the sample bulk as a transfection efficiency percentage.

**Sanger sequencing**

Verification of CRISPR/Cas9 targeting of *POLQ* and TALEN cleavage of subtelomeric sites was conducted by Sanger sequencing of locus-spanning gel-purified amplicons at Eurofins Genomics.

**RNA extraction and RT-PCR**

Total RNA was extracted using the Machery-Nagel Nucleospin RNA Plus kit with elution into nuclease-free water. Residual genomic DNA was eliminated using the Ambion DNA-*free*™ DNA Removal Kit and RNA concentrations and purity were assessed using a nanodrop spectrophotometer. For reverse transcription, 1 μg purified RNA was reacted in the presence or absence of reverse transcriptase using the Applied Biosystems High Capacity cDNA RT kit to distinguish true cDNA amplification from trace genomic DNA template. Equal volumes of cDNA products were subjected to 32 cycle RT-PCR using intron-spanning primers unique to each experimental tester gene at empirically-defined annealing temperatures.

# RT-PCR primers used in this study and the relative amplicon sizes are listed as Supplementary Table 8.

Amplicons were resolved by agarose (1% weight/volume) gel electrophoresis with ethidium bromide DNA staining and images were captured using a Vilbert Lourmat Ebox VX2 gel documenter. Quantification of amplicon signal intensity with local background correction was performed using ThermoFisher MyImageAnalysis software and standardised to values calculated for the constitutively-expressed *YWHAZ* control gene within the same samples. Normalised gene expression values are presented as tester/*YWHAZ* signal intensity or fold change in this parameter.

**Genomic DNA extraction**

Total genomic DNA was customarily extracted from WT and *POLQ^-/-^* cells using standard Tris-HCl lysis buffer supplemented with proteinase K and RNase A, followed by phenol/chloroform purification, precipitation with sodium acetate and solubilization in 10 mM Tris–HCl (pH 7.5). Total DNA was extracted from lipofected cells using Sigma GenElute™ Mammalian Genomic DNA Miniprep Kits and eluted in nuclease-free water. The purity and concentrations of all DNA samples were assessed by fluorometry and spectrophotometry.

**Telomere length PCR**

Telomere length at 17p (for HCT116 and RPE1-hTERT lines; HAP1 17p telomeres could not be amplified) and XpYp (for HAP1 and RPE1-hTERT lines; HCT116 possess an abridged XpYp subtelomere) was measured using the Single Telomere Length Analysis (STELA) protocol with chromosome-specific telomere-adjacent and Teltail primers (7). Amplicons were resolved by 0.5% TAE agarose gel electrophoresis and detected using radiolabelled telomere repeat or subtelomere probes following Southern blotting. Sizing and quantification of individual telomere products was performed using ImageQuant TL v 8.2 (Cytiva).

**Terminal Restriction Fragment analysis**

To estimate bulk population rather than individual telomere length, chromosome terminal DNA was resolved by 0.5% TAE agarose gel electrophoresis following digestion with *Hin*fI and *Rsa*I restriction enzymes at 37°C for more than 3 hr (8). Telomere terminal restriction fragments (9) were detected by Southern blotting using radiolabelled telomere repeat probes and quantification was performed using ImageQuant TL v 8.2 (Cytiva).

**Telomerase Repeated Amplification Protocol (TRAP)**

HCT116 and HAP1 WT and *POLQ^-/-^* clones were sampled pre-crisis to confirm the suppression of telomerase activity by the *DN-hTERT* expression cassette and post-crisis transition to determine the mechanism of telomere length stabilisation. Protein extracts were prepared using CHAPS lysis buffer (Millipore) and quantified using the Coomassie Plus Assay Kit (ThermoFisher Scientific). Telomere repeats amplified by 500ng each protein sample (10,11) were resolved by 40% acrylamide (19:1 acrylamide:bisacrylamide) gel electrophoresis and visualised with SYBR gold (ThermoFisher Scientific). Quantification was performed using ImageQuant TL v 8.2 (Cytiva) and standardised to the internal control.

Relative telomerase activity of each sample was calculated as a proportion of the positive control (telomerase-expressing cancer cell line). Crisis clones were subsequently compared to results obtained with the appropriate parental WT and *POLQ^-/-^* cell lines.

**Sequencing analysis**

Paired-end reads were aligned to GRCh38 using BWA-MEM (v0.7.1) (12) and processed using SAMtools (13).

***DN-hTERT* copy number estimates**

*DN-hTERT* copy numbers (CN) were inferred as described in (14) by analysing the read depth over exons at the *TERT* locus.

**Copy number variants (CNV) and structural variant (SV) calling**

CNVs were called as described in (14). Briefly, read depth was counted in 10kb non-overlapping windows genome wide, before de-noising using a Haar Wavelet transform (15), and normalizing to the GC % and mapability of the genomic bin. Relative CN changes were determined by background subtraction using the parental profile, or early-crisis profile. CN profiles were then segmented and heatmaps generated using the copyNumber package (16).

SVs were called using Dysgu (v1.1.7) (17), and common SV calls were identified by merging across samples using the ‘dysgu merge’ program. SV calls ≥ 300 bp in size were then further checked for uniqueness as described in (14). Briefly, each SV was checked by searching the alignment files of a pool of samples comprising ‘early-crisis’ samples from the same genetic background alongside the parental line. If a sample from the pool contained a read-pair supporting the SV in question, then the SV was labelled as non-unique. Non-unique SV calls and SV calls with a probability value < 0.2 determined by Dysgu were filtered.

**Clonality estimates**

Single Nucleotide Variants (SNVs) were first called using FreeBayes (v0.9.21) (18). SNVs were filtered by removing variants that were within ±10 bp of a simple repeat annotation (Gencode v27). Variants were also removed if they were multi-allelic sites, had a quality score < 30 or had a genotype that was not in (1/1, 0,1). Heterozygous variants were removed if they had an unbalanced mapping quality ratio between the reference and alternate alleles, defined as a MQM / MQMR ratio outside the range (0.5 - 1.5).

**Fusion calls**

Data were first aligned to GRCh38 using BWA-MEM (v0.7.1) (12). SV associated reads (discordant and split-reads) were separated using 'dysgu fetch’ command (parameters –mq 0 –clip-length 21 –max-coverage 100e3). Reads were then converted to fastq and trimmed using cutadapt (19) using the TelSeq telomere primers. Reads were then re-aligned to GRCh38 using BWA-MEM (parameters “-Y -P -a -I 290,90”) to generate all candidate alignments. Candidate alignments were reduced to a spanning set using the tool dodi (https://github.com/kcleal/dodi), using parameters –include subtelomere_loci.bed (5). The .bed file supplied to dodi lists the subtelomere loci associated with each TelSeq primer and is utilized when determining the optimal spanning set of alignments for each read pair. Telomere fusions were called using Dysgu (v1.1.6, with parameters “–mq 0 –min-support 1 –regions-only True –include subtelomere_loci.bed –max-cov 100000”). Common SV sites between samples were identified by running ‘dysgu merge’ command. A custom Gradient Boosting classifier implemented using sci-kit learn (20) was then applied to SV calls to prioritise variants.

**Intersection analysis**

Intersection analysis was performed with BEDTools (21), using the two-tailed *P*-values determined using the Fisher’s exact function.

**Micro satellite instability analysis**MSI sites were identified with MSIsensor-pro (22), comparing the parental WT and *POLQ^-/-^* lines. Candidate sites were further inspected using IGV (23).

**Satellite read depth**

Satellite regions of the Telomere2Telomere reference genome (Altmose, N. et al. *bioRxiv*, 2021.2007.2012.452052 and Nurk, S. et al. *bioRxiv*, 2021.2005.2026.445798) were separated into a separate fasta file for use as a mini reference genome. Data were then remapped to this mini reference using BWA-MEM (12) and filtered using SAMtools (13) with the flag -F 2308 to remove unmapped reads, non-primary alignments and supplementary alignments. Coverage values for each region were normalized to the sample mean, and log2 ratios were calculated to compare different samples.

**Sequence data display**

Indexed BAM files pertaining to all fusion amplicon sequencing and WGS samples were aligned to the GR38/hg38 human reference assembly and explored using the Broad Institute Integrative Genome Viewer (23). Custom tracks depicting Repeatmasker ([www.repeatmasker.org](http://www.repeatmasker.org/)) repetitive DNA motifs, fragile sites coding sequence were extracted as BED files from the UCSC Genome Browser tool (<https://genome.ucsc.edu/cgi-bin/hgTables>), the HUMCFS database (24) and Sanger Ensembl (25). Sequence-authenticated telomere fusion junctions, copy number variation (CNV) unique segments and crisis-induced structural variants (SV) were prepared as BED files for Ensembl karyotype plots, as well as IGV alignments.

**Gene Set Enrichment Analysis and STRING functional protein association networks**

Unique genes disrupted by genomic telomere fusions in WT and *POLQ^-/-^* crisis clones were assessed for pathway and functional enrichment using the Broad Institute Gene Set Enrichment Analysis (GSEA) tool (MSigDB 7.1; 2020) (26) and the STRING functional protein association network explorer (27).

**Statistical analyses**

All statistical analyses, including 2-tailed Mann–Whitney unpaired U-tests and Wilcoxon matched-pairs signed rank tests, Kruskal–Wallis and Repeated Measures ANOVA tests, Chi square and Fisher's exact tests were performed using GraphPad Prism 9 and GraphPad QuickCalcs. Comparison of means was computed using Medcalcs webtools. Additional evaluation of statistically significant enrichments in observations compared with representation in the human genome was performed using BEDtools v2.30.0 (21,28). Appropriate tests were selected according to whether data were collected as paired or independent experimental samples, whether data distributions were Gaussian or skewed and whether variance between comparators was equal. Data are presented as means with 95% confidence intervals (CI) except where it was considered important to display the spread of the data by standard deviation (SD). For all tests, a *P-*value of <0.05 was considered statistically significant.

**Programming**

Programming was conducted using Python 3 and R languages.

**Supplementary Figure Legends**

**Supplementary Figure 1: Characterisation of *POLQ^-/-^* human cell lines**

**Ai** Successful CRISPR/Cas9 targeting of *POLQ* in HCT116, HAP1 and RPE1-hTERT cells was confirmed by genotyping. A single nucleotide deletion at the target site in exon 2 was identified in HCT116 and RPE1-hTERT cells and a dinucleotide deletion in exon 1 resolved in HAP1 cells. Alignments of Sanger sequencing outputs for HCT116 and HAP1 *POLQ*-targeted cells with the GRCh38 genome assembly are presented. **ii** *POLQ* mRNA expression in unstimulated cells was measured by semi-quantitative RT-PCR using ThermoFisher MyImageAnalysis software to calculate background-corrected signal density normalised to *YWHAZ* control gene expression. Mean measurements for 3 replica experiments using WT and *POLQ^-/-^* HCT116, HAP1 and RPE1-hTERT cells are displayed with 95% CI (left panel). Summation of *POLQ* expression in WT and *POLQ^-/-^* cells of each lineage is depicted in the right panel. Mann-Whitney unpaired U-tests and Wilcoxon matched-pairs signed rank tests were conducted. **iii** Fold change in WT and *POLQ^-/-^* HCT116, HAP1 and RPE1-hTERT cell numbers over 24 hr following treatment with 5μM cisplatin (cis-diamminedichloroplatinum II). Data for cisplatin/untreated samples from duplicate experiments are shown as means with SD. **iv** *CDKN1A* mRNA expression in 24 hr unstimulated (- ; non-patterned bars) and 5μM cisplatin-treated (+ ; patterned bars) WT (black) and *POLQ^-/-^* (white) HCT116, HAP1 and RPE1-hTERT cells was measured by semi-quantitative RT-PCR using ThermoFisher MyImageAnalysis software to calculate background-corrected signal density normalised to *YWHAZ* control gene expression. Mean measurements for 2 replica experiments are displayed with SD. **Bi** 17p (dark blue) and XpYp (mid-blue) telomere lengths of HCT116, HAP1 and RPE1-hTERT WT (triangles) and *POLQ^-/-^* (crosses) cell lines evaluated using STELA and plotted with means and 95% CI displayed in red. Statistical significance was assessed by non-matched Brown-Forsyth and Welch’s ANOVA with Dunnett’s T3 multiple comparisons between samples. **ii** STELA measurements of 17p telomere lengths in HCT116 WT (triangles), *POLQ^-/-^* (crosses) and an array of other HCT116 DNAR-deficient models (grey star symbols). The two independent HCT116 WT sample groups are matched with the *POLQ^-/-^* or DNAR-deficient models. *LIG3^-/-^* cells were genetically complemented with *LIG3* WT and S847A or BRCT mutant alleles. Means with 95% CI are displayed with error bars in red and significance was tested using non-matched Brown-Forsyth and Welch’s ANOVA with Dunnett’s T3 multiple comparisons between columns. **iii** Presentation of pooled telomere length data for HCT116 WT (triangles), *POLQ^-/-^* (crosses) DNAR-deficient models (grey star symbols) with means and 95% CI in red. Non-matched Brown-Forsyth and Welch’s ANOVA with Dunnett’s T3 multiple comparisons between columns was performed. **iv** TRF blot revealing bulk telomere lengths (TL) in WT and *POLQ^-/-^* (*-/-*) HCT116, HAP1 and RPE1-hTERT cells with mean estimates featured below. **Ci** Relative telomerase activity in HCT116 and HAP1 WT (black bars) and *POLQ^-/-^* (white bars) clones surveyed at early and late crisis time points and quantified by TRAP assay. Mean signals as proportions of appropriate parental controls are charted with 95% CI. The passage of experimental time (growth phase) is illustrated with a black triangle and significant differences between time points were assessed using Wilcoxon matched-pairs signed rank tests. **ii** Exemplary TRAP assay blots for three clones (C; annotated below lanes) of each lineage at early (E) and late (L) crisis time points illustrate the increases in signal intensity with telomerase reactivation as clones escape crisis. Estimated *DN-hTERT* integration copy number in HCT116 (**iii**) and HAP1 (**iv**) WT (black) and *POLQ^-/-^* (white) clones at early (E) and late (L) crisis time points in comparison with parental lines (P) is presented. Copy number was computed from sequencing read coverage of the *DN-hTERT* transgene within genomic samples and is presented as means with 95% CI. Wilcoxon matched-pairs signed rank tests of paired early versus late crisis samples and Mann-Whitney unpaired U-tests between WT and *POLQ^-/-^* samples of each lineage were conducted. **D** Crisis profiles of individual **i** HCT116 WT, **ii** HCT116 *POLQ^-/-^*, **iii** HAP1 WT and **iv** HAP1 *POLQ^-/-^* clones are displayed. Growth curves (population doubling over time from day 0) are shown in black measured against the left y axis. Telomere lengths at 17p for HCT116 or XpYp for HAP1 against population doubling are marked in blue and measured against the right y axis. **E** Mean total telomere erosion between PD22 and experiment termination is shown for HCT116 and HAP1 WT (black) and *POLQ^-/-^* (white) clones with 95% CI and Mann-Whitney unpaired U-tests. **F** Maximal fusion frequencies (based on diploid genome DNA inputs into fusion PCR) for all HCT116 (black bars) and HAP1 (grey bars) clones are displayed as means with 95% CI and statistical significance determined using Mann-Whitney unpaired U-tests.

**Supplementary Figure 2: Endonuclease-induced telomere fusions in *POLQ^-/-^* cell lines**

**Ai** Total 16p and 21q family inter-chromosomal telomere fusions detected in HCT116 and HAP1 WT (black) and *POLQ^-/-^* (white) cells nuclofected with TALEN pairs targeting these subtelomeres. DNA was harvested at 24 hr for the rapidly growing HAP1 cells and 48 hr for HCT116 cells and fusion frequency was calculated based on diploid genome DNA inputs into fusion PCR. Means with 95% CI from three replica experiments are exhibited and statistical significance was determined using Mann-Whitney unpaired U-tests. Transfection efficiency in **ii** nucleofected HCT116 (black) and HAP1 (grey) or **iii** lipofectamine transfected HCT116 (black), HAP1 (grey) and RPE1-hTERT (dark grey) cells appraised by image cytometry quantifications of GFP-expressing viable cells at **ii** 24 hr or **iii** 48 hr. Data are presented as means with 95% CI of the fold change in GFP-expressing fractions in *POLQ^-/-^* compared with WT cells of each lineage. Significance was tested using Mann-Whitney unpaired U-tests. **Bi** Target sequence of the bespoke Xp subtelomere TALEN pair designed and employed in this study according to the GRCh38 human reference. **ii** Example Southern blot revealing Xp and 21q TALEN-induced telomere fusions amplified from lipofectamine transfected HAP1 WT and *POLQ^-/-^* cells with or without a POLQ expression vector. Cells were harvested 48 hr post-transfection and two complementary fusion assays were conducted using different combinations of primers targeting subtelomeric sites either proximal or distal to the Xp TALEN cleavage site (left-hand annotations). Radiolabelled Xp or 21q subtelomere-specific probes were used to visualise the fusion amplicons (right-hand annotations). **Ci** Summation of 17p intra-chromosomal telomere fusions amplified from HCT116 WT (black) or *POLQ^-/-^* (white) cells lipofectamine transfected with 17p TALEN pairs in the presence or absence of a POLQ expression vector. Means with 95% CI derived from five independent experiments are presented and statistical significance was determined using Mann-Whitney unpaired U-tests. **ii** Transfection efficiency in 100 μM NVB-treated compared with untreated HCT116 WT (black) and *POLQ^-/-^* (white) cells calculated following quantification of proportions of GFP-expressing viable cells 48 hr post-transfection using image cytometry. Means with SD of two replicate experiments are displayed and significance was tested using Mann-Whitney unpaired U-tests. **iii** Pooled fold change in cell number data for all transfected samples pertaining to the HCT116 WT (black) or *POLQ^-/-^* (white) sample groups treated (+) or untreated (-) with 100 μM NVB for 48 hr post-transfection. Means with 95% CI are plotted and Wilcoxon matched-pairs ranked sign tests of untreated versus treated pairs performed alongside Mann-Whitney unpaired U-tests of WT versus *POLQ^-/-^* samples. **iv** 17p intra-chromosomal fusion frequencies in HCT116 WT (black) and *POLQ^-/-^* (white) cells lipofectamine transfected with 17p TALEN pairs in the presence or absence of a POLQ expression vector and 100μM novobiocin (NVB) for 48 hr with dose replenished after 24 hr. Data represents 2 biological and 2 technical replicates. Means with 95% CI are displayed and significance was assessed using Wilcoxon matched-pairs signed rank tests between untreated and treated sample pairs.

**Supplementary Figure 3: *POLQ^-/-^* clones exhibit reduced incidence of gross chromosomal aberrations**

Whole genome sequencing was executed for HCT116 and HAP1 WT and *POLQ^-/-^* parental cell lines and clones pre- and post-crisis. Copy Number Variations were called using a custom pipeline (14) and profiles were generated by performing background subtraction of parental from pre-crisis samples or pre-crisis from late-crisis samples.

Heatmaps display the relative copy number changes across **A** HCT116 and **B** HAP1 clones (left-hand annotations), where copy number gains and losses are featured in red and blue, respectively. Chromosomes are ordered left to right along the y axes. **Ci** The Ensembl Genome Browser webtool was employed to prepare karyotype plots depicting unique copy number segments for all HCT116 WT (green left arrowheads) and *POLQ^-/-^* (red right arrowheads) clones and all HAP1 WT (blue left arrowheads) and *POLQ^-/-^* (purple right arrowheads) clones. **ii** Mean total incidences of all CNV unique segments in HCT116 and HAP1 WT (black) and *POLQ^-/-^* (white) Early and Late crisis samples with 95% CI. Statistical significance of differences between paired Early and Late samples for each genotype was tested using Wilcoxon matched-pairs signed rank tests, whereas differences between WT and *POLQ^-/-^* clones were analysed using Mann-Whitney unpaired U-tests. **iii** Enumeration of copy number gains (black) and losses (white) in all HCT116 and HAP1 WT and *POLQ^-/-^* clones presented as mean values with SD and significance assessed using a Mann-Whitney unpaired U-test. **iv** Total incidences of all copy number gains and losses for all WT (black) and *POLQ^-/-^* (white) clones presented as mean values with SD for pooled HCT116 and HAP1 data.

**Supplementary Figure 4: *POLQ^-/-^* clones display fewer structural variants than WT**

Whole genome sequencing was executed for HCT116 and HAP1 WT and *POLQ^-/-^* parental cell lines and clones pre- and post-crisis. Structural Variants (SV) were called using Dysgu (17) and unique events were identified by cross-referencing alignment files for all samples. Events detected in parental lines were removed and a filtering threshold of probability > 0.2 was applied to exclude false positives. **A** The numbers of unique SV localised to specific chromosomes in WT (black) and *POLQ^-/-^* (white) **i** HCT116 and **ii** HAP1 crisis clones are displayed. **iii** The total numbers of unique SV identified in WT (black) and *POLQ^-/-^* (white) HCT116 and HAP1 crisis clones are charted (means with 95% CI) with statistical significance assessed using Mann-Whitney unpaired U-tests. **iv** The total numbers of unique SV within Late crisis WT (black) and *POLQ^-/-^* (white) HCT116 and HAP1 clones are displayed with means and 95% CI. The statistical significance of differences was evaluated by Mann-Whitney unpaired U-tests. **v** The proportions of distinct SV within clones are displayed in a stacked bar chart with significance evaluated using Fisher’s Exact Tests. **B** The Ensembl Genome Browser webtool was employed to prepare karyotype plots depicting unique SV for all HCT116 WT (green left arrowheads) and *POLQ^-/-^* (red right arrowheads) clones and all HAP1 WT (blue left arrowheads) and *POLQ^-/-^* (purple right arrowheads) clones.

**Supplementary Figure 5: *POLQ^-/-^* clones are predominantly polyclonal post-crisis**

Variant allele frequencies (VAF) for the non-reference allele at sites of copy number variation were calculated for all **A** HCT116 **i** WT and **ii** *POLQ^-/-^* and all **B** HAP1 **i** WT and **ii** *POLQ^-/-^* post-crisis clones according to a custom pipeline (14). The clone identities are indicated in the top left corners of each plot. A dashed red line demarcates the 0.5 VAF frequency indicative of a monoclonal culture. **C** The proportions of all HCT116 and HAP1 WT and *POLQ^-/-^* clones classified as monoclonal (dark blue) or polyclonal (light blue) post-crisis are displayed in a stacked bar chart.

**Supplementary Figure 6: POLQ-deficient cells harbour altered telomere fusion profiles**

**Ai** Total fusion calls from all HCT116 and HAP1 WT (black) and *POLQ^-/-^* (white) sequenced crisis clones presented as means with 95% CI and analysed using a parametric paired T-test. **ii** The proportions of all sequence-validated telomere fusions derived from HCT116 and HAP1 WT (black) and *POLQ^-/-^* (white) clones that can be classified as Genomic, Inter-chromosomal or Intra-chromosomal events are presented as means with 95% CI. Statistical significance was evaluated using Mann-Whitney unpaired U-tests. **B** The proportions of all intra-chromosomal fusions comprised of **i** 17p (dark grey) or 21q family (light grey) chromatids (HCT116) or **ii** 16p (mid grey) or 21q family chromatids (HAP1) are presented in stacked bar charts (left panels) and column charts of means with 95% CI (right panels) for WT and *POLQ^-/-^* clones. Mann-Whitney unpaired U-tests were used to compare WT and *POLQ^-/-^* clones; Wilcoxon matched-pairs signed rank tests were used to compare intra-chromosomal fusions involving distinct chromosome ends. **C** The amount of deletion (in kb) of each 21q chromatid involved in an intra-chromosomal telomere fusion amplified from WT (filled triangles) and *POLQ^-/-^* (crosses) HCT116 (black) and HAP1 (grey) clones or crisis fibroblasts (CF; filled circles) clones juxtaposed with chromatid deletion data pertaining to crisis fibroblasts derived from patients with *LIG4*, *LIG1* or *NBN* mutations (mt; dash symbols) (5). Data are displayed as means with 95% CI in red and statistical significance was analysed using Mann-Whitney unpaired U-tests.

**Supplementary Figure 7: Characteristics of telomere fusions with genomic locations amplified from *POLQ^-/-^* clones**

**A** The Ensembl Genome Browser webtool was employed to prepare karyotype plots depicting validated genomic telomere fusion junction locations for all HCT116 WT (green left arrowheads) and *POLQ^-/-^* (red right arrowheads) clones and all HAP1 WT (blue left arrowheads) and *POLQ^-/-^* (purple right arrowheads) clones. **B** The percentages of all genomic telomere fusions with coding sequence that occur within exons (black) or introns (grey) are presented as stacked bar charts for WT and *POLQ^-/-^* HCT116 and HAP1 clones. Significance was assessed using Fisher’s Exact Tests. **Ci** The lengths (in Mb) of all genes coincident with genomic telomere fusion junctions amplified from WT (filled triangles) and *POLQ^-/-^* (crosses) HCT116 (black) and HAP1 (grey) clones or control crisis fibroblasts (CF; filled circles) are displayed in a scatter plot with means and 95% CI. Mann-Whitney unpaired U-tests were utilised to compare datasets and data for WT (black) and *POLQ^-/-^* (white) clones are summarised in **ii**. **D** The numbers of genomic telomere fusions characterised in WT (black) and *POLQ^-/-^* (white) HCT116 and HAP1 crisis clones that do (+) or do not (-) overlap with LINE1 (L1) loci implicated in cancer(29) are plotted. The significance of the associations was determined using Fisher’s Exact Tests in BEDtools v2.30.0 (28).

**Supplementary Figure 8: Genes disrupted by telomere fusions are interconnected**

The identities of all genes disrupted by genomic telomere fusions in **A** WT and **B** *POLQ^-/-^* crisis clones were associated using the STRING protein interaction networks functional enrichment analysis webtool (27). **C** Expected (E) and Observed (O) STRING network interactions for WT and *POLQ^-/-^* crisis clones annotated with the protein-protein interaction enrichment p-value score are presented.

**Supplementary Figure 9: Altered satellite DNA content of POLQ-deficient cells**

**A** The proportions of genomic telomere fusions derived from WT (black) and *POLQ^-/-^* (white) HCT116 and HAP1 clones or crisis fibroblasts derived from patients with *LIG4*, *LIG1* or *NBN* mutations (mid grey tones) or controls (Crisis Fibroblasts; light grey) whose junctions coincide with ALR, STR or TAR repeats are displayed as means with 95% CI with significance appraised using Fisher’s Exact Tests. **Bi** Normalised mean read depths relating to HSAT1-5 repeats for WT (black) and *POLQ^-/-^* (white) HCT116 and HAP1 parental lines with 95% CI and statistical significance evaluated using Wilcoxon matched-pairs signed rank tests. **ii** Normalised mean read depths relating to HSAT2 and HSAT3 repeats for WT (black) and DNA ligase-deficient (grey tones) HCT116 parental lines with 95% CI. Mann-Whitney unpaired U-tests were used to test the statistical significance of results. **iii** Normalised mean read depths relating to rDNA repeats for WT (black) and *POLQ^-/-^* (white) HCT116 and HAP1 parental lines with 95% CI and statistical significance assessed using Wilcoxon matched-pairs signed rank tests.

**Supplementary Table 1: Read counts and sample time points**

Details of all novel whole genome sequencing (WGS) and Fusion amplicon sequencing samples analysed in this manuscript.

**Supplementary Table 2: Copy Number Variation (CNV) unique segments**

All unique CNV segments identified in HCT116 and HAP1 WT and *POLQ^-/-^* whole genome sequencing samples.

**Supplementary Table 3: Unique structural variants (SV) identified in crisis whole genome sequencing (WGS) samples exceeding probability threshold of 0.2**

Chromosomes involved in SV are annotated as ChrA and ChrB. A probability score is given for the event and the types of SV are detailed in the final column, DEL; deletion, INV; inversion, DUP; duplication, TRA; translocation, INS; insertion.

**Supplementary Table 4: Structural variants (SV) within 10 Mb of a genomic telomere fusion**

Details of genomic fusion junctions and associated unique SV identified within 10 Mb linear genomic distance. The types of SV are detailed in the final column, DEL; deletion, INV; inversion, DUP; duplication, TRA; translocation, INS; insertion.

**Supplementary Table 5: GSEA enrichments in WT fused genes list**

Gene Set Enrichment Analysis (26) of all genes disrupted by genomic telomere fusions in WT crisis samples.

**Supplementary Table 6: GSEA enrichments in *POLQ^-/-^*** **fused genes list**

Gene Set Enrichment Analysis (26) of all genes disrupted by genomic telomere fusions in *POLQ^-/-^* crisis samples.

**Supplementary Table 7: STRING keywords enriched in *POLQ^-/-^* fused gene network**

Keyword annotations for STRING (27) functional network enrichments identified for genes involved in telomere fusions in *POLQ^-/-^* clones.

**Supplementary Table 8: Primers used in this study**

**Supplementary References:**

1. Jones, R.E., Oh, S., Grimstead, J.W., Zimbric, J., Roger, L., Heppel, N.H., Ashelford, K.E., Liddiard, K., Hendrickson, E.A. and Baird, D.M. (2014) Escape from telomere-driven crisis is DNA ligase III dependent. *Cell Rep*, **8**, 1063-1076.

2. Harvey, A., Mielke, N., Grimstead, J.W., Jones, R.E., Nguyen, T., Mueller, M., Baird, D.M. and Hendrickson, E.A. (2018) PARP1 is required for preserving telomeric integrity but is dispensable for A-NHEJ. *Oncotarget*, **9**, 34821-34837.

3. Liddiard, K., Ruis, B., Takasugi, T., Harvey, A., Ashelford, K.E., Hendrickson, E.A. and Baird, D.M. (2016) Sister chromatid telomere fusions, but not NHEJ-mediated inter-chromosomal telomere fusions, occur independently of DNA ligases 3 and 4. *Genome Res*, **26**, 588-600.

4. Oh, S., Harvey, A., Zimbric, J., Wang, Y., Nguyen, T., Jackson, P.J. and Hendrickson, E.A. (2014) DNA ligase III and DNA ligase IV carry out genetically distinct forms of end joining in human somatic cells. *DNA Repair*, **21**, 97-110.

5. Liddiard, K., Grimstead, J.W., Cleal, K., Evans, A. and Baird, D.M. (2021) Tracking telomere fusions through crisis reveals conflict between DNA transcription and the DNA damage response. *NAR Cancer*, **3**, zcaa044.

6. Bond, J.A., Haughton, M.F., Rowson, J.M., Smith, P.J., Gire, V., Wynford-Thomas, D. and Wyllie, F.S. (1999) Control of replicative life span in human cells: barriers to clonal expansion intermediate between M1 senescence and M2 crisis. *Mol Cell Biol*, **19**, 3103-3114.

7. Baird, D.M., Rowson, J., Wynford-Thomas, D. and Kipling, D. (2003) Extensive allelic variation and ultrashort telomeres in senescent human cells. *Nat Genet*, **33**, 203-207.

8. Kimura, M., Stone R.C, Hunt, S.C., Skurnick, J., Lu, X., Cao, X., Harley, C.B. and Aviv, A. (2010) Measurement of telomere length by the Southern blot analysis of terminal restriction fragment lengths. *Nat Protoc,* **5**, 1596-1607.

9. Mender, I. and Shay, J.W. (2015) Telomere Restriction Fragment (TRF) Analysis. *Bio Protoc,* **5**, e1658.

10. Kim, N.W., Piatyszek, M.A., Prowse, K.R., Harley, C.B., West, M.D., Ho, P.L., Coviello, G.M., Wright, W.E., Weinrich, S.L. and Shay, J.W. (1994) Specific association of human telomerase activity with immortal cells and cancer. *Science*, **266**, 2011-2015.

11. Mender, I. and Shay, J.W. (2015) Telomerase Repeated Amplification Protocol (TRAP). *Bio protoc,* **5**, e1657.

12. Li, H. (2013) Aligning sequence reads, clone sequences and assembly contigs with BWA-MEM. *ArXiv*, **1303**.

13. Li, H., Handsaker, B., Wysoker, A., Fennell, T., Ruan, J., Homer, N., Marth, G., Abecasis, G., Durbin, R. and Subgroup, G.P.D.P. (2009) The Sequence Alignment/Map format and SAMtools. *Bioinformatics*, **25**, 2078-2079.

14. Cleal, K., Jones, R.E., Grimstead, J.W., Hendrickson, E.A. and Baird, D.M. (2019) Chromothripsis during telomere crisis is independent of NHEJ, and consistent with a replicative origin. *Genome Res*, **29**, 737-749.

15. Lee, G.R., Gommers, R., Waselewski, F., Wohlfahrt, K. and O’Leary, A. (2019) PyWavelets: A Python package for wavelet analysis. *The Journal of Open Source Software*, **4**, 1237.

16. Nilsen, G., Liestøl, K., Van Loo, P., Moen Vollan, H.K., Eide, M.B., Rueda, O.M., Chin, S.F., Russell, R., Baumbusch, L.O., Caldas, C. *et al.* (2012) Copynumber: Efficient algorithms for single- and multi-track copy number segmentation. *BMC Genomics*, **13**, 591.

17. Cleal, K.A. and Baird, D. (2022) Dysgu: efficient structural variant calling using short or long reads. *Nucleic Acids Res*, gkac039.

18. Garrison, E. and Marth, G. (2012) Haplotype-based variant detection from short-read sequencing. *arXiv [q-bio]*, **1207.3907**.

19. Martin, M. (2011) Cutadapt removes adapter sequences from high-throughput sequencing reads. *EMBnet J*, **17**, 3.

20. Pedregosa, F., Varoquaux, G., Gramfort, A., Michel, V., Bertrand Thirion, Grisel, O., Blondel, M., Müller, A., Nothman, J., Louppe, G. *et al.* (2011) Sci-kit-learn: Machine Learning in Python. *Journal of Machine Learning Research*, **12**, 2825-2830.

21. Quinlan, A.R. and Hall, I.M. (2010) BEDTools: a flexible suite of utilities for comparing genomic features. *Bioinformatics*, **26**, 841-842.

22. Jia, P., Yang, X., Guo, L., Liu, B., Lin, J., Liang, H., Sun, J., Zhang, C. and Ye, K. (2020) MSIsensor-pro: Fast, Accurate, and Matched-normal-sample-free Detection of Microsatellite Instability. *Genomics Proteomics Bioinformatics*, **18**, 65-71.

23. Robinson, J.T., Thorvaldsdóttir, H., Winckler, W., Guttman, M., Lander, E.S., Getz, G. and Mesirov, J.P. (2011) Integrative genomics viewer. *Nat Biotechnol*, **29**, 24-26.

24. Kumar, R., Nagpal, G., Kumar, V., Usmani, S.S., Agrawal, P. and Raghava, G.P.S. (2019) HumCFS: a database of fragile sites in human chromosomes. *BMC Genomics*, **19**, 985.

25. Cunningham, F., Amode, M.R., Barrell, D., Beal, K., Billis, K., Brent, S., Carvalho-Silva, D., Clapham, P., Coates, G., Fitzgerald, S. *et al.* (2015) Ensembl 2015. *Nucleic Acids Res*, **43**, D662-669.

26. Subramanian, A., Tamayo, P., Mootha, V.K., Mukherjee, S., Ebert, B.L., Gillette, M.A., Paulovich, A., Pomeroy, S.L., Golub, T.R., Lander, E.S. *et al.* (2005) Gene set enrichment analysis: a knowledge-based approach for interpreting genome-wide expression profiles. *Proc Natl Acad Sci U S A*, **102**, 15545-15550.

27. Szklarczyk, D., Gable, A.L., Lyon, D., Junge, A., Wyder, S., Huerta-Cepas, J., Simonovic, M., Doncheva, N.T., Morris, J.H., Bork, P. *et al.* (2019) STRING v11: protein-protein association networks with increased coverage, supporting functional discovery in genome-wide experimental datasets. *Nucleic Acids Res*, **47**, D607-d613.

28. Quinlan, A.R. (2014) BEDTools: The Swiss-Army Tool for Genome Feature Analysis. *Current Protocols in Bioinformatics*, **47**, 11.12.11-11.12.34.

29. Rodriguez-Martin, B., Alvarez, E.G., Baez-Ortega, A., Zamora, J., Supek, F., Demeulemeester, J., Santamarina, M., Ju, Y.S., Temes, J., Garcia-Souto, D. *et al.* (2020) Pan-cancer analysis of whole genomes identifies driver rearrangements promoted by LINE-1 retrotransposition. *Nat Genet*, **52**, 306-319.
