# Supplementary material for: POLQ suppresses genome instability and alterations in DNA repeat tract lengths
Source: NAR Cancer. 2022 Jun 29;4(3):zcac020. doi: 10.1093/narcan/zcac020 (PMC9241439; doi:10.1093/narcan/zcac020)

Supplementary Figure 1

A i

```
HCT116 POLQ-/- agacaagctactattggc.aactggggacttccta
      <<<<<<<<< ||||||||||||||||||| |||||||||||||||||
Chr3:121544872 agacaagctactattggcaaactggggacttccta 121544838

    HAP1 POLQ- actgggggctggcactgctgtcaccgccg..tcccgag
      >>>>>>>>> |||||||||||||||||||||||||||||||   |||||||
Chr3:121545781 actgggggctggcactgctgtcaccgccgcttcccgag 121545818

RPE1-hTERT POLQ-/- agacaagctactattggc.aactggggacttccta
      <<<<<<<<< ||||||||||||||||||| |||||||||||||||||
Chr3:121544872 agacaagctactattggcaaactggggacttccta 121544838
```

ii

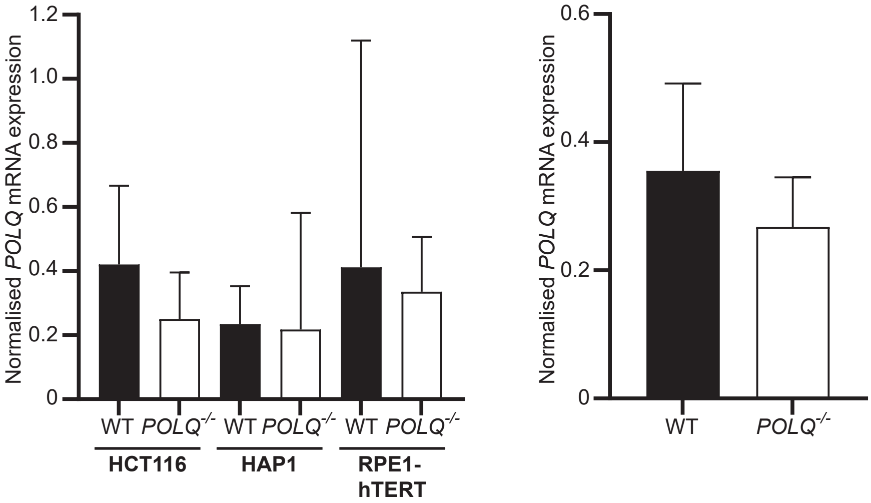

iii

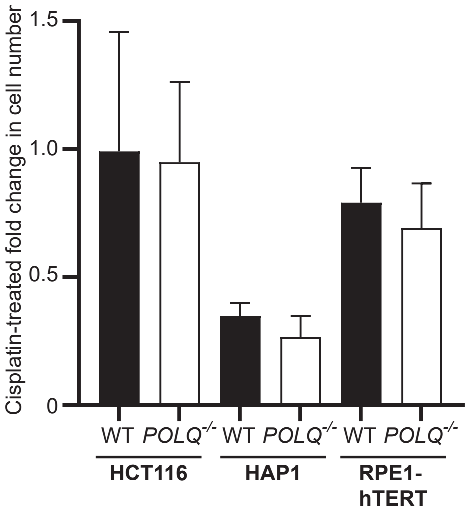

iv

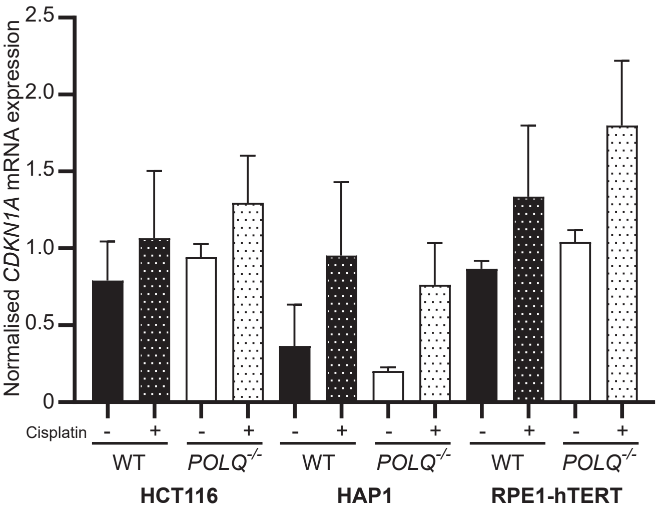

Supplementary Figure 1

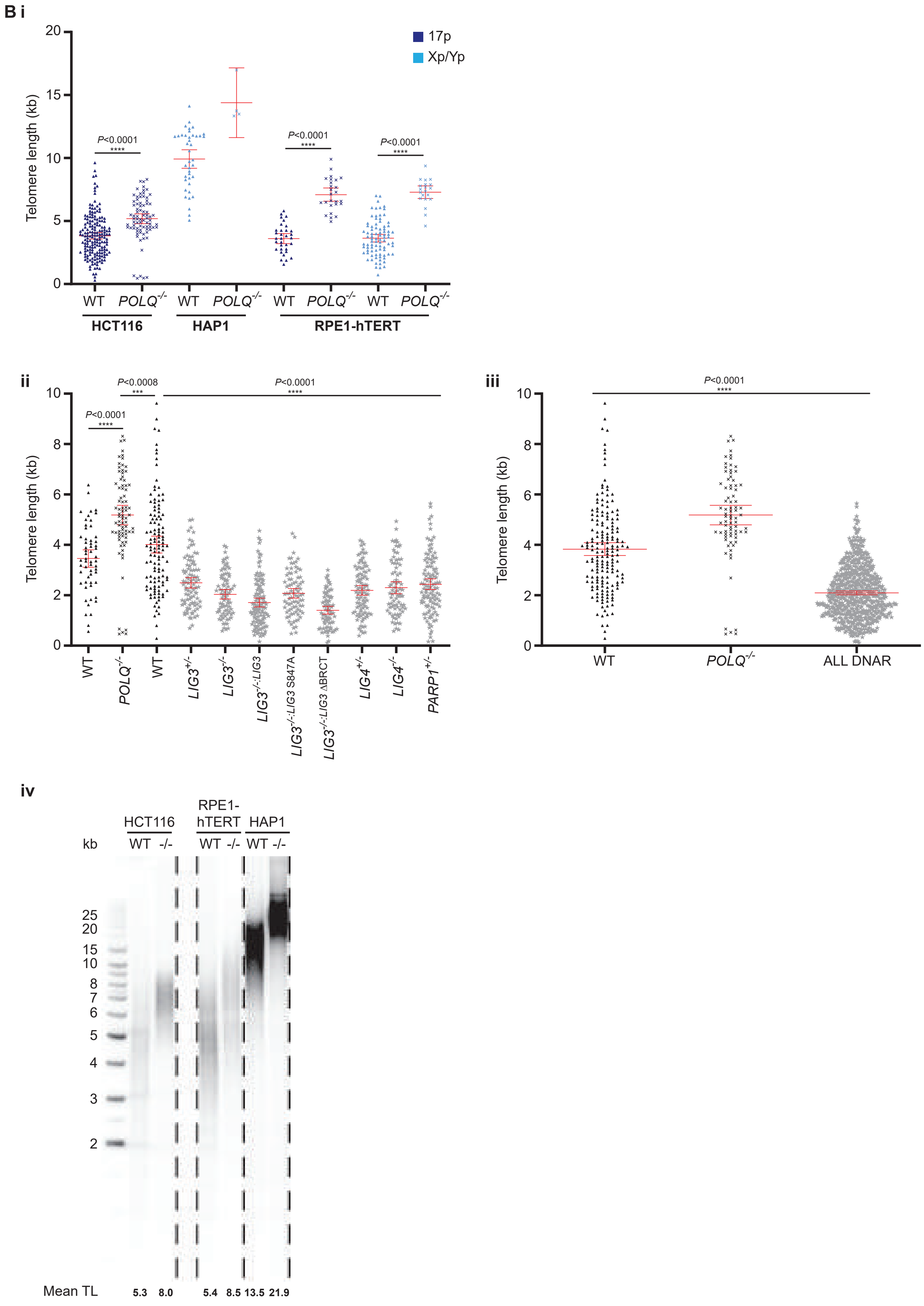

Supplementary Figure 1

C i

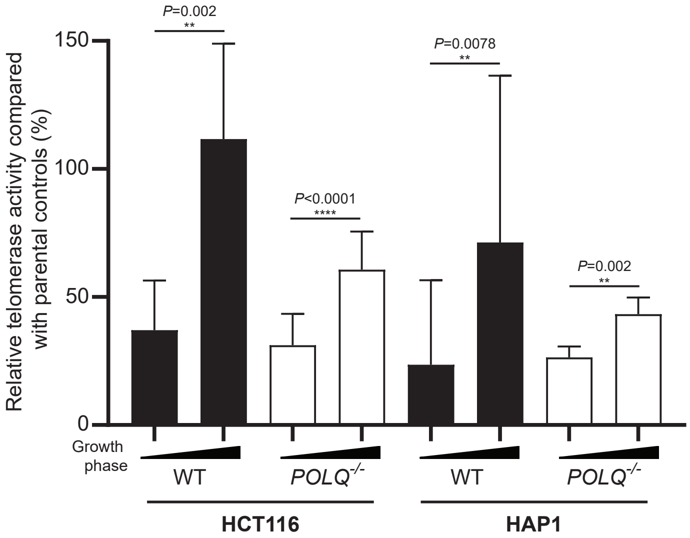

ii

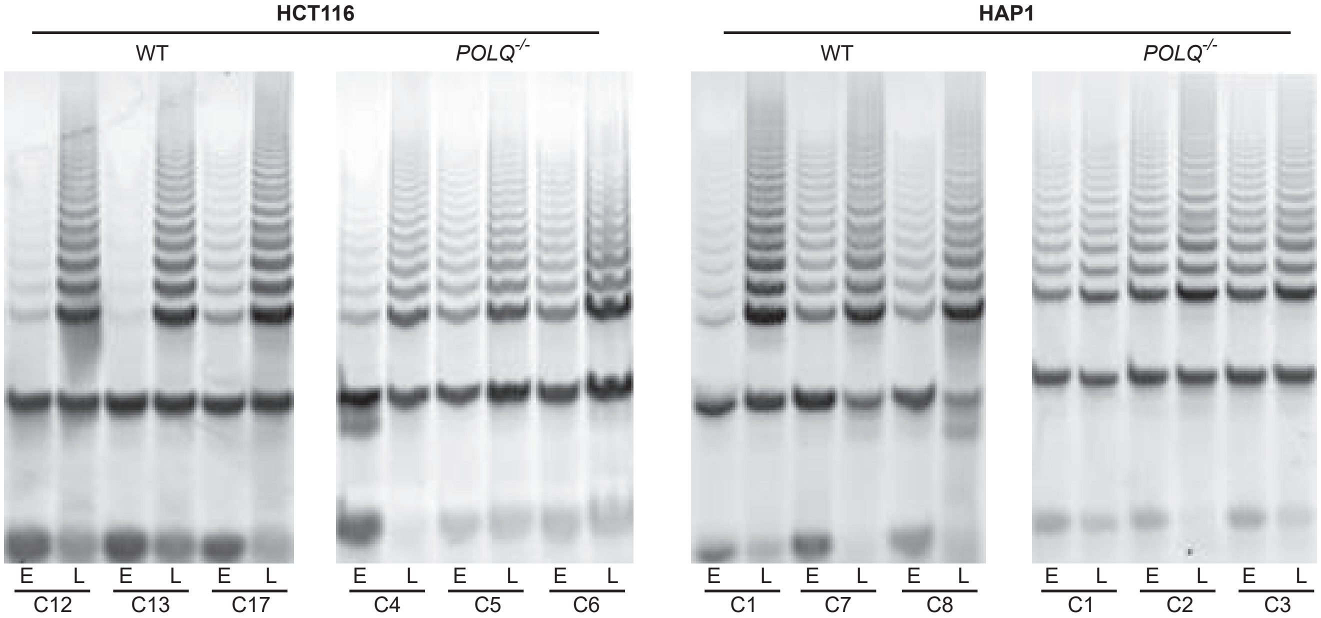

iii

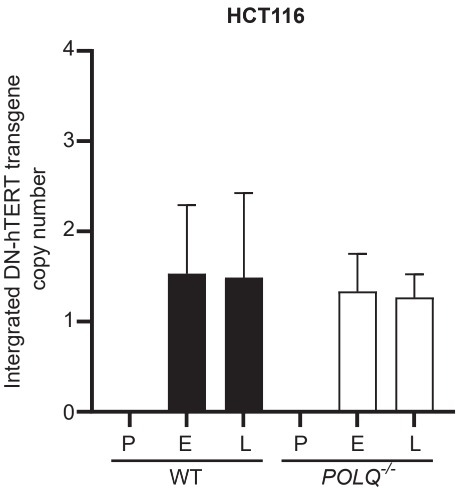

iv

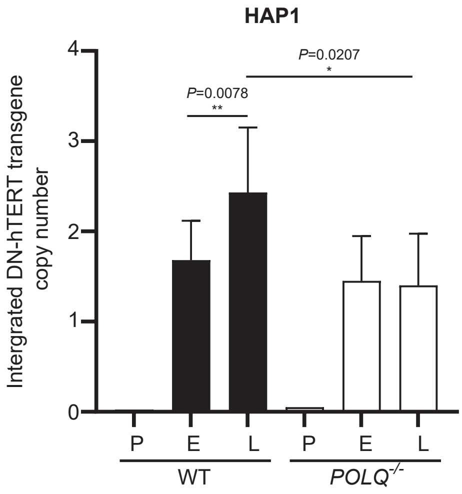

Supplementary Figure 1

D i

HCT116 WT — Growth (Population Doubling)

17p telomere length (kb)

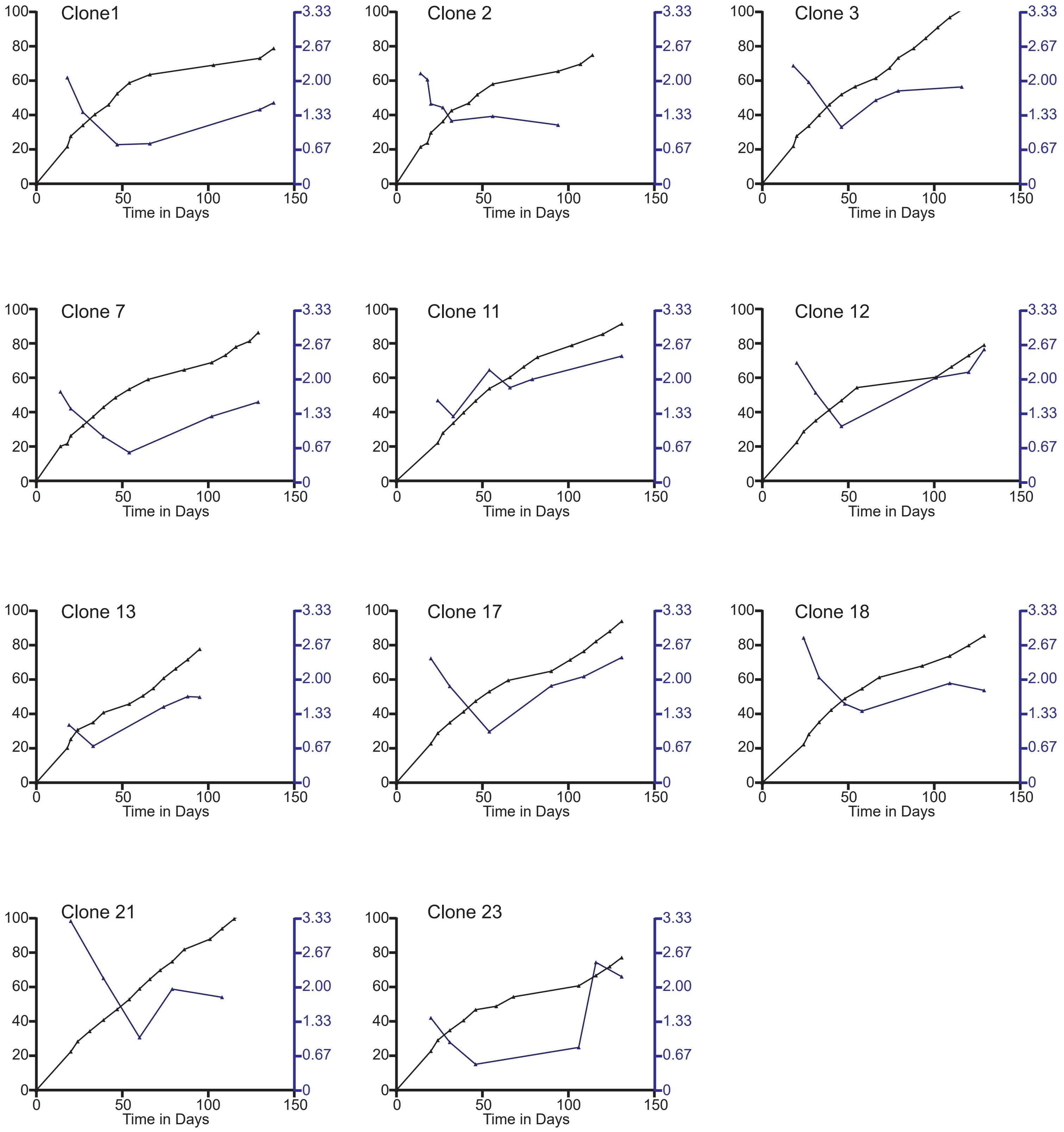

Supplementary Figure 1

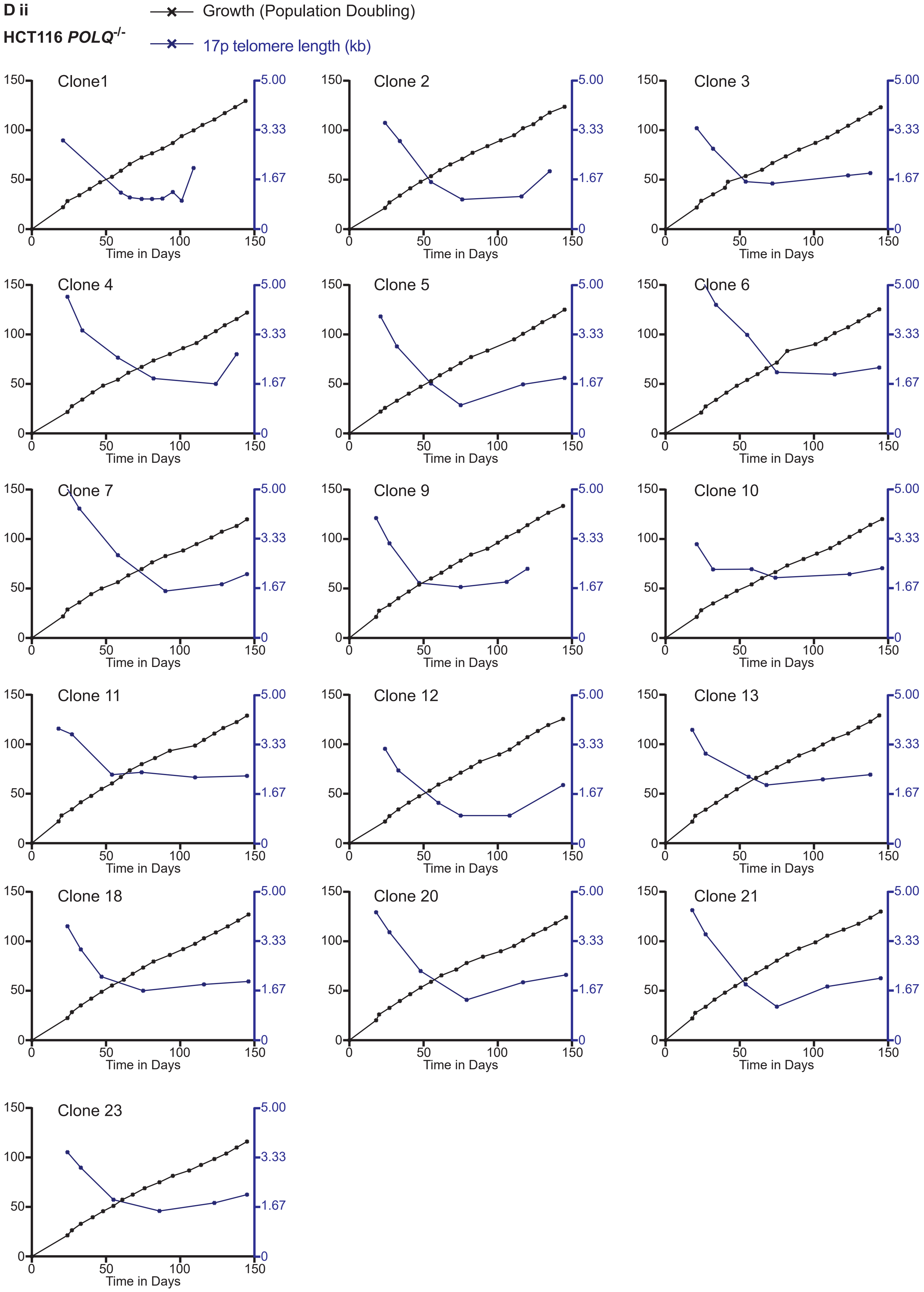

Supplementary Figure 1

D iii

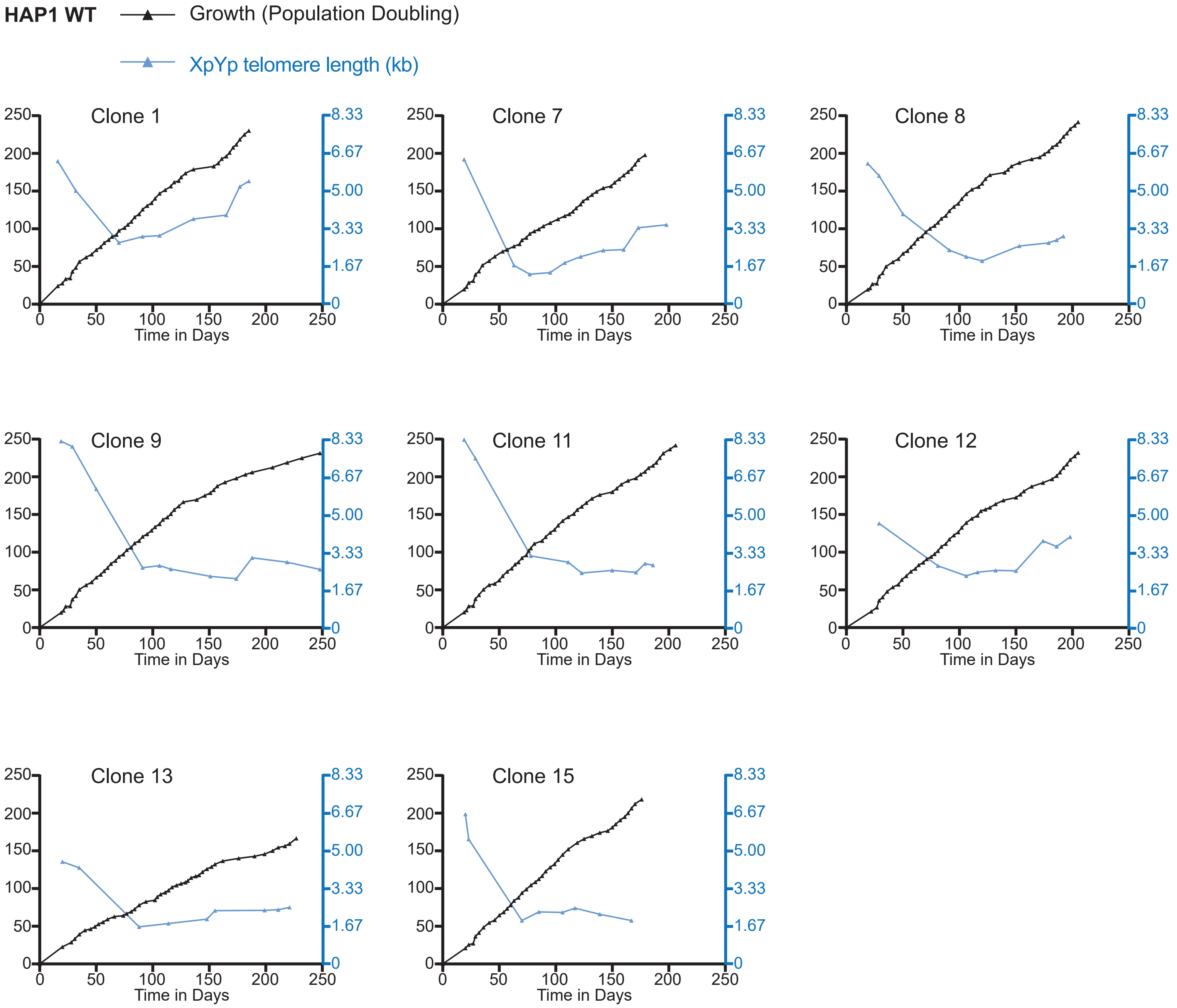

Supplementary Figure 1

D iv

HAP1 *POLQ*$^{-/-}$

—×— Growth (Population Doubling)

—×— XpYp telomere length (kb)

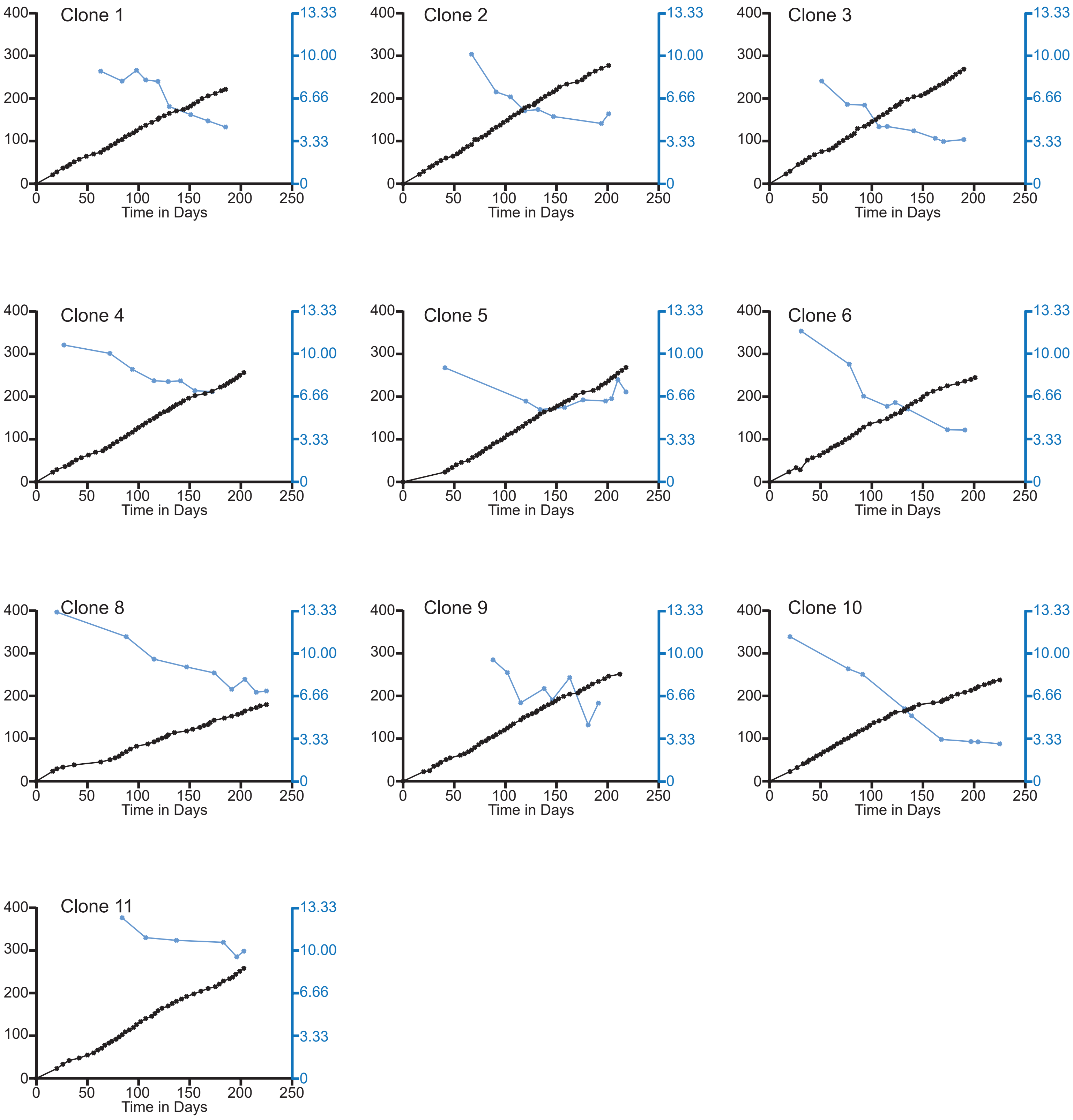

Supplementary Figure 1

E

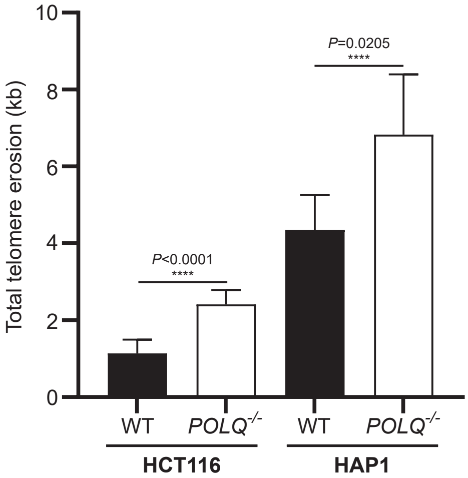

F

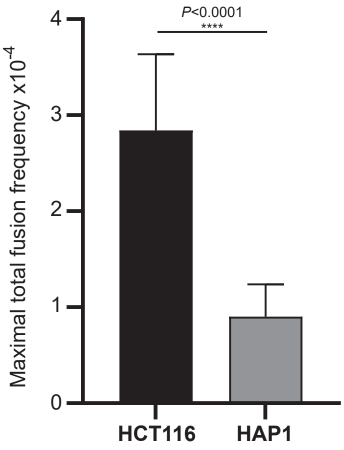

Supplementary Figure 2

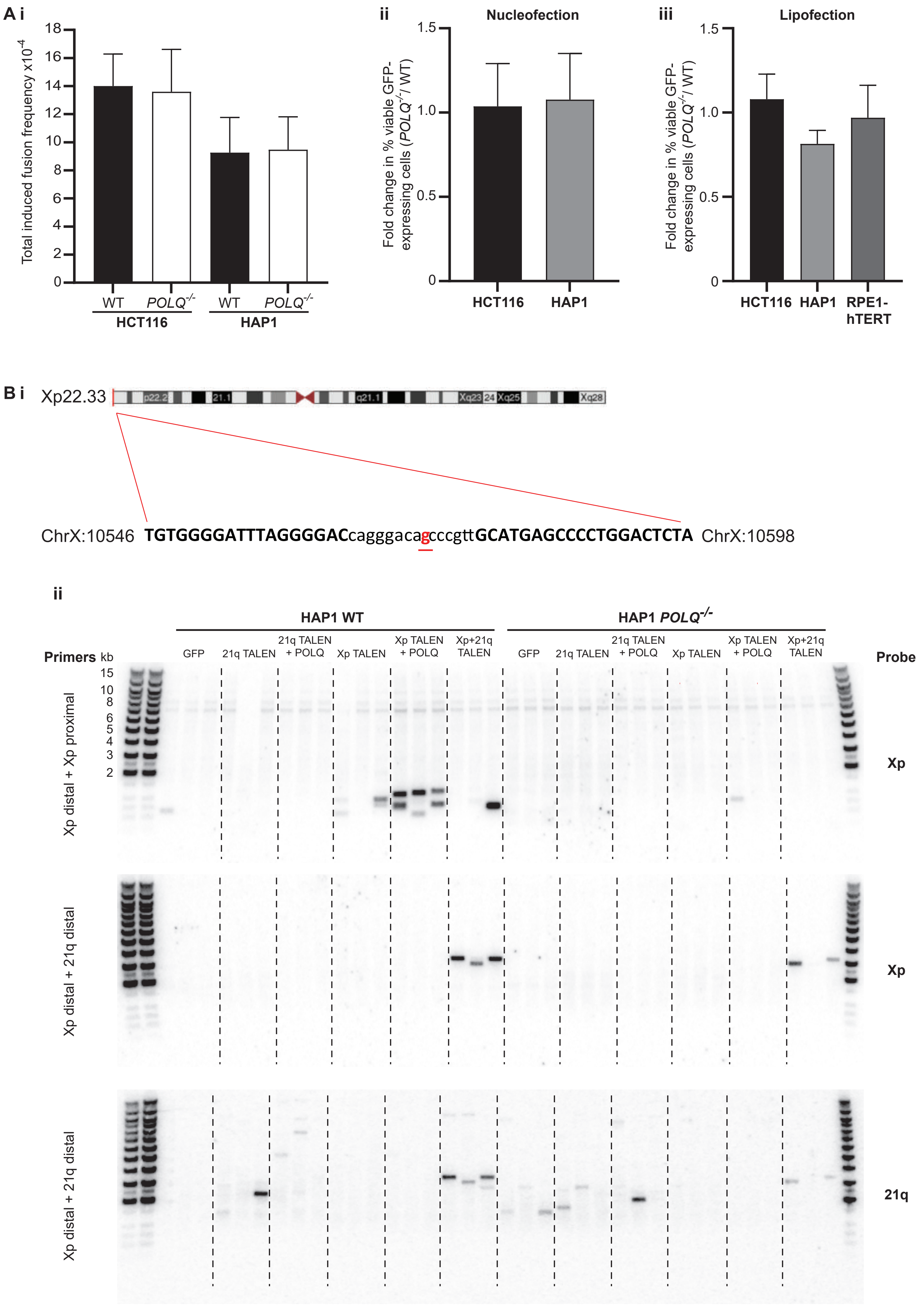

Supplementary Figure 2

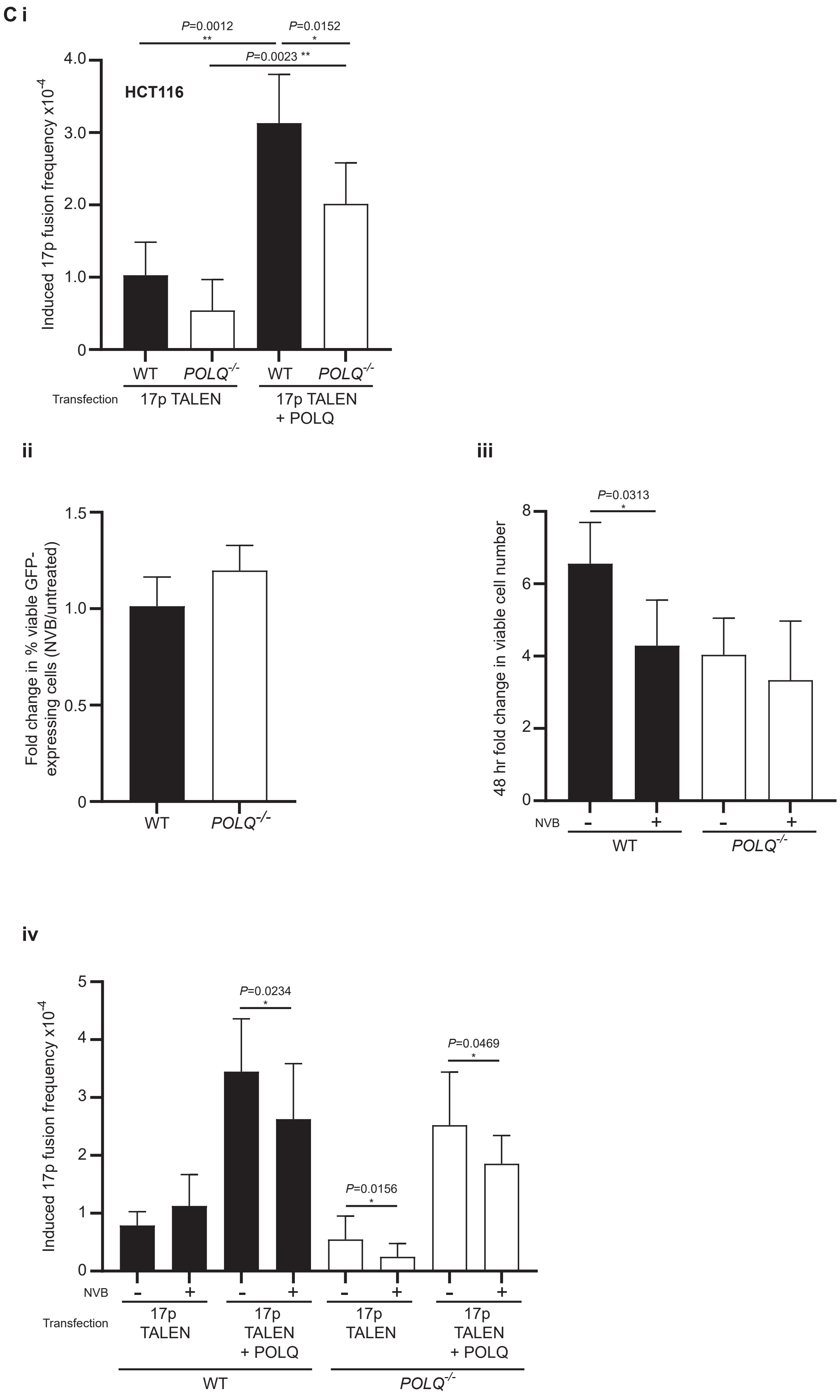

Supplementary Figure 3

A **HCT116**

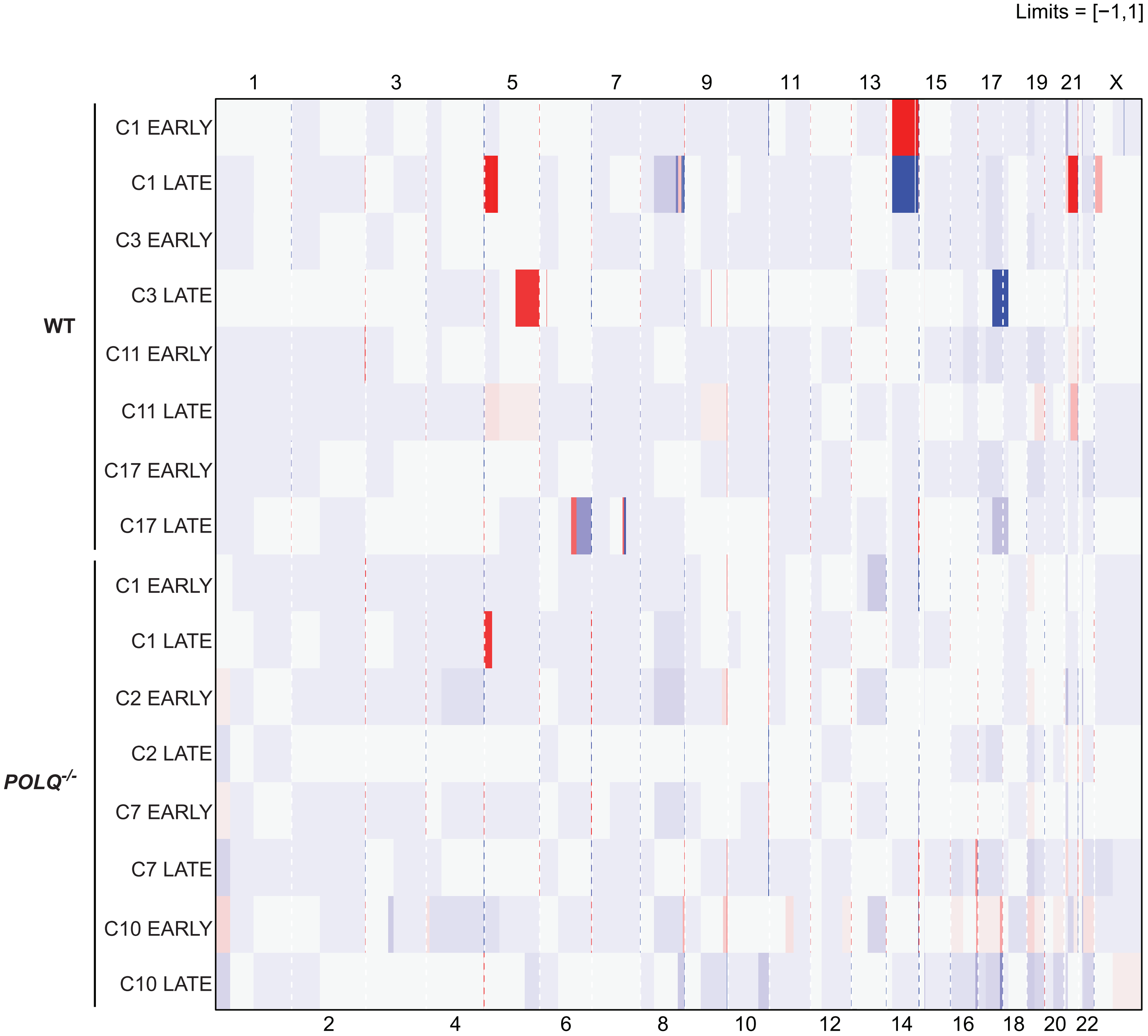

Supplementary Figure 3

B **HAP1**

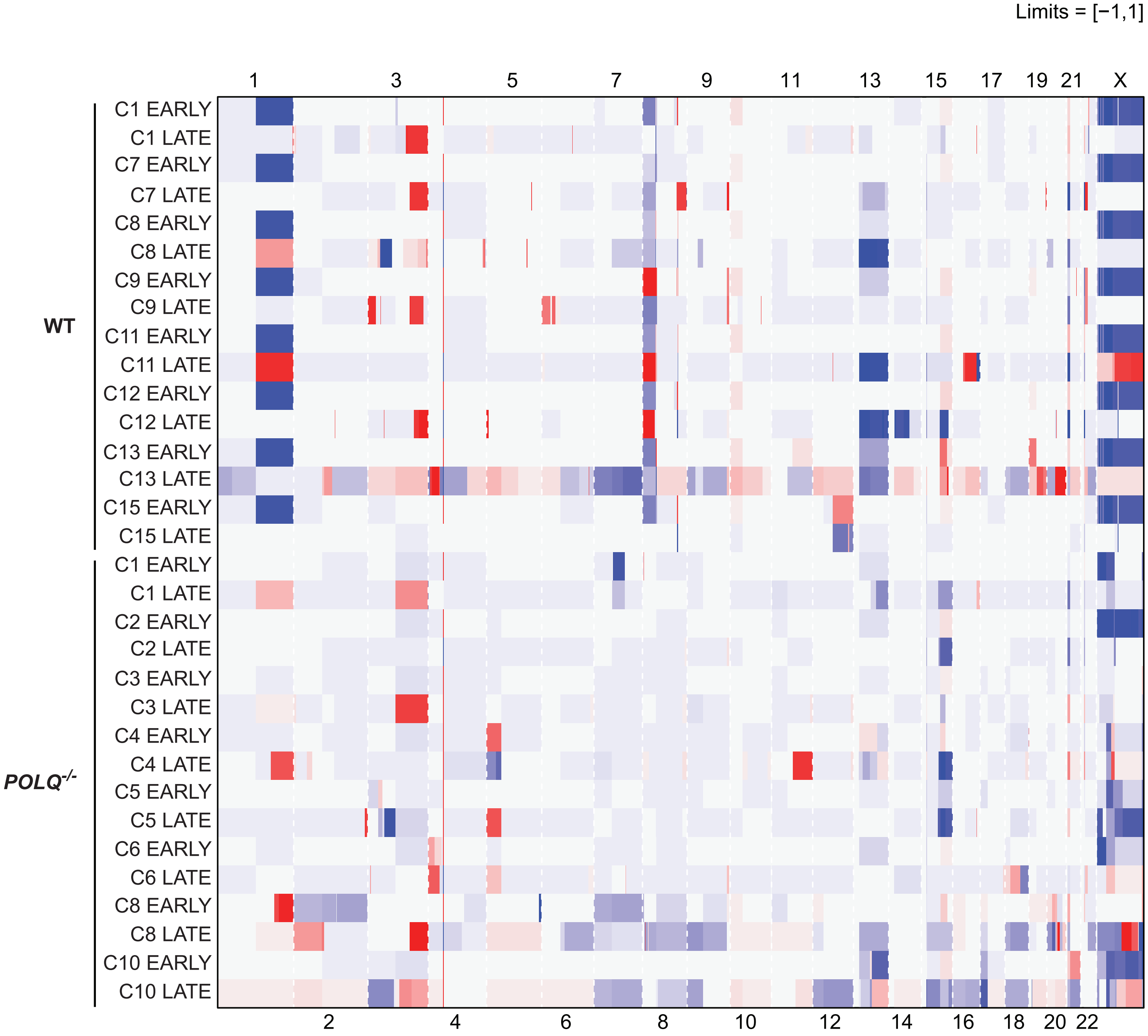

Supplementary Figure 3

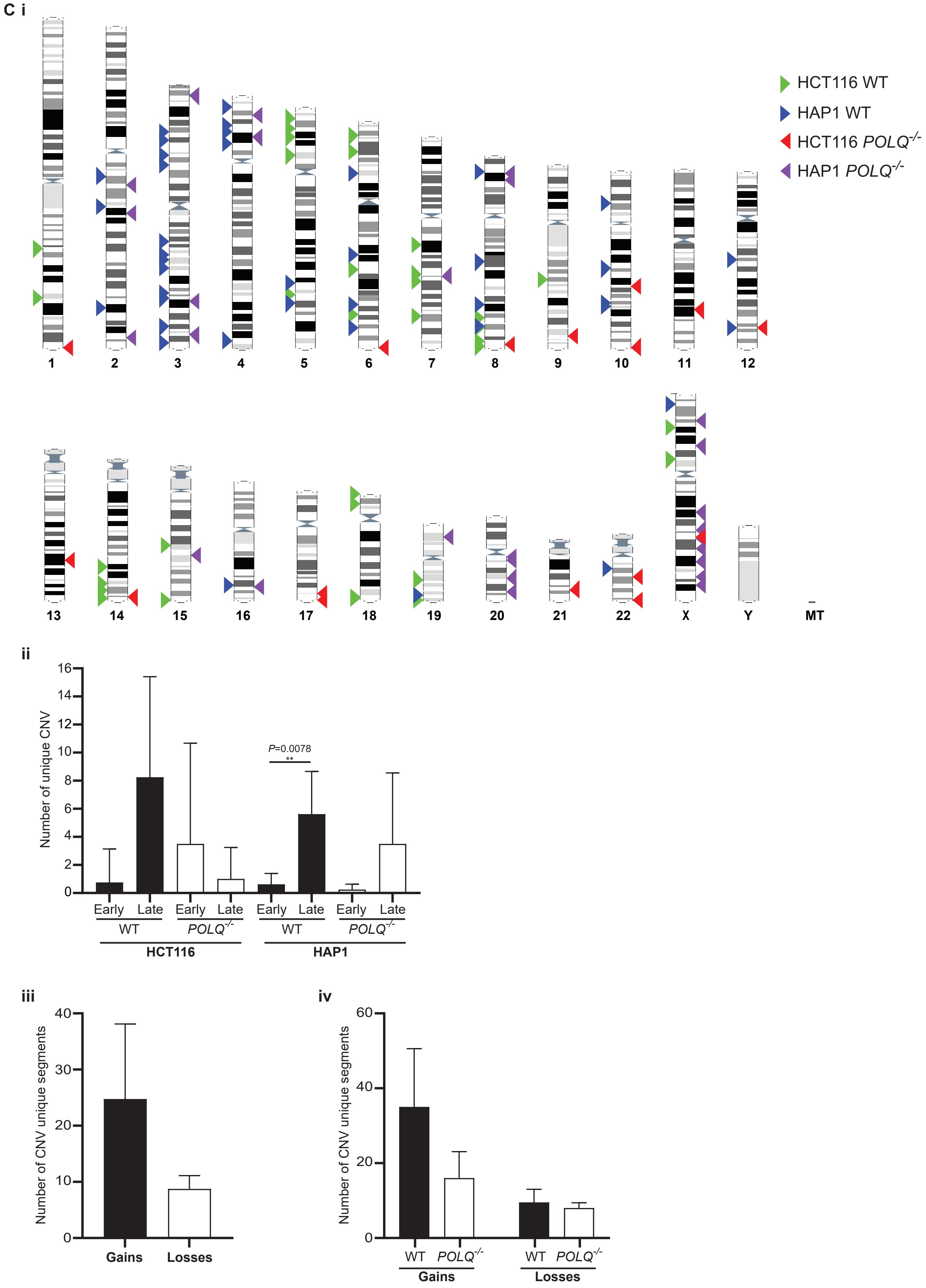

Supplementary Figure 4

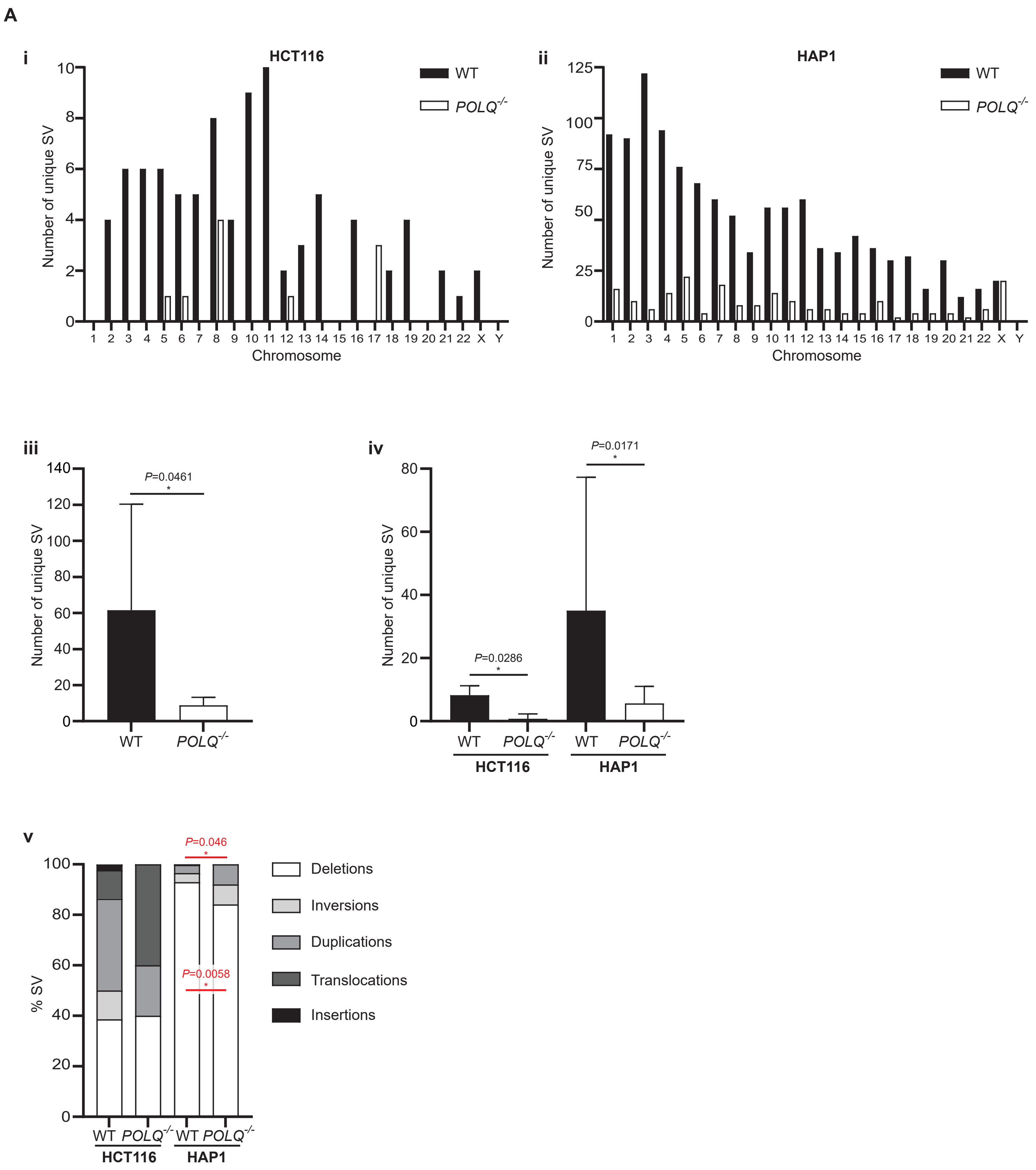

Supplementary Figure 4

B

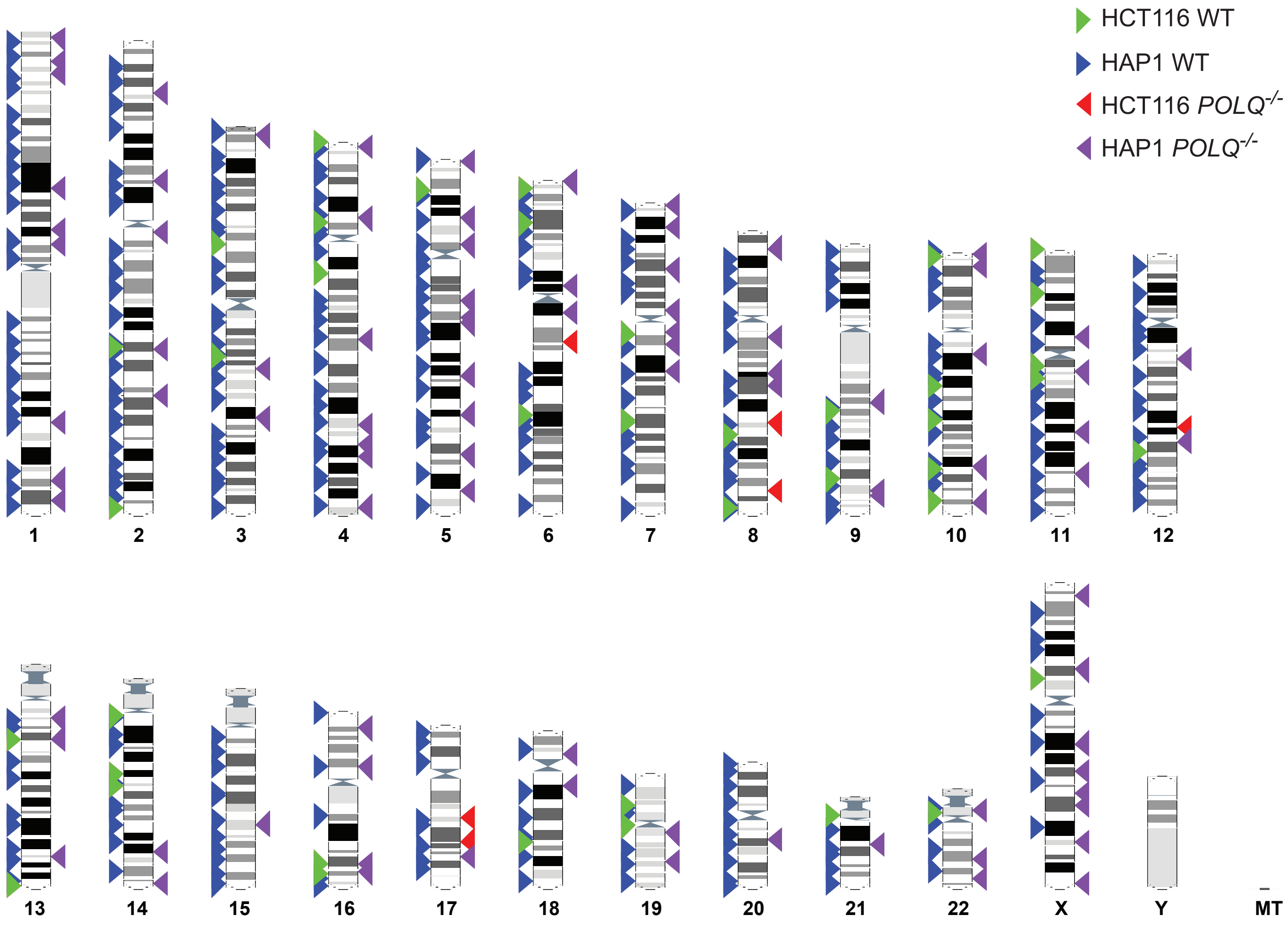

Supplementary Figure 5

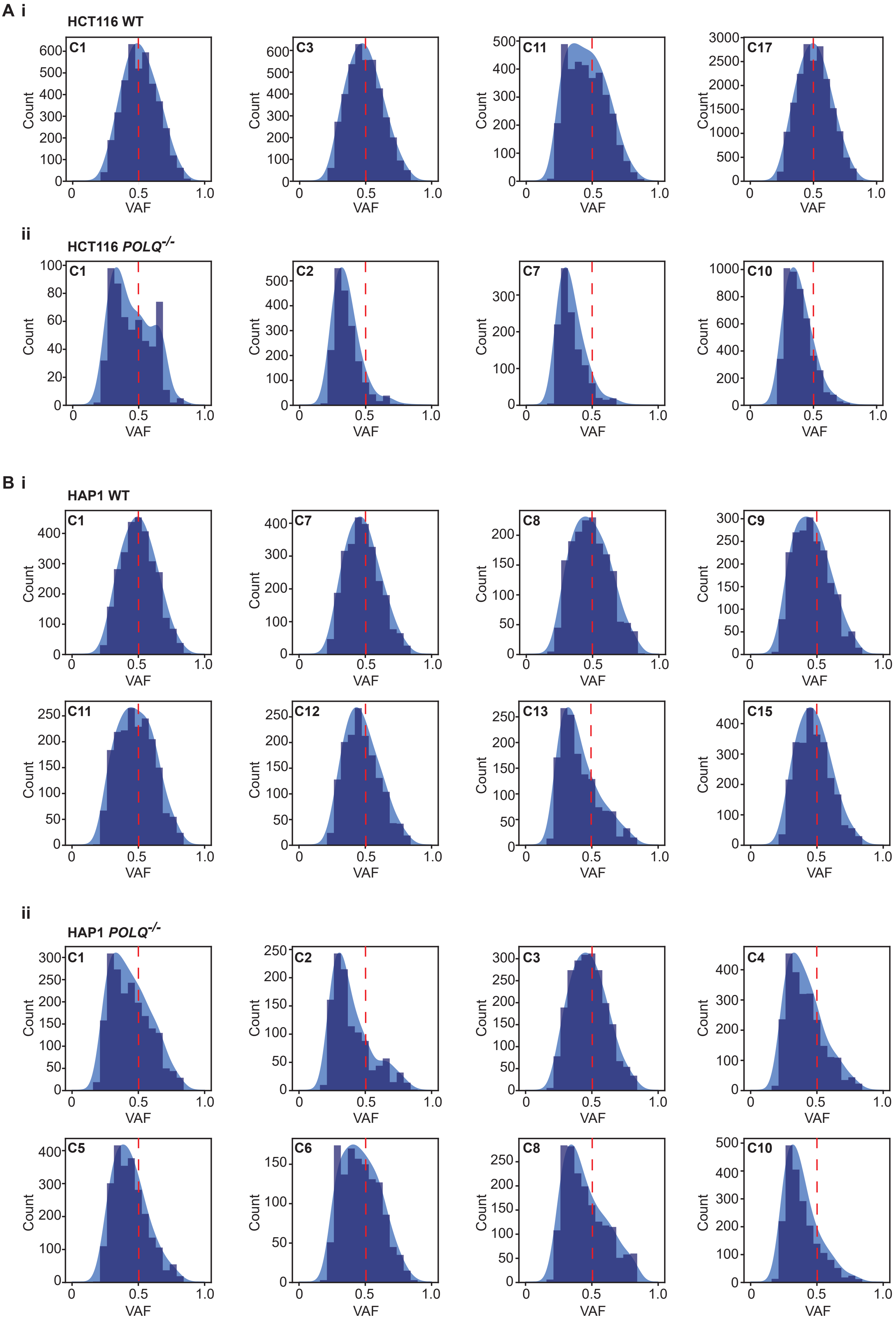

Supplementary Figure 5

C

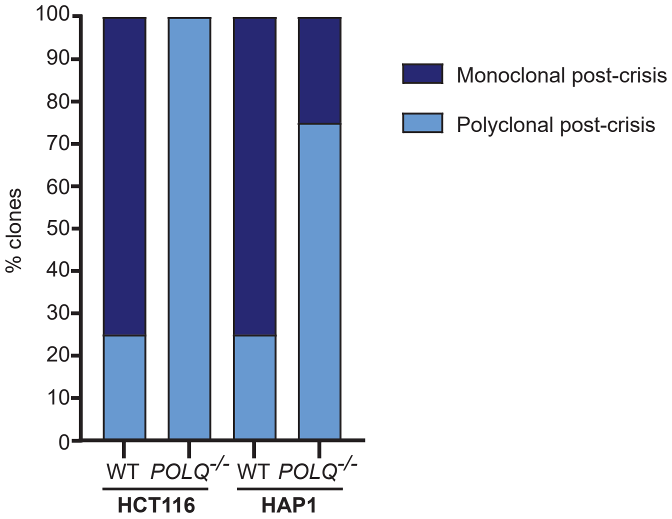

Supplementary Figure 6

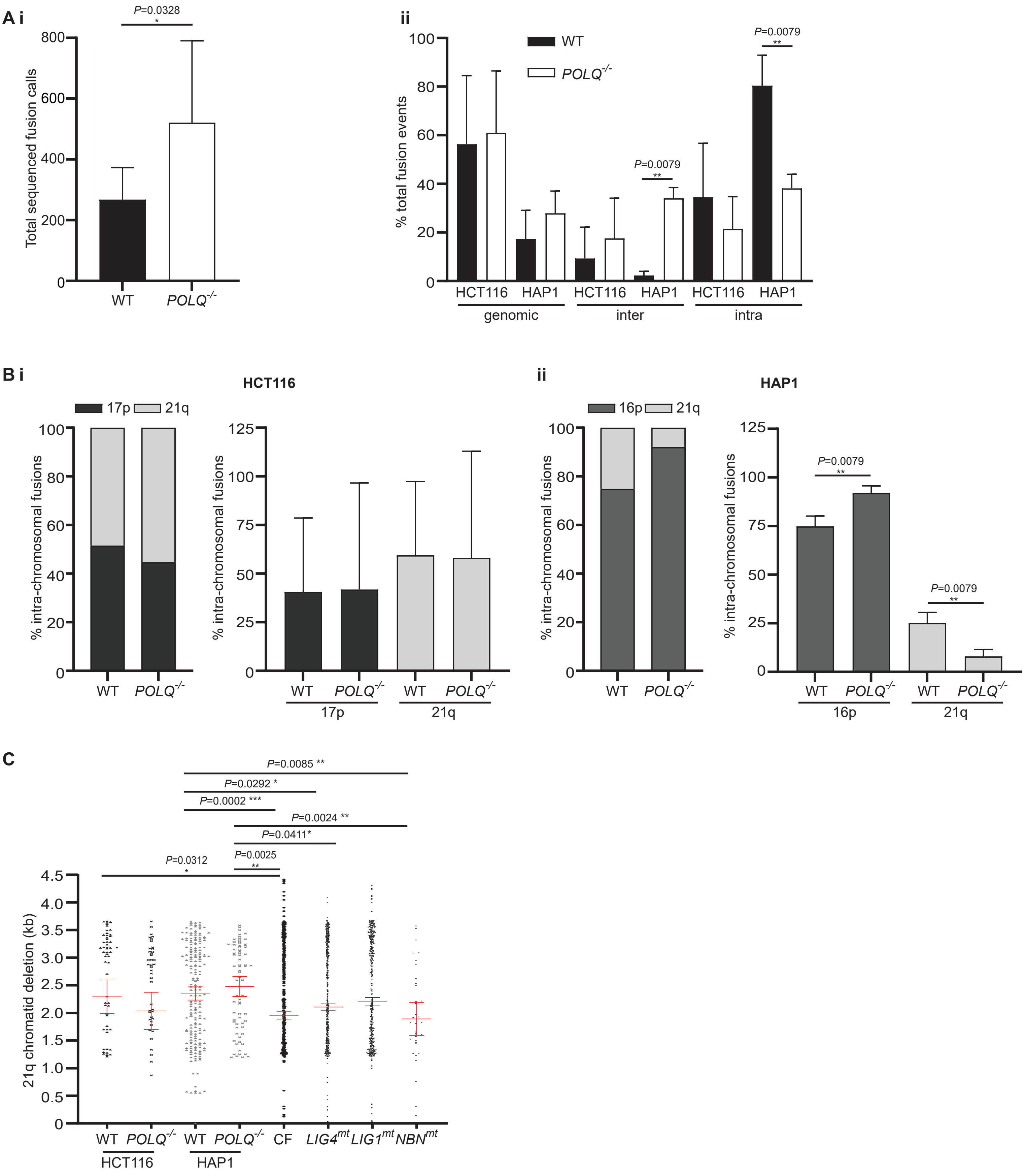

Supplementary Figure 7

A

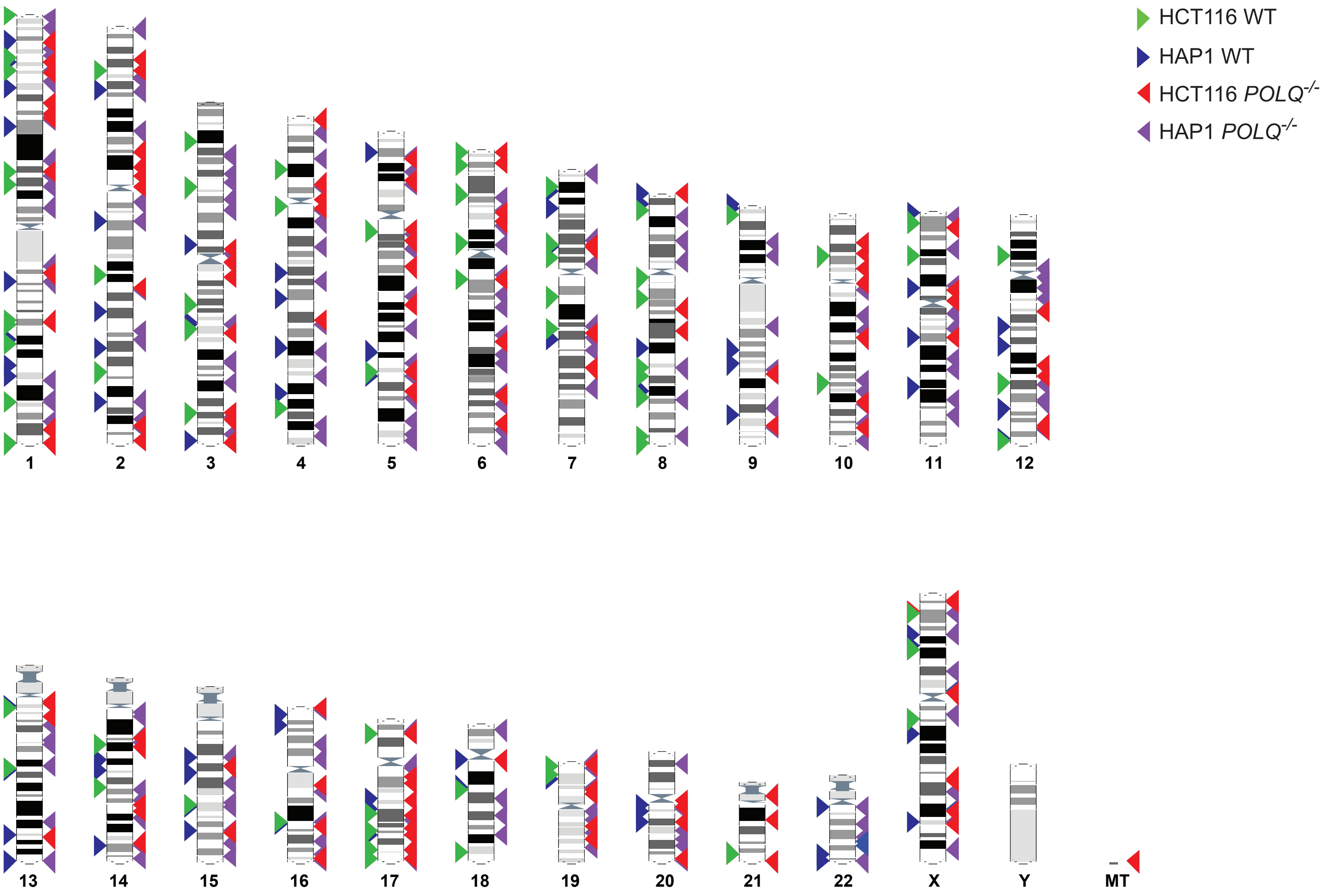

Supplementary Figure 7

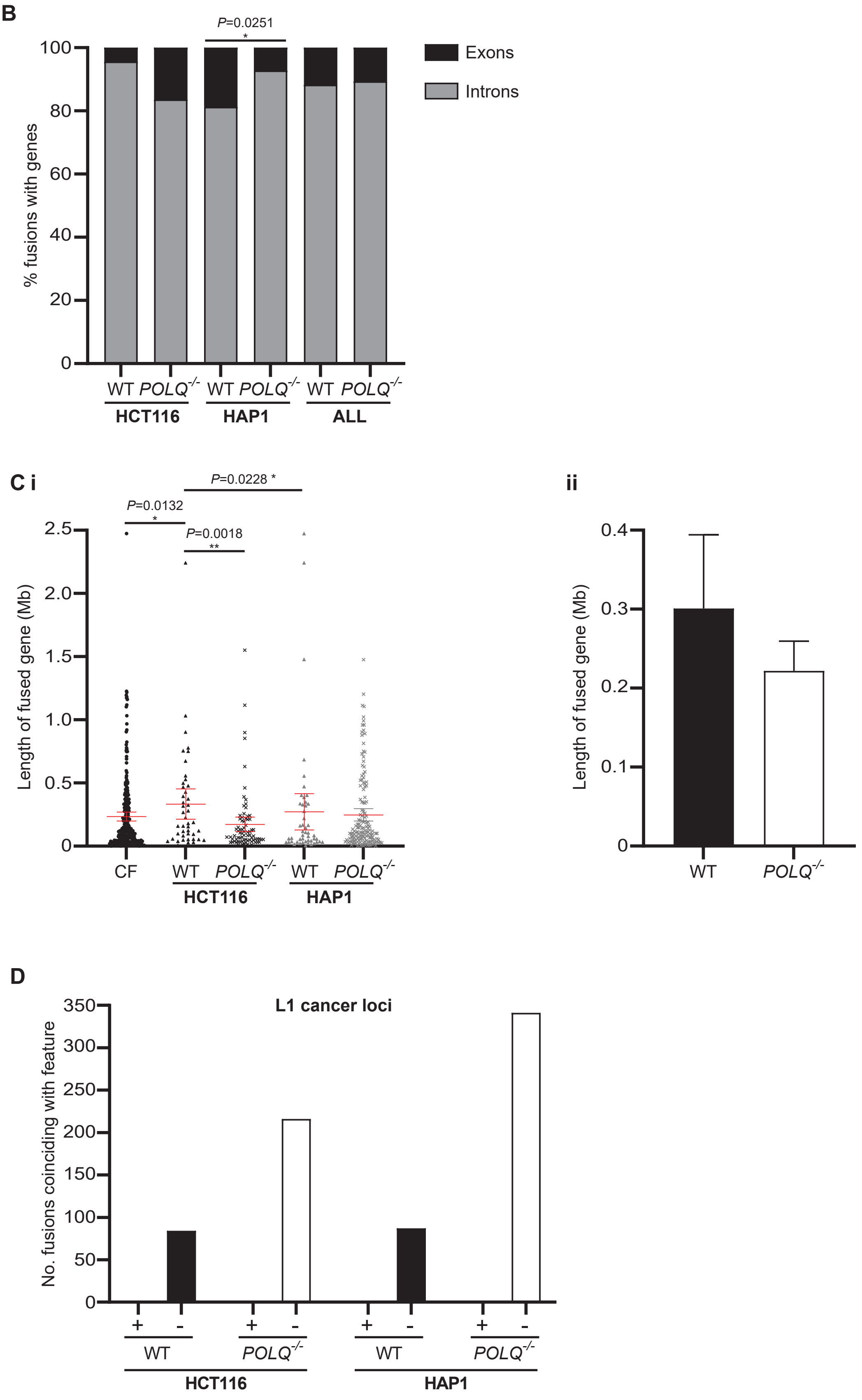

Supplementary Figure 8

A ALL WT

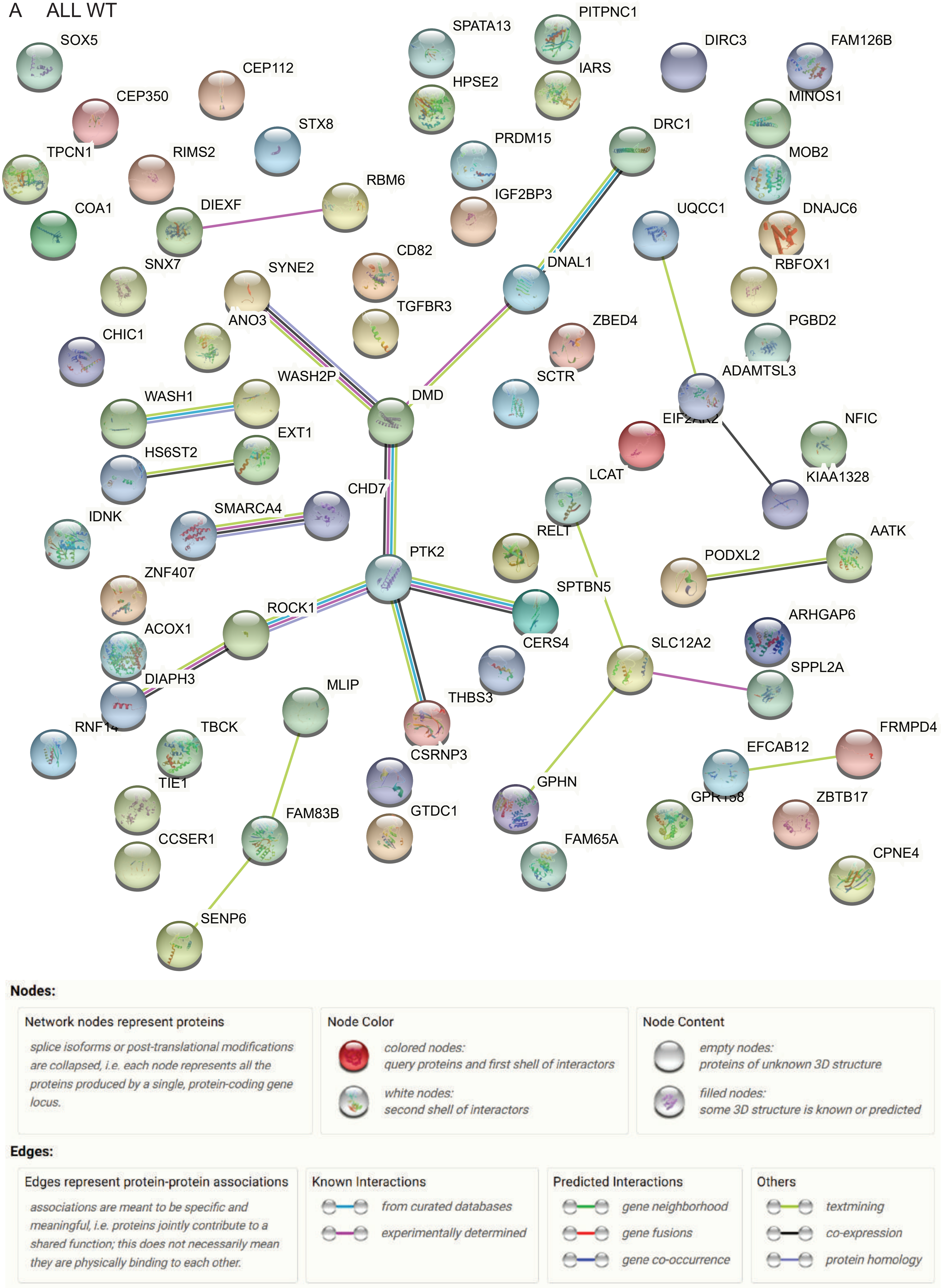

https://string-db.org/

Supplementary Figure 8

B ALL *POLQ*$^{-/-}$

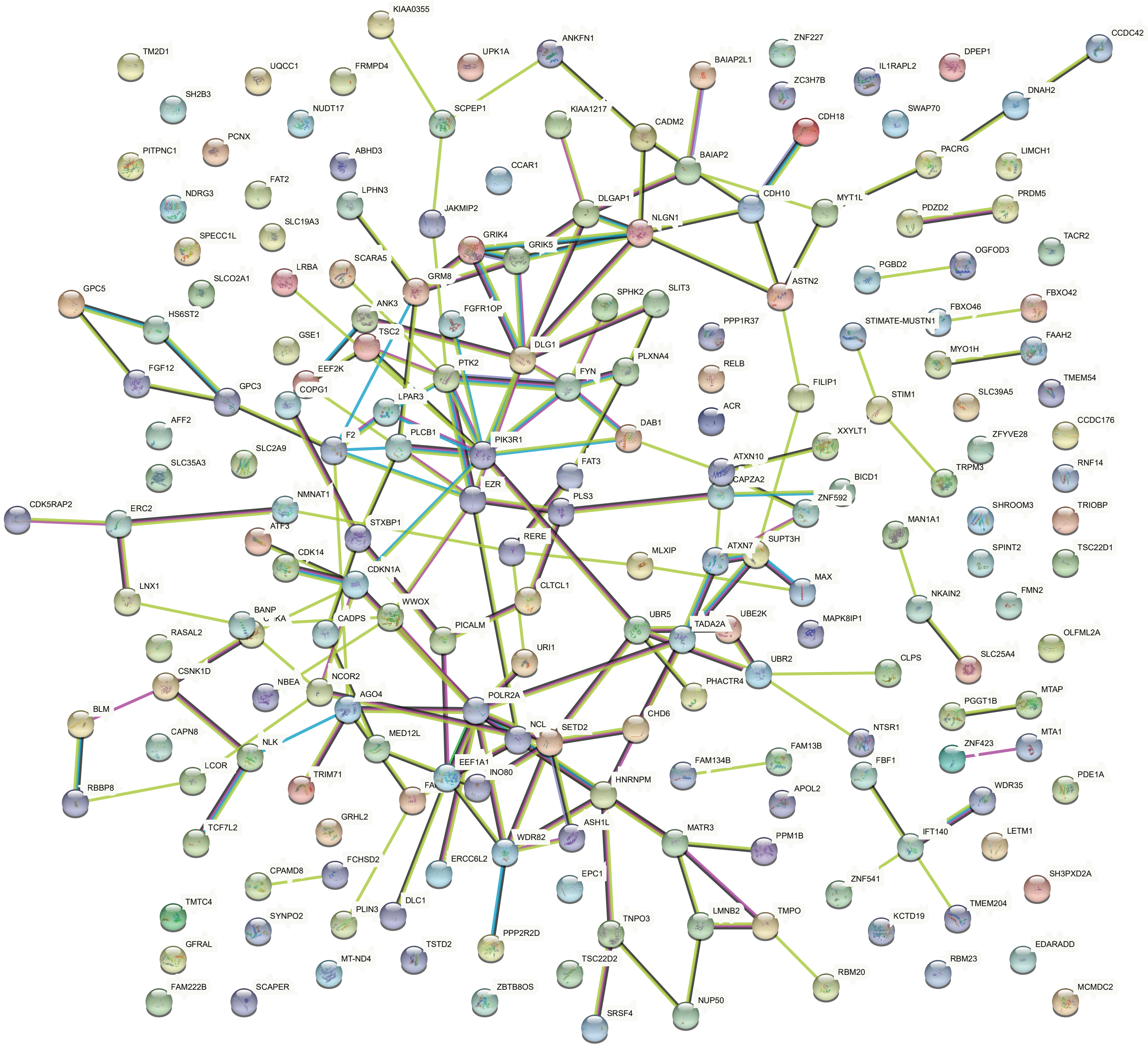

Nodes:

Network nodes represent proteins

splice isoforms or post-translational modifications are collapsed, i.e. each node represents all the proteins produced by a single, protein-coding gene locus.

Node Color

colored nodes: *query proteins and first shell of interactors*

white nodes: *second shell of interactors*

Node Content

empty nodes: *proteins of unknown 3D structure*

filled nodes: *some 3D structure is known or predicted*

Edges:

Edges represent protein-protein associations

associations are meant to be specific and meaningful, i.e. proteins jointly contribute to a shared function; this does not necessarily mean they are physically binding to each other.

Known Interactions

from curated databases

experimentally determined

Predicted Interactions

gene neighborhood

gene fusions

gene co-occurrence

Others

textmining

co-expression

protein homology

https://string-db.org/

Supplementary Figure 8

C

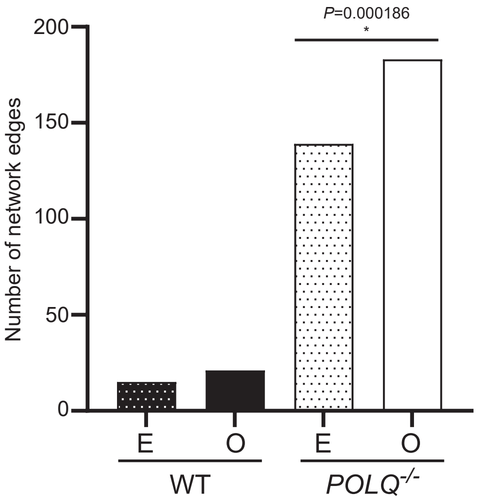

Supplementary Figure 9

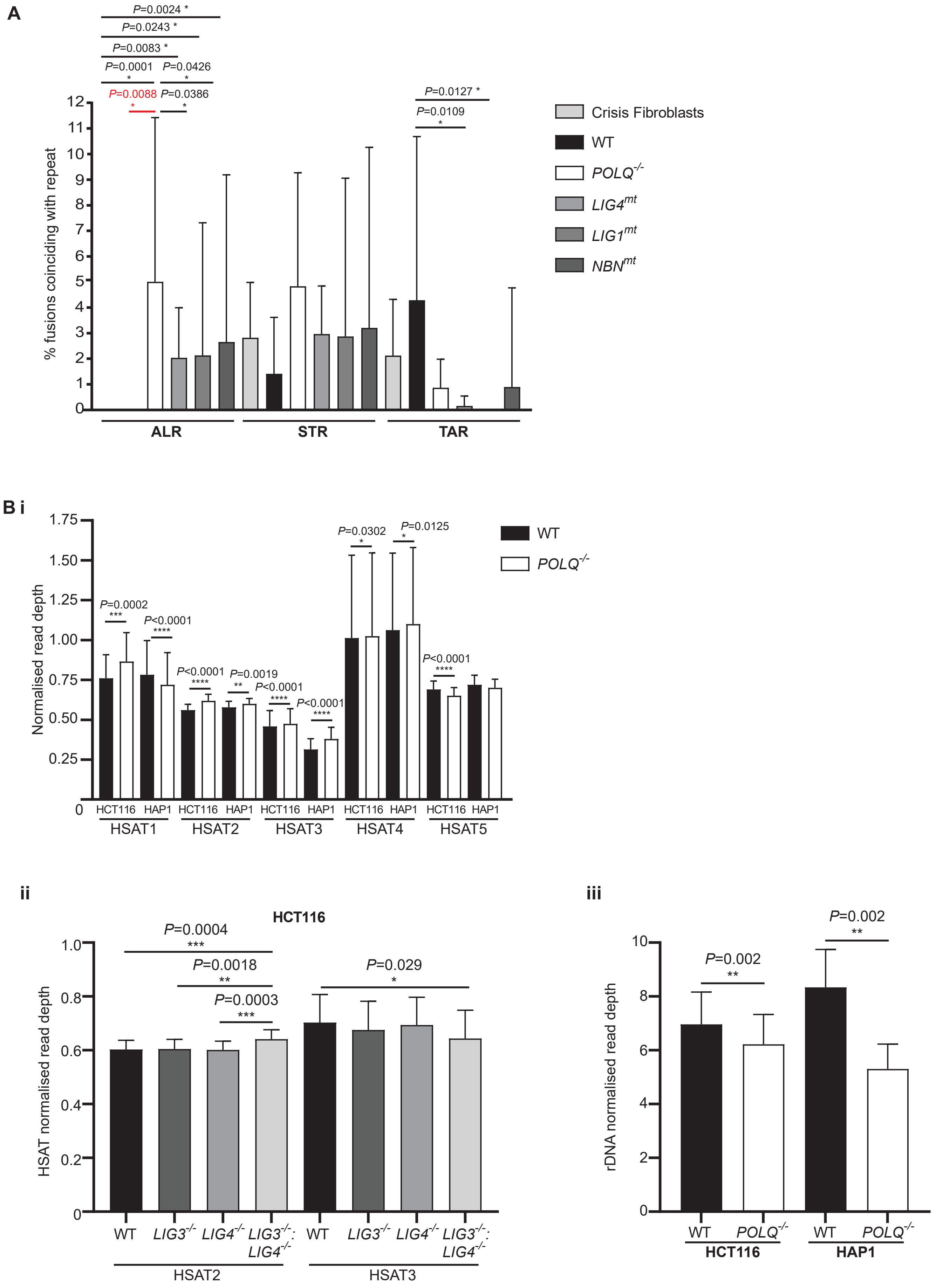

Supplement: zcac020_Supplemental_Files [file zcac020_supplemental_files.zip › POLQV6_supp_figs_190522-compressed.pdf]
